# Synthesis and Investigation of Biological Activity of New Betulonic Acid Derivatives Containing 1,2,3-Triazole Fragments

**DOI:** 10.3390/molecules29133149

**Published:** 2024-07-02

**Authors:** Roza I. Jalmakhanbetova, Gulim K. Mukusheva, Alisher Sh. Abdugalimov, Zharkyn Zh. Zhumagalieva, Wim Dehaen, Stijn Anthonissen, Yerlan M. Suleimen, Roza B. Seidakhmetova

**Affiliations:** 1 Department of Chemistry, Faculty of Natural Sciences, L.N. Gumilyov Eurasian National University, Astana 010000, Kazakhstan; aabdugalimov@gmail.com; 2 Faculty of Chemistry, Karaganda Buketov University, Karaganda 100024, Kazakhstan; mukusheva1977@list.ru; 3 Pedagogical Institute, Astana International University, Astana 010000, Kazakhstan; zharkyn.73@mail.ru; 4 Department of Chemistry, KU Leuven, Celestijnenlaan 200F, B-3001 Leuven, Belgium; wim.dehaen@kuleuven.be (W.D.); stijn.anthonissen@kuleuven.be (S.A.); 5 Department of Chemistry, Chemical Technology and Ecology, Faculty of Technology, K. Kulazhanov Kazakh University of Technology and Business, Astana 010000, Kazakhstan; syerlan75@yandex.kz; 6 Department of Clinical Pharmacology and Evidence-Based Medicine, Karaganda Medical University, Karaganda 100024, Kazakhstan; rozabat@mail.ru

**Keywords:** betulonic acid, triterpenoid, lupane, natural product, chemical transformation, synthesis, spectroscopic method, cytotoxicity, antimicrobial activity

## Abstract

The results of this study showed that the compounds synthesized by the authors have significant potential due to their antibacterial and cytotoxic properties. The apparent antibacterial activity demonstrated by the compounds suggests that they are active antimicrobial agents against common microbial pathogens that cause various socially significant infectious diseases. Compound **6** showed pronounced antimicrobial activity against the Gram-positive test strain *Staphylococcus aureus* ATCC 6538, and compound **7** demonstrated pronounced antimicrobial activity against the Gram-negative test strain *Escherichia coli* ATCC 25922 (MIC = 6.3 µg/mL). This allowed us to consider these compounds to have great potential.

## 1. Introduction

Terpenoids are a class of compounds that make up a large group of secondary metabolites, which are the most important and structurally diverse phytochemicals. These compounds are synthesized by a wide variety of plants, fungi and bacteria [1,2,3,4]. Terpenoid metabolites are responsible for many important functions in plants. They are used by plants to perform various basic growth and development functions, but most terpenoids are used for specialized chemical interactions and protection in abiotic and biotic environments. On the other hand, in other organisms, such as bacteria and fungi, terpenoids perform functions that include electron transfer, the formation of cell walls and membranes, chemical protection from predators and the establishment of symbiotic relationships [5].

It is worth mentioning the most common advanced methods of green chemistry used to extract biologically active compounds from natural sources. In recent years, recovery methods involving plant extracts instead of chemical ones have been widely used [6,7,8,9]. Additionally, microbial production of terpenoids has become an alternative source of these compounds in a process that exploits cheap raw materials obtained from biomass [10]. 

Terpenoids are of high value in the pharmaceutical, perfumery and food industries due to their biological and pharmacological properties, and they are also used as biofuels [11,12,13,14,15].

Triterpenoids and their derivatives belonging to this class are the most widespread group in nature. These compounds demonstrate a wide range of biological activities, such as anti-inflammatory [16,17], antiviral [18,19], antibacterial [20,21], antituberculosis [22], antioxidant, antifungal, physical [23] and inhibitory activities [24,25]. Recent studies have shown triterpenoids to be promising agents in the treatment and inhibition of breast cancer through the introduction of several molecular mechanisms of action on breast cancer cells [26].

The authors of [27] studied the anticancer properties of triterpenoids and found that 2,3,22,23-tetrahydroxy-2,6,10,15,19,23-hexamethyl-6,10,14,18-tetracosatrene had selective biological activity in leukemia and breast cancer cells. It was established that some new triterpenoids with fragments of pyrazole, isoxazole and piran-4-on substituted in the molecules had the most pronounced cytotoxic activity against cancer cell lines (IC_50_ = 4.31–15.6; 8.33 µm) [28].

The pharmacological activities of many betulonic acid derivatives belonging to the class of pentacyclic triterpenoids of the lupane type were determined. Thus, the antitumor activity of new betulonic acid derivatives with O- and N-propyl was studied in relation to a group of 60 human cancer cells. The compounds showed antitumor activity against most of these cells. They were more active than doxorubicin against HCT-15 colon cancer cells and ovarian cancer CA/ADDRESS [29].

The introduction of secondary amines into betulonic acid amides led to the production of derivatives with pronounced antispasmodic activity not characteristic of the triterpene skeleton [30]. C-28 imidazolides containing fragments of 3-oxo-, 3-hydroxymino- and 2-cyano-2,3-seco-4(23)-en in the lupane A ring demonstrated antitumor activity in in vitro experiments studying their antitumor activity, significantly suppressing or inhibiting the growth of lung, colon and breast cancer cells and central nervous system diseases that caused death from ovarian, prostate and kidney cancer, as well as leukemia and melanoma [31].

Cytotoxic studies of nine different human tumor cells showed that N-methylpiperazinyl amide of betulonic acid inhibited the growth of leukemia cells (SR), non-small cell lung cancer (NCI-h460) and colon cancer (hct-116) [32].

In addition, it was shown that new betulonic acid amides with piperazine derivatives had different antibacterial activities. It was also found that betulonic acid amides with a hydroxyl radical had an anticholestatic effect on mice, i.e., when piperidine nitroxide was introduced into the lupane nucleus, its hepatoprotective activity increased [33,34].

Continuing research on the modification of triterpenoids in order to study the biological activity, we synthesized new derivatives of betulonic acid.

## 2. Results and Discussion

### 2.1. Chemistry

Betulonic acid is a lupane-type triterpenic acid with a wide range of biological activities. The presence of reaction centers, such as hydroxy, keto, carboxyl and double bonds, in the composition determines the possibilities of chemical modification of molecules of this triterpenoid. Its chemical modification can lead to the formation of compounds whose activity exceeds that of the natural precursor. For example, the introduction of a triazole fragment into the composition of a molecule led to derivatives with higher biological activity [35,36,37,38,39,40]. Compounds with a wide range of biological activities synthesized on the basis of betulonic acid are also known [41,42,43,44,45].

In order to expand a number of biologically active compounds while continuing work on the modification of triterpenoids, reactions based on betulonic acid were carried out. 

**Scheme 1:**
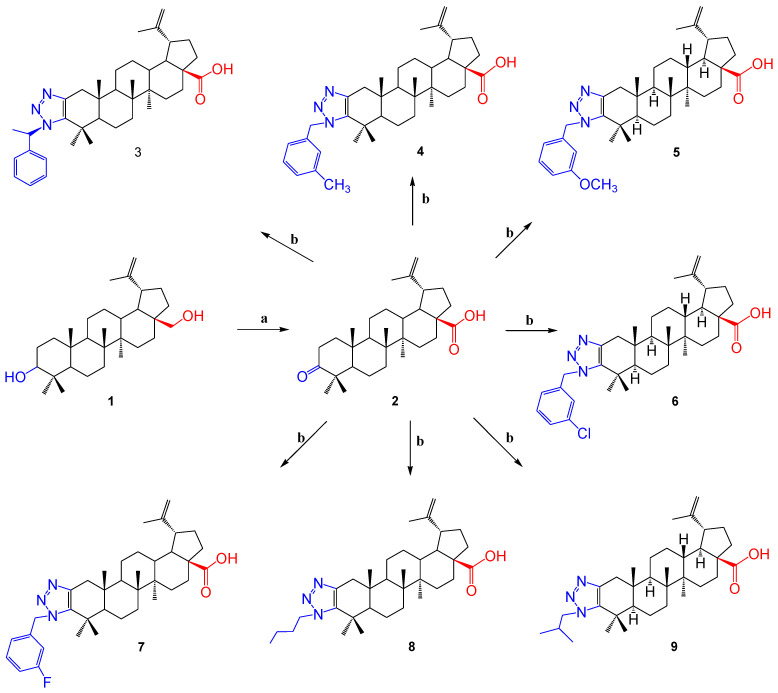
Reagents and conditions: (a) Jones reagent, acetone, RT; (b) primary amine, 4-phenylnitroazide, toluene, 24 h, 100 °C.

The first series of derivatives was synthesized in accordance with the sequence of reactions shown in Scheme 1.

For triazolization, primary aromatic (containing chloro, fluoro, methyl and methoxy groups in the aromatic ring), linear (butylamine) and branched (isopropyl) amines were taken. The column chromatography method was used to isolate the formed products. The yield of the obtained products **3**–**9** was 60–68%.

The yield was calculated for the substance obtained after column chromatography. Each stage in the separation of the resulting product was carefully carried out, and no loss of product occurred. It was observed that a relatively high-yield product was formed by the interaction of betulonic acid **2** with 3-methylbenzylamine (68%). Compared with other products (chloro, fluoro, butyl and propyl), there were no sharp differences in yields, with the exception of methyoxy-group-containing compound **3** and methylphenylamine-containing product **5**.

In previous work [46], the mechanisms of the triazole formation reaction were shown, indicating two alternative discoveries of the triazoline intermediate cycle. The role of 4-nitrophenylazide in this reaction is explained by the fact that it formally acts as a diazotransfer agent, with the formation of 4-nitroaniline as a by-product.

Spectroscopic (^1^H and ^13^C NMR) and mass spectrometric methods were used to establish the obtained compounds. In the ^1^H NMR spectra of the synthesized betulonic acid triazole derivatives **3**–**7**, the signals of the triazole residue were shown in the area δ 7.33–5.61 p.m., and in the spectrum of the **8** and **9** compounds it was observed that additional signals were formed in comparison with the initial molecule; the signals were clearly shown in the area δ 2.91, 4.29 p.m. for **8** and δ 2.92, 4.09 p.m. for **9**. Loss of the characteristic signal of the carbonyl group at the C-3 position of the initial molecule in the ^13^C NMR spectra of the resulting compounds provided evidence of a triazolation reaction. The molecular ion peak [M + H]^+^ was present in the mass spectra of the compounds. 

In order to continue obtaining new derivatives of betulonic acid, the triazolation reaction was carried out in the presence of ammonium acetate, as a result of which compound **10** was obtained. Then, the compound was dissolved in methanol and brombutane and potassium tret-butoxide (*t*ButOK) were added to it. As a result, a mixture of two substances (**11** and **12**) was formed. In this case, it was observed that a carboxyl-group proton exchange situation occurred. The yield of the formed compounds was 46 and 20%, respectively.

**Scheme 2:**
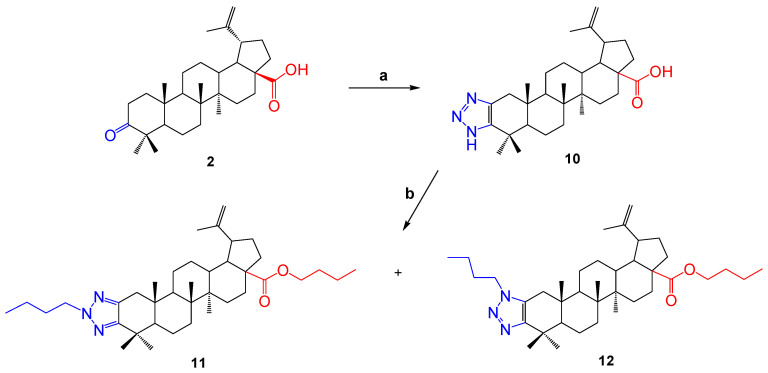
Reagents and conditions: (a) ammonium acetate, 4-nitrophenyl azide, DMF, 80 °C, 24 h; (b) *t*ButOK, 1-bromobutane, methanol, 60 °C, 12 h.

The compounds **11** and **12** were separated using column chromatography. The structure of the synthesized compounds was determined by NMR spectroscopy and mass spectrometry. By analyzing the spectral data and taking into account the molecular ionic peak in the mass spectra of the compounds, it was proved that the molecules had the structures shown in Scheme 2. In the ^1^H NMR spectrum of compound **11**, the signals of the protons of the exomethylene group at the C-20 position were present in the area δ 4.76 and 4.63 p.m., and in compound **12** they were present in the area δ 4.75 and 4.61 p.m. The presence of a butyl residue at C-28 was evidenced by the shift of the carbonyl group signal in the ^13^C NMR spectrum to a strong field compared to molecule **10**. 

**Scheme 3:**
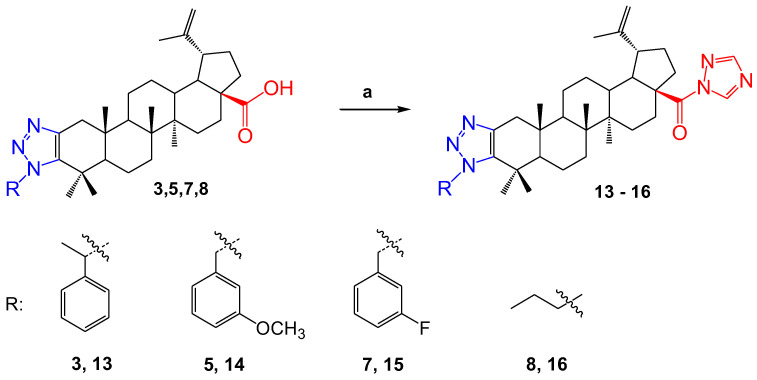
Reagents and conditions: (a) CDT, THF, 70 °C.

In order to study the biological activity, the betulonic acid derivatives **3**, **5**, **7**, and **8** were chemically modified. The reactions were carried out by reacting 1,1’-carbonyldi-(1,2,4-triazole) in THF. As a result, colorless powdery substances **13**–**16** were obtained (Scheme 3). The yield of the products was very high, with the exception of compound **14**, the yield of which was 52%.

In the ^1^NMR spectra of the compounds, protons in the triazole ring at the C-28 position were observed as singlets at δ 8.93 and 7.99 p.m. for **13**, at δ 8.92 and 7.99 p.m. for **14** and **15**, and at δ 8.93 and 8.00 p.m. for **16**. In the ^13^C NMR spectra, it was observed that, due to the formation of a triazole ring in place C-28, the signal of the carbonyl group shifted to a weak field, that is, it was manifested in regions δ 173.38, 173.39, 173.38 and 173.39 p.m., respectively.

**Scheme 4:**
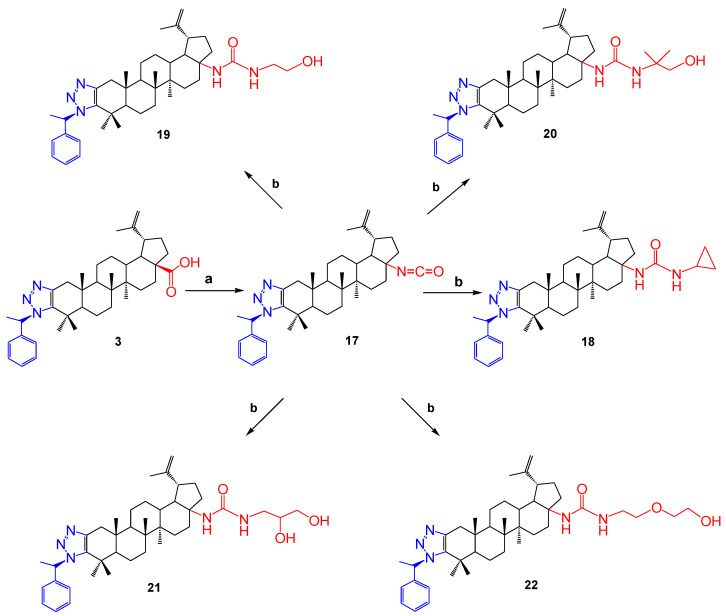
Reagents and conditions: (a) DPPA, toluene, Et_3_N; (b) amine, toluene, 110 °C.

Continuing further modifications of the triazole derivatives of betulonic acid, compound **3** reacted with diphenylphosphoryl azide (DPPA) in toluene in the presence of triethylamine. The resulting carbamide derivative **17** was included in the further reaction. Then, the corresponding amines were added. As a result, the high-yield colorless powder substances **18**–**22** were obtained (Scheme 4).

The structure of the synthesized compounds was determined by spectroscopic and high-resolution mass spectrometry methods. 

All synthesized compounds were tested for antimicrobial and cytotoxic activity.

### 2.2. Evaluation of the Biological Activity 

#### 2.2.1. Antimicrobial Activity

The antimicrobial activity of the samples was studied on recommended reference test microorganisms, facultative anaerobic Gram-positive cocci *Staphylococcus aureus* ATCC 6538, aerobic Gram-positive spore-forming *Bacillus subtilis* ATCC 6633, Gram-negative bacillus facultative anaerobes *Escherichia coli* ATCC 25922, aerobic *Pseudomonas aeruginosa* ATCC 27853 and k yeast fungus *Candida albicans* ATCC 10231, by serial dilution with determination of the minimum inhibitory concentration (MIC) [47,48]. The test strains of microorganisms used in the study were obtained from the American Type Culture Collection. 

The results of the study of the antimicrobial activity of the samples by serial dilution are shown in Table 1.

As a result of the antimicrobial study, it was found that the tested compounds exhibited antibacterial activity against opportunistic test strains to varying degrees. An analysis of the antimicrobial activity of the tested substances showed that its manifestation depends on the type of pathogen. The test strains of *Staphylococcus aureus* and *Escherichia coli* were most sensitive to all the compounds presented.

It was revealed that compound **6** showed pronounced antimicrobial activity against the Gram-positive *Staphylococcus aureus* test strain ATCC 6538 and that compound **7** showed pronounced antimicrobial activity against the Gram-negative *Escherichia coli* test strain ATCC 25922, their MICs being 6.3 µg/mL. At the same time, their antimicrobial activity was comparable to the activity of the comparison drug ceftriaxone, the MIC of which was also 6.3 µg/mL, and exceeded the activity of the comparison drug benzylpenicillin sodium salt against staphylococcus by two times and that against *E. coli* by three times.

Compounds **3** and **18** showed moderate antibacterial activity against both the Gram-positive test strain *Staphylococcus aureus* ATCC 6538 and the Gram-negative test microorganism *Escherichia coli*, their minimally inhibitory concentrations being 12.5 µg/mL in relation to the test strains.

The Gram-negative *Pseudomonas aeruginosa* test strain proved to be the most resistant to the action of these compounds. None of the tested compounds, except for compound **7**, which had a weak MIC = 50 µg/kg, showed antibacterial properties against this microorganism.

Samples **4**, **7**, **8**, **13**, **15**, **18**, **19** and **21** showed moderate antibacterial activity against the Gram-positive test strain *Staphylococcus aureus* ATCC 65; their MICs were 12.5 and 25 µg/mL.

Compounds **2**, **3**, **5**, **7**, **19** and **20** showed a slight antifungal effect against the yeast-like fungus *Candida albicans* ATCC 10231 at concentrations of 25–50 µg/mL, which were lower than the activity of the comparison drug nystatin (MIC = 12.5 µg/mL).

Thus, among the new synthesized derivatives **3**–**10** and **13**–**22** were compounds with antibacterial activity comparable to the activity of the drug ceftriaxone and exceeding the activity of benzylpenicillin sodium.

Compound **6** turned out to be the most active against *Staphylococcus aureus*, while compound **7** was the most active against the Gram-negative test microorganism *Escherichia coli*. This allowed us to consider these compounds as very promising for the search for new antibacterial drugs, though further in-depth research is required.

#### 2.2.2. Cytotoxic Activity

These new synthesized compounds were subjected to the *Artemia salina* (Leach) lethality test. Larvicidal activity based on the percentage of larval mortality was evaluated after 24 h exposure to the treatments.

According to Meyer et al. [49], who classified substances into toxic (LC_50_ value < 1000 mcg/mL) and nontoxic (LC_50_ value > 1000 mcg/mL), almost all the tested compounds showed good cytotoxic activity against artemia compared to the reference compound.

The cytotoxicity of the compounds was evaluated by means of the survival test of larvae of *Artemia salina* (Leach) crustaceans under in vitro cultivation conditions [49,50]. The results are shown in Table 2.

The study showed that samples of the tested compounds **2**–4, **6**–**10**, **13**–**15**, **18**–**20** and **22** exhibited cytotoxic activity against larvae of the marine crustaceans *Artemia salina* (Leach).

Since the test for mortality of shrimp in brine has a strong correlation with tests for cytotoxicity against human cancer cells, it could be used to screen for antitumor potential. The aforementioned samples could be considered potential candidates for an antitumor compound of plant origin. It was found that these compounds can be sources of cytotoxic and antitumor compounds. Of course, it is necessary to conduct studies to assess their antitumor activity using human cancer cell lines in order to identify their true potential for therapeutic use.

The results obtained during this study showed that the synthesized compounds had significant potential due to their antibacterial and cytotoxic properties. Among the synthesized compounds, it was found that **6** and **7** had promising antibacterial properties and exhibited cytotoxicity.

The pronounced antibacterial activity demonstrated in this study for some compounds indicated them to be potential antimicrobial agents active against common microbial pathogens that cause various socially significant infectious diseases.

If we consider the relationship between the “structure” and the “biological activity” of the synthesized compounds, when triazole fragments were introduced into the structures of the molecules, the antimicrobial activities of the derivatives in relation to the Gram-positive and Gram-negative strains were relatively different. It was found that the activity of chlorine-containing triazole derivative **6** (MIC = 6.3 µg/mL) against the Staphylococcus aureus strain was significantly higher—about eight times that of the initial betulonic acid **2** (MIC = 50 µg/mL). And it was noticed that the activity of compounds **3**, **4**, **7** and **8** (MIC = 25 µg/mL) increased two times in relation to this strain compared to the original molecule. The fluorinated triazole derivative **7** (MIC = 6.3 µg/mL) showed very high antimicrobial activity in relation to the Escherichia coli strain, while the original betulonic acid **2** showed no activity against this strain. In addition, compounds **3**–**6** (MIC = 12.5–50 µg/mL), namely, phenyl-, methyl-, methoxy- and chlorine-containing triazole derivatives, also showed activity against this strain (Escherichia coli); the initial betulonic acid **2** was resistant to this strain, i.e., it did not show activity. Of the synthesized compounds shown in Scheme 1, only the fluorinated triazole derivative **7** was active against the *Pseudomonas aeruginosa* strain and the *Candida albicans* fungus. As for the fungus *Candida albicans*, the activity of compound **7** (MIC = 25 µg/mL) was twice as high as that of betulonic acid **2** (MIC = 50 µg/mL).

If we consider the relationship between the “structure” and the “biological activity” of the triazole derivatives of betulonic acid **13**–**16** obtained by further conversion (shown in Scheme 3), there was a decrease in biological activity in relation to all strains compared with the initial compounds **3**, **5**, **7** and **8**. It was noted that the antimicrobial effect of the fluorinated triazole derivative **15** (MIC = 12.5 µg/mL) in relation to the *Staphylococcus aureus* strain was twice as high as that of the initial compound **7** (MIC = 50 µg/mL).

Considering the relationship between “structure” and “biological activity” in the N-substituted triazole derivatives **17**–**22** (shown in Scheme 4), it was noticed that antimicrobial activity in relation to Gram-positive and Gram-negative strains decreased or remained unchanged compared to the original molecule **3**. Compound **19** (MIC = 25 µg/mL) was active in relation to the yeast-like fungus *Candida albicans.* It showed two times more activity compared to the original molecule **3** (MIC = 50 µg/mL).

When considering the relationship between “structure” and “biological activity” in relation to cytotoxicity, the following regularity was observed. For example, of the compounds shown in Scheme 1, the cytotoxicity of phenyltriazole derivative **3** (LD_50_ = 82.5 µg/mL) and chlorine-containing triazole derivative **6** (LD_50_ = 82.8 µg/mL) was slightly higher compared to that of the initial betulonic acid **2** (LD_50_ = 88.7 µg/mL), and the rest of the compounds, especially **4**, **8** and **9** (LD_50_ = 95.6–107.2 µg/mL), showed a decrease in activity compared to the original molecule **2**. It was observed that the cytotoxicity of the triazole derivative with methoxy group **5** was lost compared to the initial compound **2** (LD_50_ = 88.7 µg/mL).

When comparing the cytotoxicity of compounds **13**–**16** (LD_50_ = 85.1–105.9 µg/mL) with that of the starting compounds **3**, **5**, **7** and **8** (LD_50_ = 82.5–107.2 µg/mL), it was found that the cytotoxicity of compounds **14** and **16** (LD_50_ = 90.5 and 85.1 µg/mL) increased. 

In addition, compounds **18**–**20** and **22** (LD_50_ = 63.5–74.1 µg/mL) were found to be cytotoxic compared to the original molecule **3** (LD_50_ = 82.5 µg/mL). And in compounds **17** and **21**, on the contrary, activity was lost.

So, considering the relationship between “structure” and “biological activity”, the following conclusion can be made in relation to these synthesized derivatives. By introducing a triazole cycle with various substituents into a molecule, substances with antibacterial action can be obtained. In particular, halogen-containing triazole derivatives, i.e., chlorine and fluorine atoms, showed very high antimicrobial activity. It turned out that the presence of a fluorine atom and an ethanolamine fragment in the molecules of derivatives obtained with further conversion of triazole derivatives led to a doubling of activity against microbes and fungi. In addition, it was found that the synthesized derivatives had high antimicrobial activity due to the presence of phenyl, methyl and methoxy groups in the molecules. The introduction of the triazole cycle and substituted fragments into the synthesized compounds did not lead to a significant change in cytotoxicity compared with the initial molecules. However, in some compounds containing cyclopropane, ethylhydroxy and propylhydroxy fragments introduced into the molecules increased the cytotoxicity compared to that of the starting triazole derivatives.

## 3. Materials and Methods

### 3.1. General Chemistry Section

The ^1^H and ^13^C spectra of the compounds were measured on a Bruker (Billerica, MA, USA) AMX 400 MHz instrument. Tetramethylsilane and an NMR solvent were used in some spectra as internal standards. The melting points were determined using the Reichert (Carrollton, TX, USA) Thermovar. For column chromatography, 70−230 mesh silica 60 (E.M. Merck, Rahway, NJ, USA) was used as the stationary phase. Dichlorometane, petroleum ether and ethylacetate were used as eluents. The TLC (thin-layer chromatography) results were checked on silica gel 0.20 mm 60 with a mixture of ethanol and sulfuric acid (10 mL of sulfuric acid and 90 mL of ethanol), and UV was also used. Chemicals from commercial sources were used without further purification. Reaction dry solvents (toluene, THF, DMF and CH_2_Cl_2_) from commercial sources were used. UHPLC-ESI-Q-TOF-MS analyses of small molecules were performed using a Dionex (Sunnyvale, CA, USA) Ultimate (Arlington, VA, USA) 3000 UHPLC coupled to a Zorbax (Santa Clara, CA, USA) RRHP Eclipse Plus C18 column (2.1 × 100 mm, 1.8 µL) connected to a Bruker Impact II QTOF mass spectrometer. Mobile phases consisted of water (A) and acetonitrile (B), each supplemented with 0.1% formic acid. The following gradient was used at a flow rate of 0.200 mL/min: 0–2.5 min 5% B, 2.5–14 min 5–100% B, 14–19 min 100% B, 19–20.4 min 100–5% B, 20.4–25 min 5% B. The mass spectrometer was operated in positive-ion mode with a scan range of 50–3000 *m/z*. The source conditions were as follows: end plate offset at −500 V; capillary at −4500 V; nebulizer gas (N_2_) at 1.6 bar; dry gas (N_2_) at 8 L min^−1^; dry temperature at 180 °C. The ion transfer conditions were as follows: ion funnel RF at 200 Vpp; multiple RF at 200 Vpp; quadrupole low mass at 55 *m/z*; collision energy at 5.0 eV; collision RF at 600 Vpp; ion cooler RF at 50–350 Vpp; transfer time at 121 μs; pre-pulse storage time of 1 μs. Calibration was performed with 1 mM sodium formate through a loop injection of 20 μL at the start of each run. Also, mass spectra were acquired on a quadrupole orthogonal acceleration time-of-flight mass spectrometer (Synapt G2 HDMS, Waters, Milford, MA, USA). Samples were infused at 3 µL/min, and spectra were obtained in positive (or negative) ionization mode with a resolution of 15,000 (FWHM) using leucine enkephalin as the lock mass. 

Betulonic acid **2** was prepared as previously reported [51]. The spectral data corresponded to the data reported in the literature [52,53]. The ^1^H, ^13^C NMR and mass spectra of compounds **2**–**22** can be found in the Supplementary Materials.

### 3.2. Experimental Section

*3-Oxo-lup-20(29)-en-28-oic Acid* (**Betulonic Acid, 2**). Betulin (5.0 g, 11.3 mmol) was placed in a 0.5 l flask filled with acetone (150 mL) and dissolved in an ultrasonic water bath. Freshly prepared Jones reagent (6.65 g Na_2_Cr_2_O_7_ and 6 mL H_2_SO_4_ in 50 mL water) was added drop by drop to the cooled (on an ice bath) solution. The color of the solution began to change. The reaction mixture was allowed to heat up to room temperature and continued to be stirred for 5 h. The course of the reaction was checked by TLC. Then, MeOH (100 mL) was added to the reaction mixture, followed by water (50 mL). The resulting precipitate was filtered and washed with water (50 mL). The crude product was dried, then dissolved in Et_2_O (60 mL) and washed with water (30 mL), 7.5% hydrochloric acid (20 mL), water (20 mL), saturated aqueous solution NaHCO_3_ (20 mL) and water (20 mL). The ether layer was driven off on a rotary evaporator, and the remaining residue was purified by column chromatography (silica gel); a mixture of heptane and ethyl acetate (80:20) was used as an eluent. Compound **2** was a colorless powder. The product yield was 1.55 g (30%).

*^1^H NMR (400 MHz, CDCl_3_)*: δ 4.74 (d, *J* = 2.3 Hz, 1H), 4.66–4.58 (m, 1H), 3.01 (td, *J* = 10.7, 4.6 Hz, 1H), 2.56–2.35 (m, 2H), 2.33–2.17 (m, 2H), 2.07–1.83 (m, 2H), 1.70 (s, 4H), 1.64 (t, *J* = 11.4 Hz, 1H), 1.59–1.14 (m, 14H), 1.13–1.04 (m, 4H), 1.03–0.95 (m, 9H), 0.93 (s, 3H).

*^13^C NMR (101 MHz, CDCl_3_)*: δ 218.23, 181.72, 150.32, 109.79, 56.37, 54.94, 49.85, 49.19, 47.34, 46.89, 42.49, 40.64, 39.61, 38.51, 37.04, 36.92, 34.13, 33.60, 32.10, 30.55, 29.68, 26.64, 25.49, 21.37, 21.00, 19.63, 19.37, 15.96, 15.82, 14.63.

*1′-((S)-1-Phenylethyl)-1H′-lup-2-eno-[2,3-d]-[1,2,3]-triazole-28 oic Acid* (**3**). Betulonic acid (1.0 g, 1 equiv., 2.2 mmol), 4-nitrophenyl azide (360 mg, 1.3 equiv., 2.86 mmol), (S)-(–)-α-methylbenzylamine (350 mg, 1.3 equiv., 2.86 mmol) and 4 Å molecular sieves (200 mg) were added to a dried reaction tube with a screw cap equipped with a magnetic stirrer. The mixture was dissolved in dry toluene (5 mL), and the reaction mixture was stirred at 100 °C for 24 h. A mixture of H_2_SO_4_ and ethanol (10:90) was used to visualize the TLC plates. The crude reaction mixture was purified directly using column chromatography (silica gel), first using dichloromethane to remove all 4-nitroaniline formed during the reaction, and then a mixture of petroleum ether and ethyl acetate was used as an eluent. Compound **3** was powdery with a yellowish tinge, m.p. 325–327 °C. The yield was 790 mg (61%). The spectroscopic data for compound **3** were consistent with previously reported data for this compound [41].

*^1^H NMR (400 MHz, CDCl_3_)*: δ 7.33–7.25 (m, 3H), 7.25–7.19 (m, 2H), 5.72 (q, *J* = 7.0 Hz, 1H), 4.77 (d, *J* = 2.3 Hz, 1H), 4.64 (m, 1H), 3.03 (td, *J* = 10.6, 4.5 Hz, 1H), 2.96 (d, *J* = 15.3 Hz, 1H), 2.32–2.20 (m, 2H), 2.15 (d, *J* = 15.3 Hz, 1H), 2.05–1.95 (m, 5H), 1.81–1.60 (m, 5H), 1.61–1.32 (m, 11H), 1.26 (s, 5H), 1.10 (s, 3H), 0.99 (s, 6H), 0.81 (s, 3H).

*^13^C NMR (101 MHz, CDCl_3_)*: δ 181.07, 150.22, 141.79, 141.05, 137.53, 128.64, 127.56, 126.24, 109.88, 59.28, 56.38, 54.78, 49.34, 49.21, 46.90, 42.45, 40.60, 38.91, 38.53, 38.32, 37.04, 33.80, 33.41, 32.08, 30.59, 29.80, 28.89, 25.50, 23.73, 21.36, 21.31, 19.42, 18.91, 16.25, 15.70, 14.65.

*1′-(3-Methylbenzyl)-1H′-lup-2-eno-[2,3-d]-[1,2,3]-triazole-28-oic Acid* (**4**). Betulonic acid **2** (100 mg, 1 equiv., 0.22 mmol), 4-nitrophenyl azide (72 mg, 2 equiv., 0.44 mmol), 3-methylbenzylamine (74.6 mg, 2.8 equiv., 0.616 mmol) and 4 Å molecular sieves (50 mg) were added to a dried reaction tube with a screw cap equipped with a magnetic stirrer. The mixture was dissolved in dry toluene (1 mL), and the reaction mixture was stirred at 100 °C for 24 h. A mixture of H_2_SO_4_ and ethanol (10:90) was used to visualize the TLC plates. The crude reaction mixture was purified directly using column chromatography (silica gel), first using dichloromethane to remove all 4-nitroaniline formed during the reaction, and then a mixture of petroleum ether and ethyl acetate (10:1) was used as an eluent. Compound **4** was a pale-yellow powder, m.p. 261–264 °C. The yield was 72 mg (68%).

*^1^H NMR (400 MHz, CDCl_3_)*: δ 7.17 (t, *J* = 7.6 Hz, 1H), 7.06 (d, *J* = 7.6 Hz, 1H), 6.85 (s, 1H), 6.81 (d, *J* = 7.7 Hz, 1H), 5.62 (m, 2H), 4.76 (d, *J* = 2.3 Hz, 1H), 4.64 (m, 1H), 4.12 (q, *J* = 7.2 Hz, OH), 3.04 (td, *J* = 10.6, 4.5 Hz, 1H), 2.96 (d, *J* = 15.3 Hz, 1H), 2.25 (m, 6H), 1.99 (m, 2H), 1.77 (d, *J* = 12.8 Hz, 1H), 1.71 (s, 3H), 1.45 (m, 13H), 1.17 (s, 3H), 1.04 (s, 3H), 0.99 (d, *J* = 9.2 Hz, 6H), 0.90 (m, 1H), 0.78 (s, 3H).

*^13^C NMR (101 MHz, CDCl_3_)*: δ 181.38, 150.25, 141.84, 138.49, 138.00, 136.38, 128.59, 128.52, 127.03, 123.46, 109.86, 56.40, 54.59, 52.81, 49.29, 49.19, 46.90, 42.44, 40.56, 38.97, 38.51, 38.36, 37.04, 33.71, 33.35, 32.08, 30.58, 29.79, 28.77, 25.50, 21.41, 21.37, 21.31, 19.42, 18.91, 16.08, 15.70, 14.65.

*HRMS (ESI+)*: *m/z* calculated for C_38_H_54_N_3_O_2_ [M + H]^+^: 584.42160, found: 584.4216.

*1′-(3-Methoxybenzyl)-1H′-lup-2-eno-[2,3-d]-[1,2,3]-triazole-28-oic Acid* (**5**). Betulonic acid (100 mg, 1 equiv., 0.22 mmol), 4-nitrophenyl azide (72 mg, 2 equiv., 0.44 mmol), 3-methoxybenzylamine (84.5 mg, 2.8 equiv., 0.616 mmol) and 4 Å molecular sieves (50 mg) were added to a dried reaction tube with a screw cap equipped with a magnetic stirrer. The mixture was dissolved in dry toluene (1 mL), and the reaction mixture was stirred at 100 °C for 18 h. A mixture of H_2_SO_4_ and ethanol (10:90) was used to visualize the TLC plates. The crude reaction mixture was purified directly using column chromatography (silica gel), first using dichloromethane to remove all 4-nitroaniline formed during the reaction, and then a mixture of petroleum ether and ethyl acetate was used as an eluent. Compound **5** was powdery with a yellowish tinge, m.p. 205–208 °C. The yield was 78 mg (60%).

*^1^H NMR (400 MHz, CDCl_3_)*: δ 7.21 (td, *J* = 7.9, 3.3 Hz, 1H), 6.83–6.75 (m, 1H), 6.65–6.54 (m, 3H), 5.62 (d, *J* = 3.8 Hz, 3H), 4.76 (d, *J* = 2.3 Hz, 1H), 4.67–4.63 (m, 1H), 3.74 (d, *J* = 1.6 Hz, 4H), 3.09–2.92 (m, 2H), 2.26 (ddd, *J* = 15.5, 9.7, 3.5 Hz, 2H), 2.21–2.14 (m, 1H), 2.06–1.82 (m, 3H), 1.81–1.75 (m, 2H), 1.71 (s, 3H), 1.68 (s, 0H), 1.61–1.36 (m, 10H), 1.35–1.18 (m, 4H), 1.17 (d, *J* = 2.6 Hz, 3H), 1.04 (s, 3H), 1.02–0.96 (m, 8H), 0.81 (s, 1H), 0.77 (s, 3H).

*^13^C NMR (101 MHz, CDCl_3_)*: δ 180.81, 159.97, 150.24, 141.89, 138.09, 138.04, 129.78, 118.65, 113.31, 111.96, 109.87, 56.36, 55.23, 54.79, 54.59, 52.74, 52.66, 49.30, 49.19, 46.88, 44.09, 42.45, 40.56, 40.36, 38.98, 38.48, 38.37, 37.03, 33.72, 33.35, 32.07, 30.57, 29.79, 28.73, 25.50, 21.38, 21.31, 19.42, 18.91, 16.08, 15.71, 15.19, 14.65.

*HRMS (ESI+)*: *m/z* calculated for C_38_H_54_N_3_O_3_ [M + H]^+^: 600.4160, found: 600.4194.

*1′-(3-Chlorobenzyl)-1H′-lup-2-eno-[2,3-d]-[1,2,3]-triazole-28-oic Acid* (**6**). Betulonic acid **2** (100 mg, 1 equiv., 0.22 mmol), 4-nitrophenyl azide (72 mg, 2 equiv., 0.44 mmol), 3-chlorobenzylamine (87 mg, 2.8 equiv., 0.616 mmol) and 4 Å molecular sieves (50 mg) were added to a dried reaction tube with a screw cap equipped with a magnetic stirrer. The mixture was dissolved in dry toluene (1 mL), and the reaction mixture was stirred at 100 °C for 24 h. A mixture of H_2_SO_4_ and ethanol (10:90) was used to visualize the TLC plates. The crude reaction mixture was purified directly using column chromatography (silica gel), first using dichloromethane to remove all 4-nitroaniline formed during the reaction, and then a mixture of petroleum ether and ethyl acetate (1:1) was used as an eluent. Compound **6** was a pale-yellow powder, m.p. 288–290 °C. The yield was 65 mg (62%).

*^1^H NMR (400 MHz, CDCl_3_)*: δ 7.28–7.19 (m, 2H), 7.03 (d, *J* = 2.0 Hz, 1H), 6.89 (dt, *J* = 5.7, 2.1 Hz, 1H), 5.61 (d, *J* = 5.5 Hz, 2H), 4.76 (d, *J* = 2.3 Hz, 1H), 4.64 (t, *J* = 1.9 Hz, 1H), 3.04 (td, *J* = 10.6, 4.6 Hz, 1H), 2.96 (d, *J* = 15.4 Hz, 1H), 2.28 (ddd, *J* = 14.7, 9.0, 3.2 Hz, 2H), 2.23–2.15 (m, 1H), 2.00 (ddd, *J* = 17.8, 11.6, 5.5 Hz, 2H), 1.83–1.73 (m, 1H), 1.71 (s, 3H), 1.67–1.19 (m, 13H), 1.17 (s, 4H), 1.06–0.95 (m, 9H), 0.78 (s, 3H).

*^13^C NMR (101 MHz, CDCl_3_)*: δ 181.63, 150.23, 142.05, 130.06, 128.07, 126.53, 124.55, 109.87, 56.41, 54.51, 52.16, 49.27, 49.20, 46.91, 42.44, 40.55, 38.98, 38.53, 38.29, 37.03, 33.69, 33.31, 32.07, 30.58, 29.80, 28.83, 25.47, 21.41, 21.37, 19.42, 18.88, 16.09, 15.73, 14.64.

*HRMS (ESI+)*: *m/z* calculated for C_37_H_51_ClN_3_O_2_ [M + H]^+^: 604.36698, found: 604.3661.

*1′-(3-Fluorobenzyl)-1H′-lup-2-eno-[2,3-d]-[1,2,3]-triazole-28-oic Acid* (**7**). Betulonic acid (100 mg, 1 equiv., 0.22 mmol), 4-nitrophenyl azide (72 mg, 2 equiv., 0.44 mmol), 3-fluorobenzylamine (77 mg, 2.8 equiv., 0.616 mmol) and 4 Å molecular sieves (50 mg) were added to a dried reaction tube with a screw cap equipped with a magnetic stirrer. The mixture was dissolved in dry toluene (1 mL), and the reaction mixture was stirred at 100 °C for 22 h. A mixture of H_2_SO_4_ and ethanol (10:90) was used to visualize the TLC plates. The crude reaction mixture was purified directly using column chromatography (silica gel), first using dichloromethane to remove all 4-nitroaniline formed during the reaction, and then a mixture of petroleum ether and ethyl acetate was used as an eluent. Compound **7** was powdery with a yellowish tinge, m.p. 302–305 °C. The yield was 82 mg (64%).

*^1^H NMR (400 MHz, CDCl_3_)*: δ 7.27 (d, *J* = 5.5 Hz, 2H), 7.00–6.92 (m, 1H), 6.81 (ddd, *J*_HF_ = 7.8 Hz, *J*_HH_ =1.8, 0.9 Hz, 1H), 6.71 (dt, *J*_HF_ = 9.6 Hz, *J*_HH_ = 2.2 Hz, 1H), 5.68–5.58 (m, 2H), 4.76 (d, *J* = 2.3 Hz, 1H), 4.67–4.62 (m, 1H), 3.03 (td, *J* = 10.6, 4.6 Hz, 1H), 2.96 (d, *J* = 15.4 Hz, 1H), 2.27 (td, *J* = 12.7, 11.3, 3.3 Hz, 2H), 2.23–2.15 (m, 1H), 2.07–1.93 (m, 2H), 1.77 (dd, *J* = 11.4, 4.1 Hz, 1H), 1.71 (s, 3H), 1.66 (t, *J* = 11.4 Hz, 1H), 1.62–1.33 (m, 9H), 1.33–1.18 (m, 3H), 1.17 (s, 3H), 1.04 (s, 3H), 0.99 (d, *J* = 8.9 Hz, 6H), 0.78 (s, 3H).

*^13^C NMR (101 MHz, CDCl_3_)*: δ 181.18, 161.82 (d, ^1^*J*_C-F_ *=* 245.5 Hz), 150.22, 142.05, 139.03 (d, ^3^*J*_C-F_ *=* 7.2 Hz), 138.10, 130.36 (d, ^3^*J*_C-F_ *=* 8.2 Hz), 121.99 (d, ^4^*J*_C-F_ *=* 2.9 Hz), 114.80 (d, ^2^*J*_C-F_ *=* 22.5 Hz), 113.50 (d, ^2^*J*_C-F_ *=* 21.1 Hz), 109.88, 56.38, 54.53, 52.24, 49.28, 49.20, 46.91, 42.45, 40.56, 38.98, 38.53, 38.31, 37.02, 33.69, 33.32, 32.07, 30.58, 29.80, 28.78, 25.48, 21.37, 19.42, 18.89, 16.10, 15.73, 14.65.

*HRMS (ESI+)*: *m/z* calculated for C_37_H_51_FN_3_O_2_ [M + H]^+^: 588.3960, found: 588.3966.

*1′-Butyl-1H′-lup-2-eno-[2,3-d]-[1,2,3]-triazole-28-oic Acid* (**8**). Betulonic acid (100 mg, 1 equiv., 0.22 mmol), 4-nitrophenyl azide (36 mg, 1 equiv., 0.22 mmol), n-butylamine (21 mg, 1.3 equiv., 0.286 mmol) and 4 Å molecular sieves (50 mg) were added to a dried reaction tube with a screw cap equipped with a magnetic stirrer. The mixture was dissolved in dry toluene (1 mL), and the reaction mixture was stirred at 100 °C for 24 h. A mixture of H_2_SO_4_ and ethanol (10:90) was used to visualize the TLC plates. The crude reaction mixture was purified directly using column chromatography (silica gel), first using dichloromethane to remove all 4-nitroaniline formed during the reaction, and then a mixture of petroleum ether and ethyl acetate was used as an eluent. Compound **8** was powdery with a yellowish tinge, m.p. 288–290 °C. The yield was 74 mg (63%).

*^1^H NMR (400 MHz, CDCl_3_)*: δ 4.76 (d, *J* = 2.3 Hz, 1H), 4.67–4.61 (m, 1H), 4.29 (td, *J* = 7.1, 1.6 Hz, 2H), 3.04 (td, *J* = 10.5, 4.5 Hz, 1H), 2.91 (d, *J* = 15.3 Hz, 1H), 2.34–2.20 (m, 2H), 2.13 (d, *J* = 15.4 Hz, 1H), 2.06–1.93 (m, 4H), 1.80–1.73 (m, 1H), 1.71 (s, 3H), 1.65 (d, *J* = 11.5 Hz, 1H), 1.62–1.33 (m, 11H), 1.30 (s, 3H), 1.26 (s, 4H), 1.18 (s, 3H), 1.04–0.93 (m, 10H), 0.93–0.80 (m, 1H), 0.77 (s, 3H).

*^13^C NMR (101 MHz, CDCl_3_)*: δ 181.21, 150.24, 141.03, 109.86, 56.39, 54.68, 49.37, 49.30, 49.21, 46.90, 42.46, 40.59, 38.98, 38.54, 38.27, 37.05, 33.66, 33.39, 32.85, 32.09, 30.60, 29.81, 29.71, 28.68, 25.50, 21.33, 20.18, 19.43, 18.98, 16.06, 15.72, 14.69, 13.70.

*HRMS (ESI+)*: *m/z* calculated for C_34_H_54_N_3_O_2_ [M + H]^+^: 536.4211, found: 536.4214.

*1′-Isobutyl-1H′-lup-2-eno-[2,3-d]-[1,2,3]-triazole-28-oic Acid* (**9**). Betulonic acid **2** (100 mg, 1 equiv., 0.22 mmol), 4-nitrophenyl azide (36 mg, 1 equiv., 0.22 mmol), isobutylamine (21 mg, 1.3 equiv., 0.286 mmol) and 4 Å molecular sieves (50 mg) were added to a dried reaction tube with a screw cap equipped with a magnetic stirrer. The mixture was dissolved in dry toluene (1 mL), and the reaction mixture was stirred at 100 °C for 24 h. A mixture of H_2_SO_4_ and ethanol (10:90) was used to visualize the TLC plates. The crude reaction mixture was purified directly using column chromatography (silica gel), first using dichloromethane to remove all 4-nitroaniline formed during the reaction, and then a mixture of petroleum ether and ethyl acetate (10:1) was used as an eluent. Compound **9** was a pale-yellow powder, m.p. 299–301 °C. The yield was 68 mg (65%).

*^1^H NMR (400 MHz, CDCl_3_)*: δ 4.76 (d, *J* = 2.3 Hz, 1H), 4.64 (t, *J* = 1.9 Hz, 1H), 4.16–4.03 (m, 2H), 3.04 (td, *J* = 10.6, 4.6 Hz, 1H), 2.92 (d, *J* = 15.3 Hz, 1H), 2.54 (dq, *J* = 13.8, 6.9 Hz, 1H), 2.35–2.19 (m, 2H), 2.14 (d, *J* = 15.3 Hz, 1H), 2.08–1.94 (m, 2H), 1.81–1.73 (m, 1H), 1.71 (s, 3H), 1.69–1.32 (m, 10H), 1.30 (s, 3H), 1.24 (d, *J* = 16.4 Hz, 3H), 1.17 (s, 3H), 1.15–1.03 (m, 1H), 1.03–0.93 (m, 13H), 0.93–0.83 (m, 1H), 0.77 (s, 3H).

*^13^C NMR (101 MHz, CDCl_3_)*: δ 181.59, 150.26, 140.94, 137.79, 109.83, 56.56, 56.42, 54.74, 49.30, 49.22, 46.91, 42.45, 40.58, 38.90, 38.55, 38.28, 37.05, 33.73, 33.38, 32.10, 30.61, 29.82, 29.38, 28.90, 25.50, 21.57, 21.36, 20.25, 20.21, 19.43, 19.00, 16.04, 15.74, 14.68.

*HRMS (ESI+)*: *m/z* calculated for C_34_H_54_N_3_O_2_ [M + H]^+^: 536.42160, found: 536.4209.

*1H′-Lup-2-eno-[2,3-d]-[1,2,3]-triazole-28-oic Acid* (**10**). Betulonic acid **2** (100 mg, 1 equiv., 0.22 mmol), ammonium acetate (84.8 mg, 5 equiv., 1.1 mmol) and 4-nitrophenyl azide (45.8 mg, 1.3 equiv., 0.28 mmol) were dissolved in dry DMF (1 mL). The reaction mixture was stirred at 80 °C for 24 h. A mixture of H_2_SO_4_ and ethanol (10:90) was used to visualize the TLC plates. The crude reaction mixture was purified directly using column chromatography (silica gel), first using dichloromethane to remove all 4-nitroaniline formed during the reaction, and then a mixture of petroleum ether and ethyl acetate (10:1) was used as an eluent. Compound **10** was a pale-yellow powder, m.p. 158 °C. The yield was 48 mg (46%). The spectroscopic data for compound **10** were consistent with previously reported data for this compound [41].

*^1^H NMR (400 MHz, CDCl_3_)*: δ 4.77 (d, *J* = 2.5 Hz, 1H), 4.64 (d, *J* = 1.9 Hz, 1H), 3.05 (td, *J* = 10.7, 4.6 Hz, 1H), 2.90 (d, *J* = 15.5 Hz, 1H), 2.36–2.22 (m, 2H), 2.19–2.09 (m, 1H), 2.08–1.93 (m, 2H), 1.83–1.74 (m, 1H), 1.72 (s, 3H), 1.68–1.36 (m, 15H), 1.32 (s, 4H), 1.30–1.18 (m, 4H), 1.18–1.04 (m, 1H), 1.01 (d, *J* = 10.2 Hz, 6H), 0.97–0.80 (m, 6H), 0.77 (s, 3H).

*^13^C NMR (101 MHz, CDCl_3_)*: δ 181.38, 150.35, 149.92, 140.40, 109.80, 56.40, 53.42, 49.22, 49.09, 46.95, 42.51, 41.35, 40.75, 39.04, 38.51, 37.32, 37.07, 33.72, 33.38, 33.30, 32.16, 31.01, 30.62, 29.80, 29.06, 27.67, 25.51, 23.76, 22.63, 21.41, 20.45, 19.42, 19.16, 16.27, 15.69, 14.68.

Compound (**11**) and (**12**). Compound **10** (181 mg) was dissolved in dry methanol (1.5 mL), then potassium tret-butoxide (*t*ButOK) (100.08 mg, 0.91 mmol) was added. The reaction mixture was stirred at room temperature for 3 h, after which 1-bromobutane was slowly added and stirred at 60 °C for 12 h. The solvent was distilled in a rotary evaporator. The crude reaction mixture was purified directly using column chromatography (silica gel), using a mixture of petroleum ether and ethyl acetate as an eluent. Compound **11** was a colorless substance. The yield was 103 mg (46%).

*^1^H NMR (400 MHz, CDCl_3_)*: δ 4.76 (d, *J* = 2.3 Hz, 1H), 4.63 (t, *J* = 1.8 Hz, 1H), 4.32 (t, *J* = 7.3 Hz, 2H), 4.09 (qt, *J* = 10.8, 6.6 Hz, 2H), 3.04 (td, *J* = 10.8, 4.5 Hz, 1H), 2.83 (d, *J* = 15.4 Hz, 1H), 2.29 (td, *J* = 12.4, 11.7, 3.6 Hz, 2H), 2.07 (d, *J* = 15.4 Hz, 1H), 1.96–1.85 (m, 4H), 1.80–1.72 (m, 1H), 1.71 (s, 3H), 1.68–1.30 (m, 15H), 1.29 (d, *J* = 4.0 Hz, 4H), 1.21 (s, 2H), 1.17 (d, *J* = 5.7 Hz, 4H), 1.15–1.02 (m, 1H), 1.00 (s, 3H), 0.98 (d, *J* = 4.3 Hz, 4H), 0.96–0.81 (m, 11H), 0.79 (s, 3H).

*^13^C NMR (101 MHz, CDCl_3_)*: δ 176.22, 150.89, 150.54, 141.49, 109.63, 63.72, 56.58, 54.41, 53.50, 49.35, 49.17, 47.01, 42.46, 40.76, 38.90, 38.35, 37.62, 37.05, 33.50, 33.39, 32.14, 32.05, 31.08, 30.81, 30.68, 29.72, 25.61, 23.82, 21.44, 19.84, 19.44, 19.32, 19.21, 16.26, 15.63, 14.67, 13.72, 13.59.

*HRMS (ESI+)*: *m/z* calculated for C_38_H_62_N_3_O_2_ [M + H]^+^: 592.48420, found: 592.4850.

Compound **12** was a colorless substance. The yield was 45 mg (20%).

*^1^H NMR (400 MHz, CDCl_3_)* δ 4.75 (d, *J* = 2.4 Hz, 1H), 4.61 (m, 1H), 4.17 (m, 1H), 4.10 (m, 3H), 3.05 (td, *J* = 10.8, 4.4 Hz, 1H), 2.61 (d, *J* = 15.3 Hz, 1H), 2.37–2.24 (m, 2H), 2.05–1.98 (m, 1H), 1.97–1.85 (m, 2H), 1.84–1.73 (m, 3H), 1.70 (s, 3H), 1.68–1.37 (m, 13H), 1.33 (d, *J* = 10.5 Hz, 4H), 1.27–1.20 (m, 6H), 1.18–1.03 (m, 2H), 1.01 (s, 3H), 0.98 (d, *J* = 5.2 Hz, 3H), 0.95 (d, *J* = 6.8 Hz, 4H), 0.93–0.82 (m, 5H), 0.79 (s, 3H).

*^13^C NMR (101 MHz, CDCl_3_)* δ 176.17, 150.68, 150.15, 129.16, 109.61, 63.76, 56.52, 53.37, 49.34, 49.27, 47.36, 47.00, 42.49, 40.87, 39.24, 38.31, 37.03, 36.06, 33.65, 33.36, 32.16, 32.09, 30.81, 30.70, 30.59, 29.70, 25.58, 23.08, 21.59, 19.86, 19.38, 19.31, 19.00, 16.63, 15.63, 14.65, 13.72, 13.60.

*HRMS (ESI+)*: *m/z* calculated for C_38_H_62_N_3_O_2_ [M + H]^+^: 592.48420, found: 592.4844.

*1′-((S)-1-Phenylethyl)-1H′-lup-2-eno-[2,3-d]-[1,2,3]-triazole-28-(1H-triazol-1-yl)* (**13**). Compound **3** (100 mg, 1 equiv., 0.171 mmol) was dissolved in 2 mL of THF, and 1,1′-carbonyldi-(1,2,4-triazole) (112.1 mg, 4 equiv., 0.684 mmol) was added. The reaction mixture was stirred at 70 °C. The reaction was carried out for 5 h. The solvent was distilled in a rotary evaporator. The remainder was chromatographed with silica gel in a column. When the column was eluted with a mixture of petroleum ether and ethyl acetate (5:3), compound **13** was isolated. Compound **13** was a colorless powder, m.p. 232–235 °C. The yield was 116 mg (107%).

*^1^H NMR (400 MHz, CDCl_3_)*: δ 8.93 (s, 1H), 7.99 (s, 1H), 7.34–7.26 (m, 3H), 7.24–7.18 (m, 3H), 5.72 (q, *J* = 7.0 Hz, 1H), 4.79 (d, *J* = 2.1 Hz, 1H), 4.68 (t, *J* = 1.8 Hz, 1H), 3.04–2.90 (m, 3H), 2.74–2.55 (m, 2H), 2.20–2.09 (m, 2H), 2.02 (d, *J* = 7.0 Hz, 3H), 1.90–1.75 (m, 3H), 1.73 (s, 3H), 1.72–1.57 (m, 2H), 1.57–1.30 (m, 6H), 1.30–1.13 (m, 9H), 1.09 (s, 3H), 0.99 (d, *J* = 10.7 Hz, 6H), 0.95–0.86 (m, 1H), 0.85 (s, 3H).

*^13^C NMR (101 MHz, CDCl_3_)*: δ 152.19, 145.19, 141.02, 128.64, 127.55, 126.23, 110.20, 59.28, 58.45, 54.84, 50.92, 49.54, 45.65, 42.33, 40.62, 38.94, 38.41, 37.19, 36.26, 33.81, 33.38, 31.47, 30.54, 29.97, 28.89, 25.53, 23.74, 21.52, 21.32, 19.40, 18.90, 16.29, 15.67, 14.65.

*HRMS (ESI+)*: *m/z* calculated for C_40_H_55_N_6_O [M + H]^+^: 635.4432, found: 635.4393.

*1′-(3-Methoxybenzyl)-1H′-lup-2-eno-[2,3-d]-[1,2,3]-triazole-28-(1H-triazol-1-yl)* (**14**). Compound **5** (93 mg, 1 equiv., 0.155 mmol) was dissolved in 2 mL of THF, and 1,1′-carbonyldi-(1,2,4-triazole) (152.6 mg, 6 equiv., 0.93 mmol) was added. The reaction mixture was stirred at 70 °C. The reaction was carried out for 24 h. The solvent was distilled in a rotary evaporator. The remainder was chromatographed with silica gel in a column. When the column was eluted with a mixture of petroleum ether and ethyl acetate (4:1), compound **14** was isolated. Compound **14** was a colorless powder, m.p. 279–282 °C. The yield was 52 mg (52%).

*^1^H NMR (400 MHz, CDCl_3_)*: δ 8.92 (s, 1H), 7.99 (s, 1H), 7.21 (t, *J* = 7.9 Hz, 1H), 6.82–6.77 (m, 1H), 6.63–6.58 (m, 1H), 6.56 (t, *J* = 2.1 Hz, 1H), 5.62 (s, 2H), 4.80 (d, *J* = 2.2 Hz, 1H), 4.71–4.64 (m, 1H), 3.74 (s, 3H), 3.05–2.90 (m, 3H), 2.68 (td, *J* = 12.4, 3.4 Hz, 1H), 2.60 (dd, *J* = 12.9, 7.3 Hz, 1H), 2.17 (s, 5H), 1.92–1.75 (m, 3H), 1.74 (s, 3H), 1.72–1.64 (m, 1H), 1.63 (d, *J* = 1.9 Hz, 2H), 1.60–1.34 (m, 5H), 1.35–1.19 (m, 2H), 1.18 (s, 3H), 1.06 (s, 3H), 1.03 (s, 3H), 0.97 (s, 4H), 0.80 (s, 3H).

*^13^C NMR (101 MHz, CDCl_3_)*: δ 173.39, 159.97, 152.21, 149.83, 145.18, 141.87, 138.10, 138.00, 129.78, 118.65, 113.27, 112.00, 110.20, 58.44, 55.23, 54.66, 52.74, 50.91, 49.49, 45.65, 42.33, 40.60, 39.01, 38.48, 37.18, 36.27, 33.74, 33.32, 31.47, 30.54, 29.96, 28.73, 25.53, 21.54, 21.35, 19.41, 18.91, 16.13, 15.65, 14.66.

*HRMS (ESI+)*: *m/z* calculated for C_40_H_55_N_6_O_2_ [M + H]^+^: 651.4381, found: 651.4378.

*1′-(3-Fluorobenzyl)-1H′-lup-2-eno-[2,3-d]-[1,2,3]-triazole-28-(1H-triazol-1-yl)* (**15**). Compound **7** (50 mg, 1 equiv., 0.085 mmol) was dissolved in 2 mL of THF, and 1,1′-carbonyldi-(1,2,4-triazole) (55.7 mg, 4 equiv., 0.34 mmol) was added. The reaction mixture was stirred at 70 °C. The reaction was carried out for 2.5 h. The solvent was distilled in a rotary evaporator. The formed compound was washed three times with cold acetone. Compound **15** was a colorless powder, m.p. 307–310 °C. The yield was 61 mg (112%).

*^1^H NMR (400 MHz, CDCl_3_)*: δ 8.92 (s, 1H), 7.99 (s, 1H), 7.31–7.23 (m, 2H), 7.00–6.91 (m, 1H), 6.81 (dt, *J*_HF_ = 7.8 Hz, *J*_HH_ = 1.3 Hz, 1H), 6.72 (dt, *J*_HF_ = 9.6 Hz, *J*_HH_ =2.1 Hz, 1H), 5.64 (d, *J* = 3.0 Hz, 2H), 4.80 (d, *J* = 2.2 Hz, 1H), 4.68 (t, *J* = 1.8 Hz, 1H), 3.75 (tdd, *J* = 5.8, 2.5, 1.6 Hz, 1H), 3.04–2.90 (m, 3H), 2.68 (td, *J* = 12.4, 3.4 Hz, 1H), 2.64–2.55 (m, 1H), 2.20 (d, *J* = 15.4 Hz, 1H), 1.91–1.75 (m, 4H), 1.74 (s, 3H), 1.70–1.34 (m, 8H), 1.25 (ddd, *J* = 25.0, 8.5, 3.2 Hz, 3H), 1.17 (s, 4H), 1.07 (s, 3H), 1.03 (s, 3H), 0.97 (s, 3H), 0.81 (s, 3H).

*^13^C NMR (101 MHz, CDCl_3_): δ ^13^C NMR (101 MHz, CDCl_3_)*: δ 173.38, 161.83 (d, ^1^*J*_C-F_ *=* 245.5 Hz), 152.21, 149.82, 145.18, 142.04, 139.01 (d, ^3^*J*_C-F_ *=* 7.2 Hz), 138.07, 130.35 (d, ^3^*J*_C-F_ *=* 8.3 Hz), 121.94 (d, ^4^*J*_C-F_ *=* 2.9 Hz), 114.83 (d, ^2^*J*_C-F_ *=* 20.9 Hz), 113.52 (d, ^2^*J*_C-F_ *=* 22.6 Hz), 110.21, 67.98, 58.44, 54.61, 52.25, 50.91, 49.49, 45.65, 42.34, 40.60, 39.01, 38.42, 37.18, 36.27, 33.72, 33.30, 31.46, 30.53, 29.96, 28.79, 25.52, 21.54, 21.41, 19.40, 18.90, 16.16, 15.65, 14.66.

*HRMS (ESI+)*: *m/z* calculated for C_39_H_52_FN_6_O [M + H]^+^: 639.4181, found: 639.4180.

*1′-Butyl-1H′-lup-2-eno-[2,3-d]-[1,2,3]-triazole-28-(1H-triazol-1-yl)* (**16**). Compound **8** (50 mg, 1 equiv., 0.0934 mmol) was dissolved in 2 mL of THF, and 1,1′-carbonyldi-(1,2,4-triazole) (61.2 mg, 4 equiv., 0.3736 mmol) was added. The reaction mixture was stirred at 70 °C. The reaction was carried out for 2 h. The solvent was distilled in a rotary evaporator. The formed compound was washed three times with cold acetone. Compound **16** was a colorless powder, m.p. 281–283° C. The yield was 59 mg (108%).

*^1^H NMR (400 MHz, CDCl_3_)*: δ 8.93 (s, 1H), 8.00 (s, 1H), 7.27 (s, 1H), 4.79 (d, *J* = 2.2 Hz, 1H), 4.71–4.63 (m, 1H), 4.29 (td, *J* = 7.1, 1.4 Hz, 2H), 3.05–2.89 (m, 3H), 2.68 (td, *J* = 12.4, 3.4 Hz, 1H), 2.63–2.56 (m, 1H), 2.19–2.10 (m, 2H), 1.97 (pd, *J* = 7.1, 1.4 Hz, 2H), 1.89–1.75 (m, 3H), 1.71 (d, *J* = 17.7 Hz, 5H), 1.66–1.34 (m, 7H), 1.30 (s, 3H), 1.27–1.18 (m, 4H), 1.12 (td, *J* = 13.0, 4.3 Hz, 1H), 1.03 (s, 3H), 1.01–0.94 (m, 6H), 0.80 (s, 3H).

*^13^C NMR (101 MHz, CDCl_3_)*: δ 173.39, 152.21, 149.82, 145.19, 141.02, 137.26, 110.20, 58.45, 54.75, 50.92, 49.50, 49.36, 45.65, 42.34, 40.62, 39.01, 38.39, 37.19, 36.27, 33.68, 33.36, 32.86, 31.48, 30.54, 29.98, 28.68, 25.53, 21.52, 21.36, 20.18, 19.40, 18.97, 16.11, 15.67, 14.70, 13.70.

*HRMS (ESI+)*: *m/z* calculated for C_36_H_55_N_6_O [M + H]^+^: 587.4432, found: 587.4438.

*1′-((S)-1-Phenylethyl)-1H′-lup-2-eno-[2,3-d]-[1,2,3]-triazole-isocyanate* (**17**). Compound **3** (200 mg, 1 equiv., 0.34 mmol) was dissolved in toluene (6 mL) in an ultrasonic bath, then triethylamine (0.0464 mL, 1 equiv., 0.34 mmol) and diphenylphosphoryl azide (0.0772 mL, 1 equiv., 0.34 mmol) were added. The reaction was carried out at room temperature (21 °C) for 6 h. At the end of the reaction, the solvent was distilled in a rotary evaporator. The remainder was chromatographed with silica gel in a column. Compound **17** was a colorless powder, m.p. 197–200 °C. The yield was 120 mg (61%).

*^1^H NMR (400 MHz, CDCl_3_)*: δ 7.29 (m, 3H), 7.22 (m, 2H), 5.73 (q, *J* = 7.0 Hz, 1H), 4.77 (d, *J* = 2.1 Hz, 1H), 4.67 (m, 1H), 2.97 (d, *J* = 15.3 Hz, 1H), 2.56 (td, *J* = 10.9, 5.8 Hz, 1H), 2.13 (m, 2H), 2.02 (d, *J* = 7.0 Hz, 3H), 1.84 (m, 5H), 1.70 (d, *J* = 1.2 Hz, 3H), 1.52 (m, 8H), 1.29 (s, 3H), 1.20 (m, 2H), 1.11 (d, *J* = 1.8 Hz, 6H), 0.94 (s, 3H), 0.84 (m, 3H).

*^13^C NMR (101 MHz, CDCl_3_)*: δ 148.71, 141.83, 140.99, 137.51, 128.64, 127.55, 126.24, 121.63, 110.63, 71.59, 59.27, 54.80, 49.32, 49.18, 48.10, 42.06, 40.66, 39.27, 39.17, 38.91, 38.42, 33.81, 33.57, 33.46, 29.29, 28.91, 27.81, 24.91, 23.74, 21.36, 21.34, 19.52, 18.89, 16.29, 15.69, 14.44.

*HRMS (ESI+)*: *m/z* calculated for C_38_H_53_N_4_O [M + H]^+^: 581.4214, found: 581.4218.

*1′-((S)-1-Phenylethyl)-1H′-lup-2-eno-[2,3-d]-[1,2,3]-triazole-28-oic Acid derivative-1.* (**18**). Compound **3** (100 mg, 1 equiv., 0.17 mmol) was dissolved in toluene (3 mL) in an ultrasonic bath, then triethylamine (0.024 mL, 1 equiv., 0.17 mmol) and diphenylphosphoryl azide (0.039 mL, 1 equiv., 0.17 mmol) were added. The reaction was carried out at room temperature (20 °C) for 7 h, after which cyclopropylamine (0.177 mL, 10 equiv., 1.7 mmol) in 0.5 mL of toluene was added to the reaction mixture. Then, it was heated at 110 °C for 4 h. At the end of the reaction, the solvent was distilled in a rotary evaporator. The remainder was chromatographed with silica gel in a column. During elution of the column with ethyl acetate, a colorless substance (compound **18**) with a melting point of 200–203 °C was isolated. The yield was 66 mg (61%).

*^1^H NMR (400 MHz, CDCl_3_)*: δ 7.34–7.25 (m, 3H), 7.23–7.18 (m, 2H), 5.73 (q, *J* = 7.0 Hz, 1H), 4.95 (s, 1H), 4.75 (d, *J* = 2.1 Hz, 1H), 4.69–4.60 (m, 2H), 2.96 (d, *J* = 15.2 Hz, 1H), 2.67 (dt, *J* = 13.2, 3.3 Hz, 1H), 2.54 (dd, *J* = 12.5, 8.1 Hz, 1H), 2.44 (tq, *J* = 7.9, 4.6, 4.1 Hz, 2H), 2.16 (d, *J* = 15.2 Hz, 1H), 2.07–1.91 (m, 4H), 1.82–1.64 (m, 7H), 1.62–1.16 (m, 11H), 1.10 (d, *J* = 9.6 Hz, 6H), 0.99 (s, 3H), 0.95–0.75 (m, 6H), 0.69–0.56 (m, 2H).

*^13^C NMR (101 MHz, CDCl_3_)*: δ 158.04, 149.40, 141.76, 140.94, 137.53, 128.66, 127.59, 126.23, 110.27, 63.72, 59.28, 54.74, 49.31, 49.23, 48.33, 42.07, 40.64, 38.89, 38.42, 38.37, 35.19, 33.83, 33.15, 29.86, 29.33, 28.89, 27.34, 25.11, 23.72, 22.70, 21.48, 21.35, 19.28, 18.90, 16.29, 15.58, 14.48, 7.82, 7.35.

*HRMS (ESI+)*: *m/z* calculated for C_41_H_60_N_5_O [M + H]^+^: 638.4792, found: 638.4750.

*1′-((S)-1-Phenylethyl)-1H′-lup-2-eno-[2,3-d]-[1,2,3]-triazole-28-oic Acid derivative-2.* (**19**). Compound **3** (100 mg, 1 equiv., 0.17 mmol) was dissolved in toluene (3 mL) in an ultrasonic bath, then triethylamine (0.024 mL, 1 equiv., 0.17 mmol) and diphenylphosphoryl azide (0.039 mL, 1 equiv., 0.17 mmol) were added. The reaction was carried out at room temperature (21 °C) for 2 h, after which ethanolamine (0.1026 mL, 10 equiv., 1.7 mmol) in 0.5 mL of toluene was added to the reaction mixture. Then, it was heated at 110 °C for 7 h. At the end of the reaction, the solvent was distilled in a rotary evaporator. The remainder was chromatographed with silica gel in a column. During elution of the column with ethyl acetate, a colorless substance (compound **19**) with a melting point of 202–204 °C was isolated. The yield was 126 mg (116%).

*^1^H NMR (400 MHz, CDCl_3_)*: δ 7.35–7.23 (m, 2H), 7.27–7.14 (m, 2H), 5.79 (s, 1H), 5.75 (q, *J* = 6.9 Hz, 1H), 5.18 (s, 1H), 4.69 (d, *J* = 2.3 Hz, 1H), 4.62 (t, *J* = 1.9 Hz, 1H), 4.12 (q, *J* = 7.2 Hz, 1H), 3.77–3.60 (m, 3H), 3.60–3.45 (m, 1H), 3.24 (s, 2H), 2.91 (d, *J* = 15.3 Hz, 1H), 2.68–2.60 (m, 1H), 2.55 (td, *J* = 11.0, 5.0 Hz, 1H), 2.46 (dd, *J* = 12.2, 8.1 Hz, 1H), 2.21–2.15 (m, 1H), 2.14 (d, *J* = 14.3 Hz, 1H), 2.08 (s, 1H), 2.03 (d, *J* = 12.8 Hz, 2H), 2.02–1.92 (m, 2H), 1.86–1.75 (m, 1H), 1.74 (s, 1H), 1.70 (s, 3H), 1.65 (d, *J* = 11.6 Hz, 1H), 1.62–1.54 (m, 1H), 1.57–1.47 (m, 4H), 1.47–1.35 (m, 1H), 1.38–1.28 (m, 1H), 1.28 (s, 4H), 1.26 (s, 9H), 1.30–1.19 (m, 1H), 1.13 (s, 3H), 1.19–1.03 (m, 2H), 1.02 (s, 3H), 0.96 (s, 3H), 0.96–0.85 (m, 2H), 0.89–0.81 (m, 2H), 0.79 (s, 3H).

*^13^C NMR (101 MHz, CDCl_3_)*: δ 174.49, 159.57, 149.75, 141.44, 140.91, 137.89, 128.73, 127.74, 126.14, 110.03, 64.03, 63.73, 59.28, 54.73, 49.53, 49.31, 47.47, 43.51, 42.07, 40.65, 38.86, 38.31, 37.51, 35.61, 33.85, 33.23, 31.93, 29.79, 29.71, 29.66, 29.37, 28.88, 27.43, 25.02, 23.58, 22.70, 21.49, 21.35, 19.23, 18.90, 16.35, 15.70, 14.41, 14.13.

*HRMS (ESI+)*: *m/z* calculated for C_40_H_60_N_5_O_2_ [M + H]^+^: 642.4742, found: 642.470.

*1′-((S)-1-Phenylethyl)-1H′-lup-2-eno-[2,3-d]-[1,2,3]-triazole-28-oic Acid derivative-3.* (**20**). Compound **3** (100 mg, 1 equiv., 0.17 mmol) was dissolved in toluene (3 mL) in an ultrasonic bath, then triethylamine (0.024 mL, 1 equiv., 0.17 mmol) and diphenylphosphoryl azide (0.039 mL, 1 equiv., 0.17 mmol) were added. The reaction was carried out at room temperature (21 °C) for 3 h, after which 2-amino-2-methyl-1-propanol (0.162 mL, 10 equiv., 1.7 mmol) in 0.5 mL of toluene was added to the reaction mixture. Then, it was heated at 110 °C for 3.5 h. At the end of the reaction, the solvent was distilled in a rotary evaporator. The remainder was chromatographed with silica gel in a column. During elution of the column with ethyl acetate, a colorless substance (compound **20**) with a melting point of 176–178 °C was isolated. The yield was 96 mg (84%).

*^1^H NMR (400 MHz, CDCl_3_)*: δ 7.30 (dd, *J* = 8.2, 6.3 Hz, 3H), 7.23–7.16 (m, 2H), 6.45 (s, 1H), 5.75 (q, *J* = 7.0 Hz, 1H), 5.25 (s, 1H), 5.05 (s, 1H), 4.65 (d, *J* = 2.3 Hz, 1H), 4.60 (t, *J* = 1.9 Hz, 1H), 3.55 (s, 2H), 2.92 (d, *J* = 15.2 Hz, 1H), 2.67 (dt, *J* = 13.4, 3.4 Hz, 1H), 2.44 (ddd, *J* = 20.1, 14.0, 9.6 Hz, 2H), 2.17 (d, *J* = 15.2 Hz, 1H), 2.08 (s, 0H), 2.02 (d, *J* = 7.0 Hz, 3H), 1.99–1.90 (m, 1H), 1.75 (td, *J* = 14.7, 14.1, 8.5 Hz, 2H), 1.68 (s, 3H), 1.62 (d, *J* = 11.7 Hz, 1H), 1.62–1.40 (m, 5H), 1.39–1.24 (m, 7H), 1.23 (d, *J* = 1.9 Hz, 6H), 1.09 (d, *J* = 22.1 Hz, 8H), 0.96 (s, 3H), 0.93–0.78 (m, 7H).

*^13^C NMR (101 MHz, CDCl_3_)*: δ 158.88, 149.68, 141.54, 140.91, 128.70, 127.69, 126.18, 109.99, 72.29, 63.89, 59.30, 54.78, 54.76, 49.51, 49.35, 47.37, 42.04, 40.62, 38.89, 38.36, 37.52, 35.61, 33.85, 33.24, 29.84, 29.55, 28.89, 27.40, 25.26, 25.22, 25.04, 23.60, 21.50, 21.35, 19.29, 18.89, 16.33, 15.74, 14.45.

*HRMS (ESI+)*: *m/z* calculated for C_42_H_64_N_5_O_2_ [M + H]^+^: 670.5055, found: 670.5093.

*1′-((S)-1-Phenylethyl)-1H′-lup-2-eno-[2,3-d]-[1,2,3]-triazole-28- oic Acid derivative-4.* (**21**). Compound **3** (100 mg, 1 equiv., 0.17 mmol) was dissolved in toluene (3 mL) in an ultrasonic bath, then triethylamine (0.024 mL, 1 equiv., 0.17 mmol) and diphenylphosphoryl azide (0.039 mL, 1 equiv., 0.17 mmol) were added. The reaction was carried out at room temperature (22 °C) for 3 h, after which 3-amino-1,2-propanediol (0.1317 mL, 10 equiv., 1.7 mmol) was added to the reaction mixture. Then, it was heated at 110 °C for 2 h. At the end of the reaction, the solvent was distilled in a rotary evaporator. The remainder was chromatographed with silica gel in a column. Elution of the column with a mixture of ethyl acetate and methanol (10:1) isolated a colorless substance (compound **21**) with a melting point of 182–185 °C. The yield was 89 mg (78%).

*^1^H NMR (400 MHz, CDCl_3_)*: δ 7.35–7.23 (m, 3H), 7.18 (td, *J* = 7.3, 1.9 Hz, 2H), 5.95 (s, 1H), 5.76 (q, *J* = 7.3 Hz, 1H), 5.35–5.18 (m, 1H), 4.68 (s, 1H), 4.64–4.57 (m, 1H), 4.12 (q, *J* = 7.2 Hz, 1H), 3.82–3.70 (m, 1H), 3.70–3.58 (m, 1H), 3.59–3.39 (m, 2H), 3.26 (dt, *J* = 16.1, 5.3 Hz, 2H), 2.92 (dd, *J* = 20.5, 15.2 Hz, 1H), 2.69–2.59 (m, 1H), 2.54 (ddq, *J* = 16.3, 11.8, 6.9, 5.2 Hz, 1H), 2.42 (dd, *J* = 12.0, 8.1 Hz, 1H), 2.24–2.10 (m, 1H), 2.06 (d, *J* = 10.6 Hz, 1H), 2.01 (d, *J* = 6.9 Hz, 3H), 1.97–1.71 (m, 1H), 1.69 (s, 3H), 1.67–1.30 (m, 5H), 1.30–1.19 (m, 7H), 1.12 (d, *J* = 8.0 Hz, 4H), 1.03–0.89 (m, 6H), 0.89–0.80 (m, 1H), 0.78 (s, 2H).

*^13^C NMR (101 MHz, CDCl_3_)*: δ 159.68, 149.68, 141.37, 140.94, 138.02, 128.76, 128.68, 127.79, 126.18, 126.08, 110.09, 71.98, 63.85, 63.83, 63.30, 59.27, 54.71, 49.45, 49.31, 47.39, 42.50, 42.06, 40.63, 38.86, 38.29, 37.50, 35.72, 33.86, 33.22, 30.84, 29.71, 28.85, 27.41, 25.62, 25.01, 23.63, 23.52, 21.52, 21.34, 19.22, 18.89, 16.40, 15.68, 14.43, 14.20.

*HRMS (ESI+)*: *m/z* calculated for C_41_H_62_N_5_O_3_ [M + H]^+^: 672.4847, found: 672.4858.

*1′-((S)-1-Phenylethyl)-1H′-lup-2-eno-[2,3-d]-[1,2,3]-triazole-28-oic Acid derivative-5.* (**22**). Compound **3** (100 mg, 1 equiv., 0.17 mmol) was dissolved in toluene (3 mL) in an ultrasonic bath, then triethylamine (0.024 mL, 1 equiv., 0.17 mmol) and diphenylphosphoryl azide (0.039 mL, 1 equiv., 0.17 mmol) were added. The reaction was carried out at room temperature (22 °C) for 3 h, after which 2-(2-aminoethoxy)ethanol (0.1705 mL, 10 equiv., 1.7 mmol) in 0.5 mL of toluene was added to the reaction mixture. Then, it was heated at 110 °C for 2 h. At the end of the reaction, the solvent was distilled in a rotary evaporator. The remainder was chromatographed with silica gel in a column. Elution of the column with a mixture of ethyl acetate and methanol (20:1) isolated a colorless substance (compound **22**) with a melting point of 165–167 °C. The yield was 76 mg (65%).

*^1^H NMR (400 MHz, CDCl_3_)*: δ 7.37–7.23 (m, 3H), 7.22–7.16 (m, 2H), 5.74 (q, *J* = 7.0 Hz, 1H), 5.49 (s, 1H), 4.76–4.67 (m, 2H), 4.62 (t, *J* = 1.9 Hz, 1H), 3.83–3.68 (m, 3H), 3.68–3.60 (m, 3H), 3.60–3.51 (m, 4H), 3.36 (d, *J* = 5.1 Hz, 2H), 2.93 (d, *J* = 15.2 Hz, 1H), 2.71–2.61 (m, 1H), 2.49 (ddt, *J* = 20.7, 12.4, 6.5 Hz, 2H), 2.16 (d, *J* = 15.2 Hz, 1H), 2.06 (d, *J* = 8.7 Hz, 0H), 2.01 (d, *J* = 7.0 Hz, 3H), 1.76 (d, *J* = 11.2 Hz, 2H), 1.70 (s, 3H), 1.68–1.28 (m, 7H), 1.27 (s, 7H), 1.19–1.02 (m, 7H), 0.96 (s, 3H), 0.90–0.82 (m, 5H), 0.80 (s, 3H).

*^13^C NMR (101 MHz, CDCl_3_)*: δ 158.08, 149.69, 141.63, 140.96, 137.71, 128.69, 127.65, 126.19, 110.06, 72.36, 70.81, 63.60, 61.66, 61.14, 59.30, 54.74, 49.50, 49.27, 47.52, 42.07, 41.35, 40.63, 40.18, 38.87, 38.32, 37.63, 35.71, 33.83, 33.25, 29.81, 29.77, 28.88, 27.39, 25.03, 23.67, 22.63, 21.46, 21.35, 19.26, 18.91, 16.32, 15.71, 14.42.

*HRMS (ESI+)*: *m/z* calculated for C_42_H_64_N_5_O_3_ [M + H]^+^: 686.5004, found: 686.4954.

### 3.3. In Vitro Biological Assays

#### 3.3.1. Antimicrobial Activity

The antimicrobial activity of the samples was evaluated in relation to strains of the Gram-positive bacteria *Staphylococcus aureus* and *Bacillus subtilis*, the Gram-negative bacteria *Escherichia coli* and *Pseudomonas aeruginosa*, and the yeast fungus *Candida albicans* by serial dilution with determination of the minimum inhibitory concentration (MIC). The test strains of the microorganisms used in the study were obtained from the American Type Culture Collection. An antibacterial drug, ceftriaxone; benzylpenicillin sodium salt; and an antifungal drug, nystatin, were used as comparison drugs and served as positive controls.

MIC was determined by the method of serial dilution of ethanol solutions of the test samples in a nutrient broth. Suspensions of test strains at a concentration of 106 CFU/mL were used to carry out the method of serial dilutions. A suspension of test strains of the microorganisms was prepared from daily cultures grown on mown agar at a temperature of 37 °C for 24 h and at 30 °C for 48 h for the yeast fungus *Candida albicans*. The antimicrobial activity of the samples was studied at dilutions in the range of 1.56–50 µg/mL. A quantity of 0.1 mL of microbial suspension at a concentration of 106 CFU/mL was added to each tube with a working dilution of each test sample. The procedure was repeated for all cultures tested. A suspension of microbes with a nutrient medium without a sample was placed in control tubes to serve as a negative control. The mixture was incubated in a thermostat for 24–48 h, depending on the class of microorganism.

Then, the presence of turbidity in each of the tubes was visually determined, and the one that contained a transparent suspension and the lowest concentration of the antimicrobial agent was selected. This concentration corresponded to the MIC. The results were averaged according to the data of three experiments.

#### 3.3.2. Cytotoxic Activity

The cytotoxicity of the synthesized compounds against *Artemia salina* (Leach) was evaluated in accordance with the methodology proposed by Meyer et al. [49].

The cytotoxicity of the samples was evaluated in the survival test of larvae of *Artemia salina* (Leach) crustaceans.

The experiments were carried out on larvae of 2 days of age under in vitro cultivation conditions. The larvae were grown by immersing the eggs of *Artemia salina* (Leach) crustaceans in artificial seawater and incubating them for 48 h at a temperature of 37 °C. The samples were dissolved in 2 mL of ethanol, then 500 µL (3 parallels), 50 µL (3 parallels) and 5 µL (3 parallels) were taken from this solution. After evaporation of ethanol, 5 mL of artificial seawater was added to each bottle.

Thus, if the initial weight of the sample was 2 mg, then the final concentrations of the sample were 100 µg/mL, 10 µg/mL and 1 µg/mL, respectively, with 3 repetitions for each concentration. Ten larvae of 2-day-old *Artemia salina* crustaceans were planted in each vial using a Pasteur pipette. After that, all the vials were left at room temperature in the light for 24 h.

After 24 h, the surviving and dead larvae were counted. Then, using the obtained data on the upper and lower toxic limits, the half-toxic dose of the sample was calculated. The control was DMSO in equal amounts.

The test was performed using ready-made samples as well as a comparison drug, dactinomycin (actinomycin D), which had antitumor (cytotoxic) activity (producer: Sigma Aldrich, St. Louis, MO, USA).

Lethal concentrations of these compounds, leading to 50% death of the shrimp (LC_50_), and 95% confidence intervals were determined based on 24 h calculations with probit analysis, and LC_50_ values were obtained with a 95% confidence interval [49].

## 4. Conclusions

Thus, in the synthesis of heterocyclic derivatives based on lupane-structured triterpenoid betulonic acid, 21 compounds were obtained, 18 of which were new. The physico-chemical properties of the synthesized compounds were proven by spectroscopic methods. As a result of the study of biological activity, it was found that most of the tested compounds exhibited antimicrobial and cytotoxic activity. Compound **6** showed pronounced antimicrobial activity against the Gram-positive *Staphylococcus aureus* test strain ATCC 6538, and compound **7** showed pronounced antimicrobial activity against the Gram-negative *Escherichia coli* test strain ATCC 25922.

## Figures and Tables

**Table 1:** Antimicrobial activity (MIC values in µg/mL) of test compounds in relation to reference test strains.

Compounds	*Staphylococcus aureus* ATCC 6538	*Bacillus* *subtilis* ATCC 6633	*Escherichia* *coli* ATCC 25922	*Pseudomonas* *aeruginosa* ATCC 27853	*Candida* *albicans* ATCC 10231
**Betulonic acid 2**	50	25	-	-	50
**3**	25	50	12.5	-	50
**4**	25	50	50	-	-
**5**	50	25	25	-	50
**6**	6.3	-	25	-	-
**7**	25	25	6.3	50	25
**8**	25	50	-	-	-
**9**	50	-	-	-	-
**10**	50	50	-	-	-
**13**	25	-	50	-	-
**14**	50	-	-	-	-
**15**	12.5	50	25	-	-
**16**	50	-	50	-	-
**17**	50	50	50	-	-
**18**	25	50	12.5	-	-
**19**	25	25	50	-	25
**20**	50	25	25	-	50
**21**	25	-	50	-	-
**22**	50	-	-	-	-
**Ceftriaxone**	6.3	12.5	6.3	6.3	-
**Benzylpenicillin sodium salt**	12.5	25	25	50	-
**Nystatin**	-	-	-	-	12.5

**Table 2:** Cytotoxic activity of the compounds.

Compounds	LD_50_, µg/mL
**Betulonic acid 2**	88.7
**3**	82.5
**4**	105.7
**5**	-
**6**	82.8
**7**	89.4
**8**	107.2
**9**	95.6
**10**	79.4
**13**	97.3
**14**	90.5
**15**	105.9
**16**	85.1
**17**	-
**18**	66.2
**19**	74.1
**20**	63.5
**21**	-
**22**	69.0
Comparison drug: dactinomycin (*actinomycin D*)	46.2

## Data Availability

The data presented in this study are available on request from the corresponding author.

## Supplementary Materials

The following supporting information can be downloaded at: https://www.mdpi.com/article/10.3390/molecules29133149/s1, NMR, HRMS spectra.
