# Supplementary material for: Synthesis and Investigation of Biological Activity of New Betulonic Acid Derivatives Containing 1,2,3-Triazole Fragments
Source: Molecules. 2024 Jul 2;29(13):3149. doi: 10.3390/molecules29133149 (PMC11243376; doi:10.3390/molecules29133149)

Supporting Information for

Synthesis and Investigation of Biological Activity of New Betulonic Acid Derivatives Containing 1,2,3-Triazole Fragments

List of Contents

^{1}H, ^{13}C NMR and Mass-Spectra

Compounds 2–22

Compound **2**, ^{1}H NMR (400 MHz, $CDCl_3$)

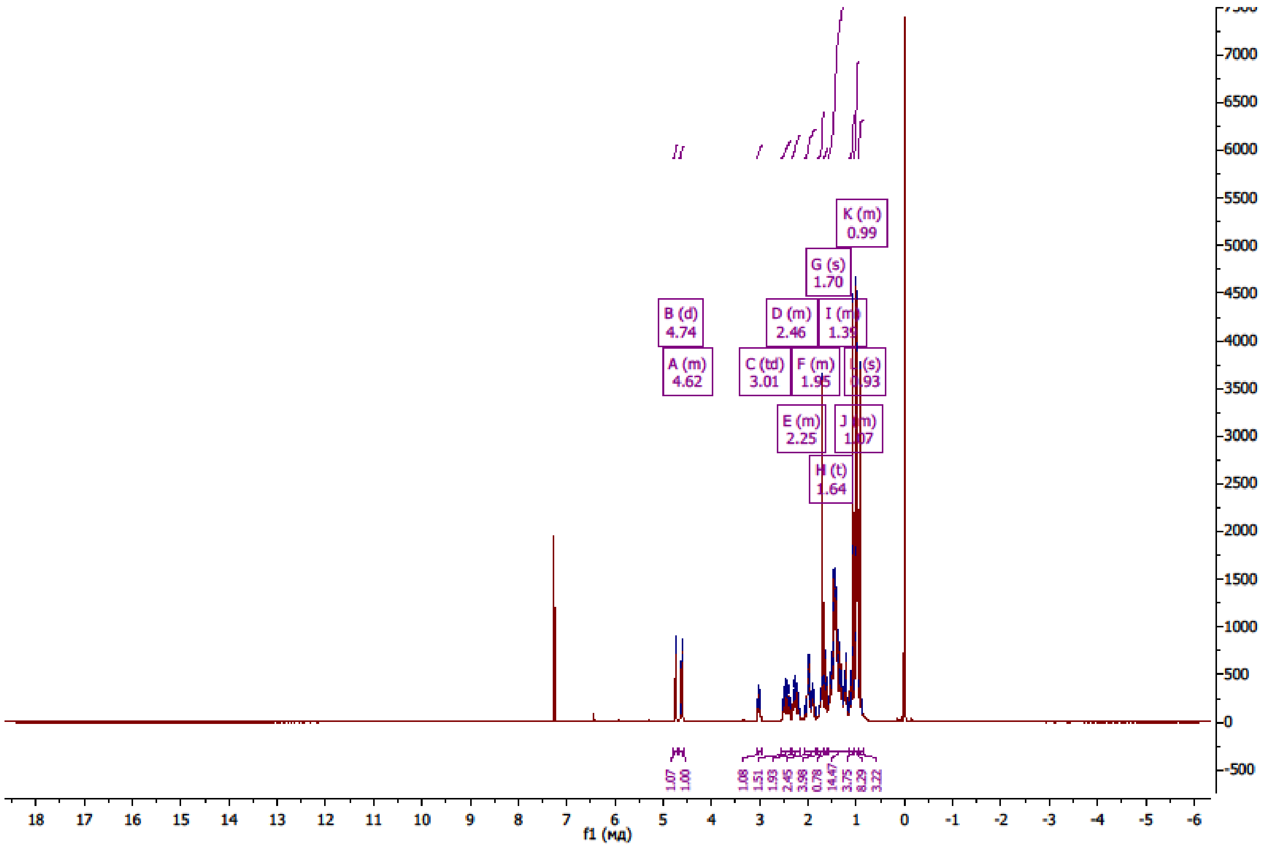

^{13}C NMR (101 MHz, $CDCl_3$)

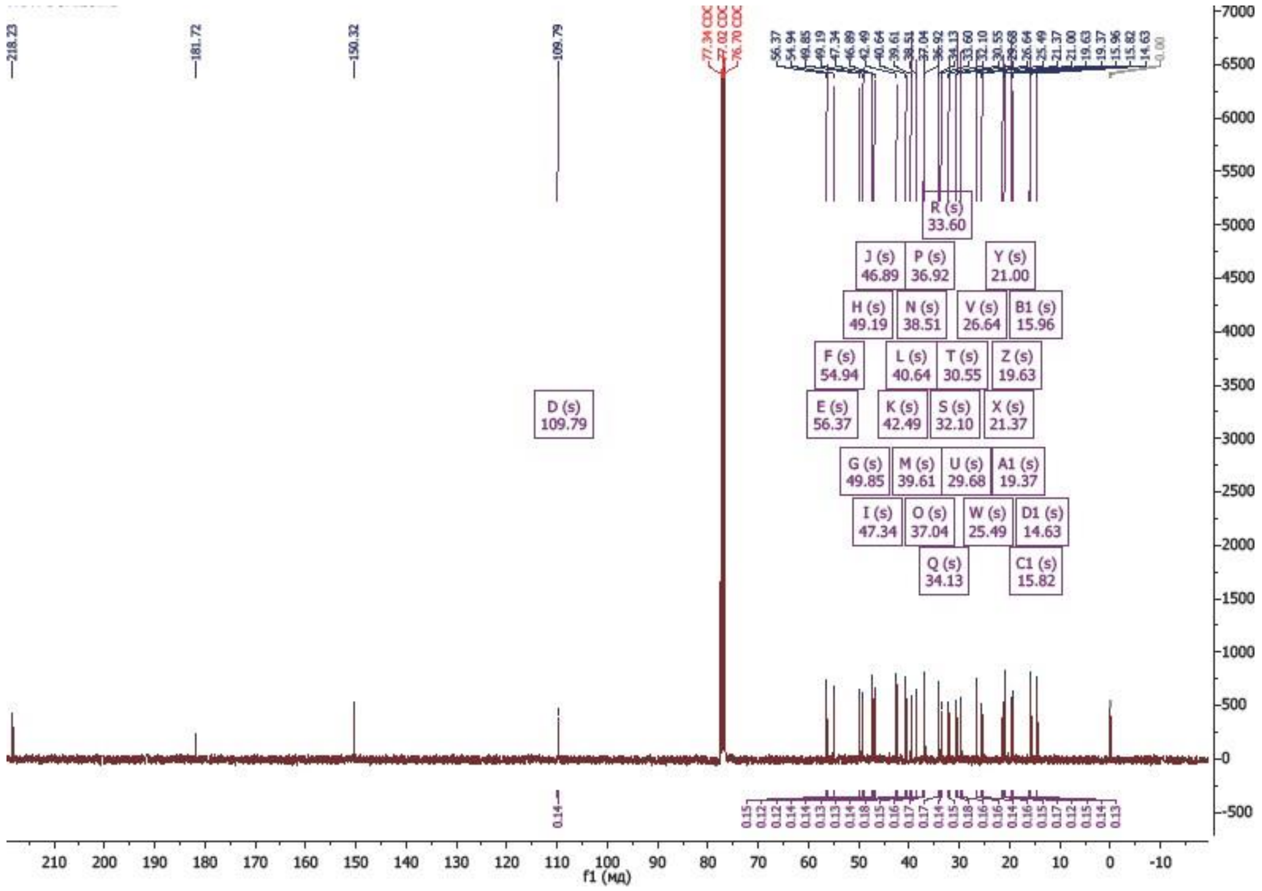

Compound **3**, ^{1}H NMR (400 MHz, $CDCl_3$)

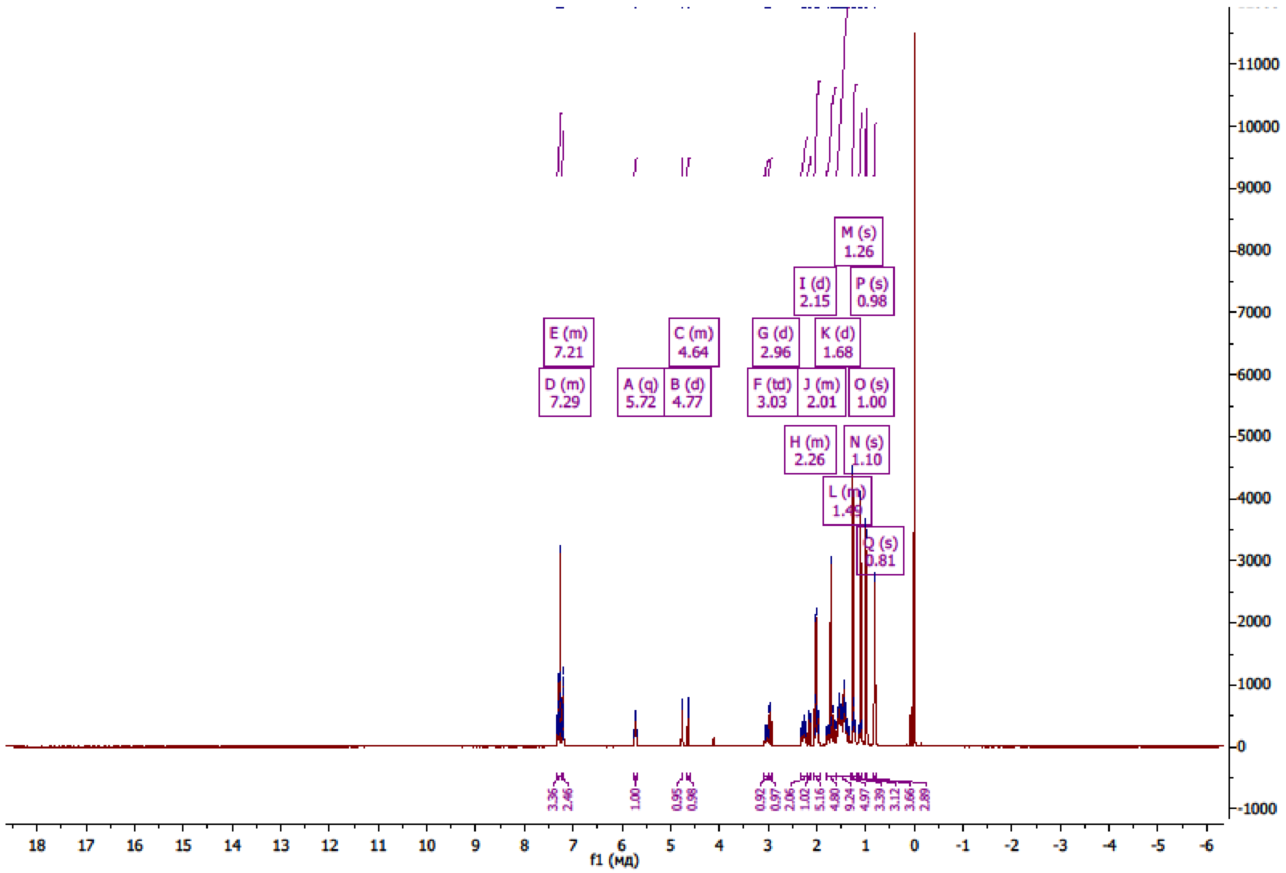

^{13}C NMR (101 MHz, $CDCl_3$)

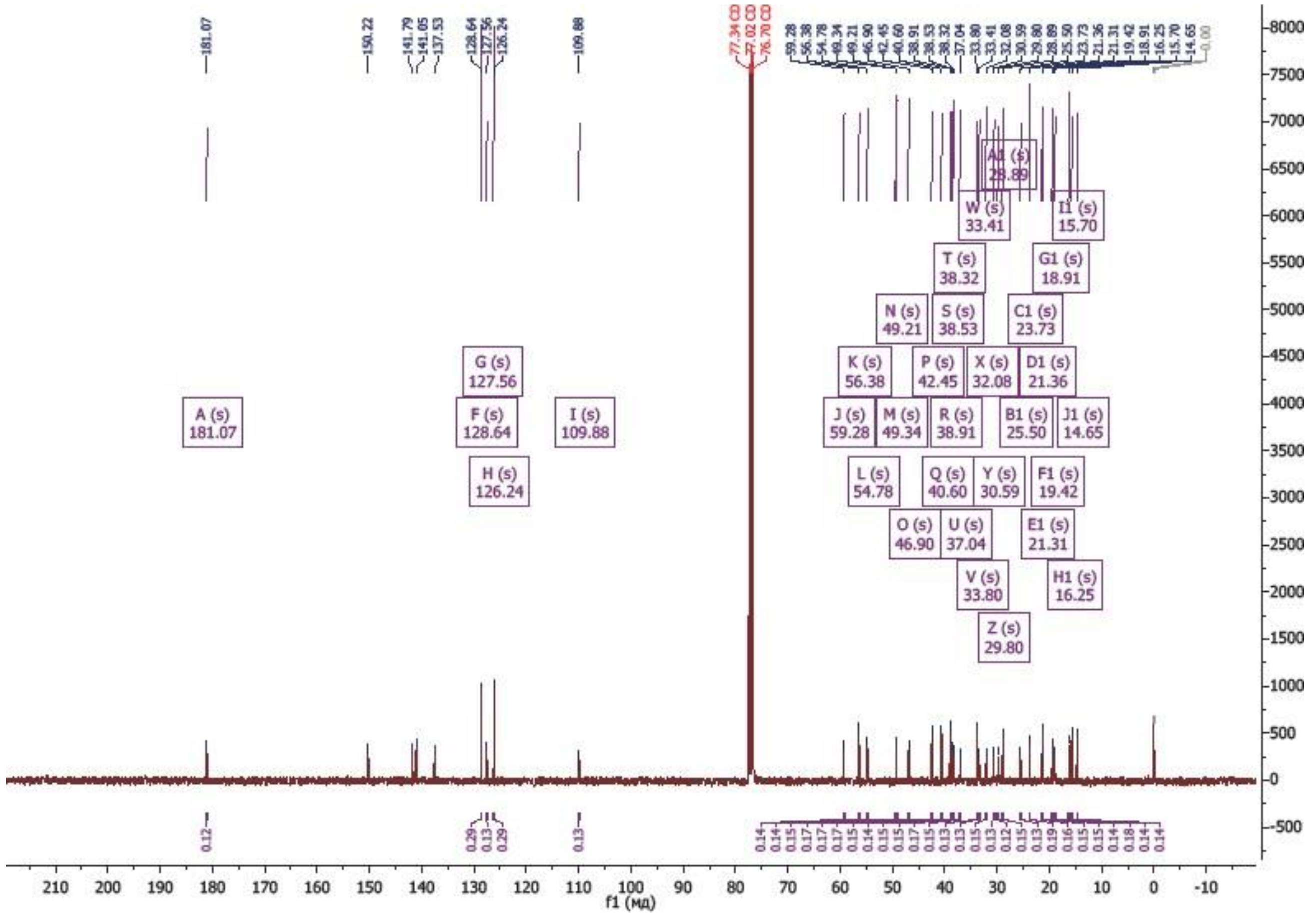

Compound **4**, ^{1}H NMR (400 MHz, $CDCl_3$)

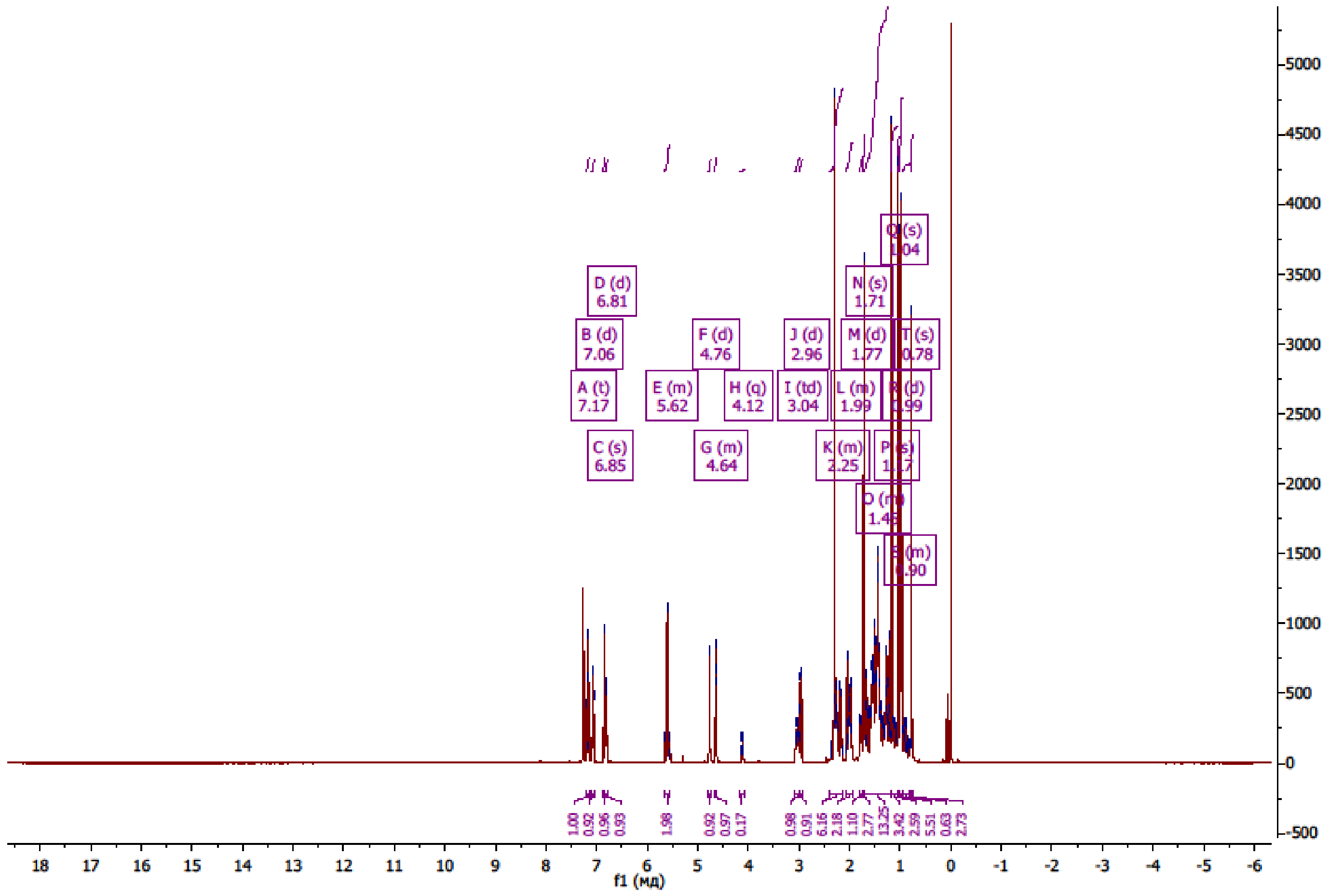

^{13}C NMR (101 MHz, $CDCl_3$)

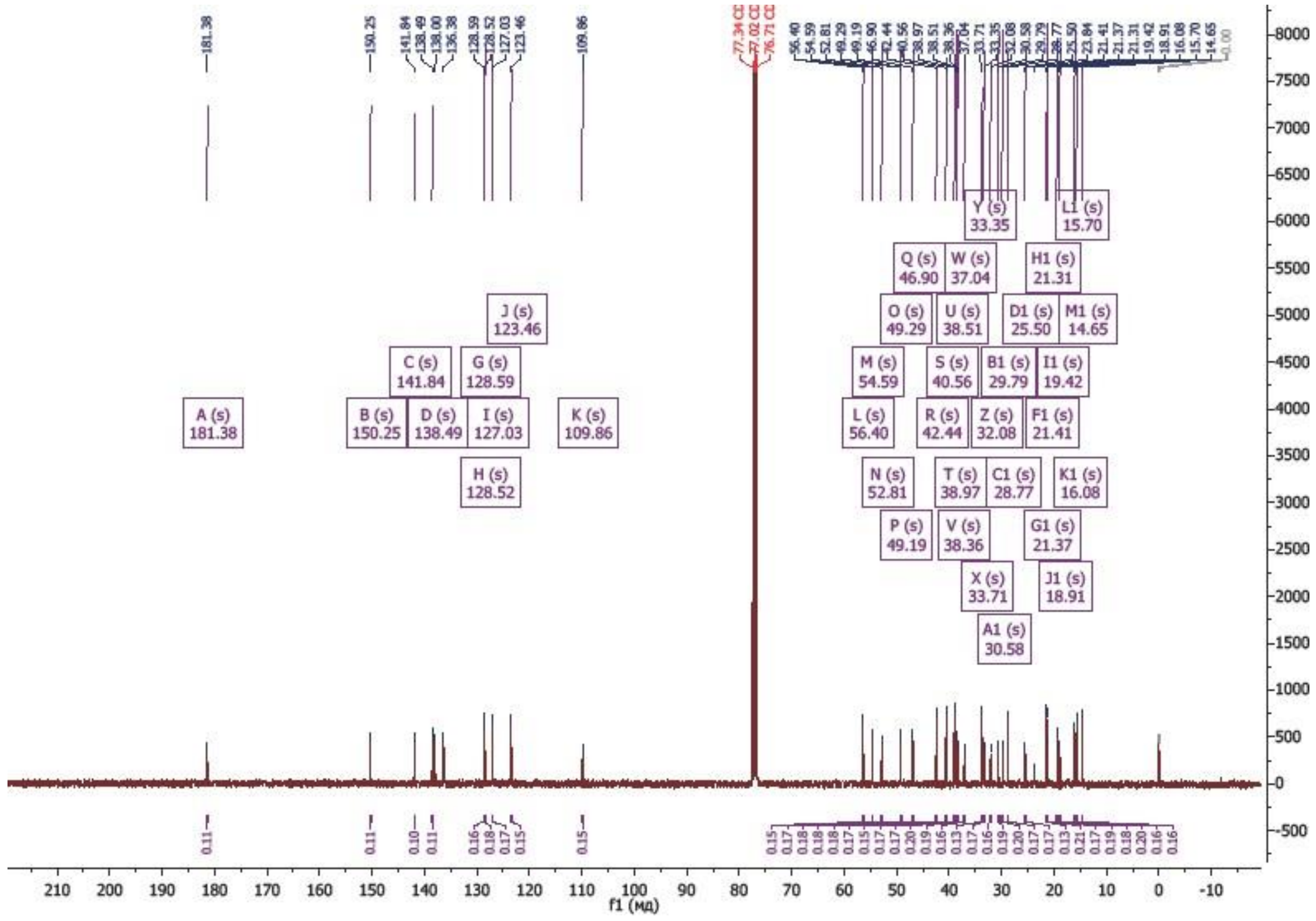

Compound **5**, ^{1}H NMR (400 MHz, $CDCl_3$)

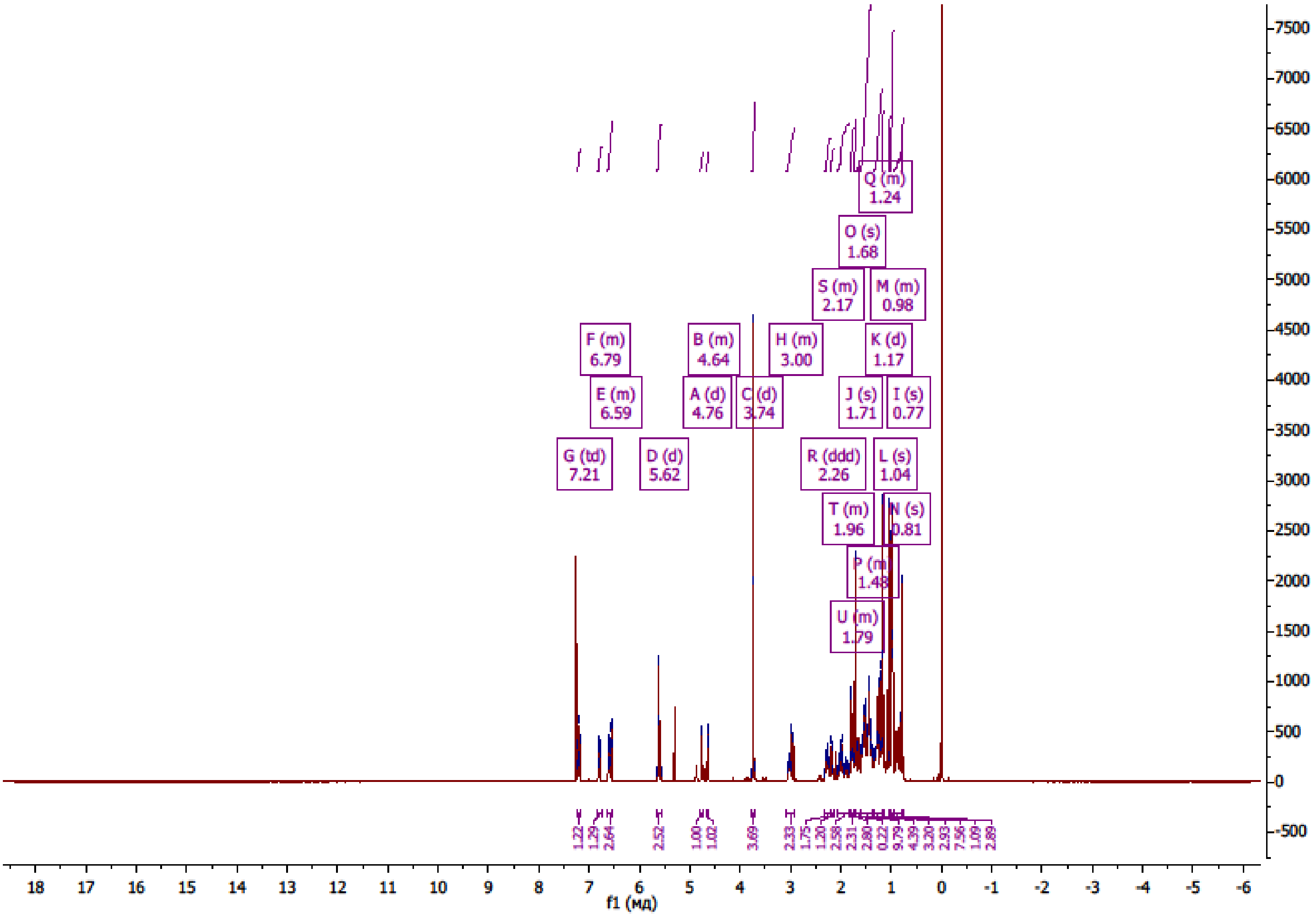

^{13}C NMR (101 MHz, $CDCl_3$)

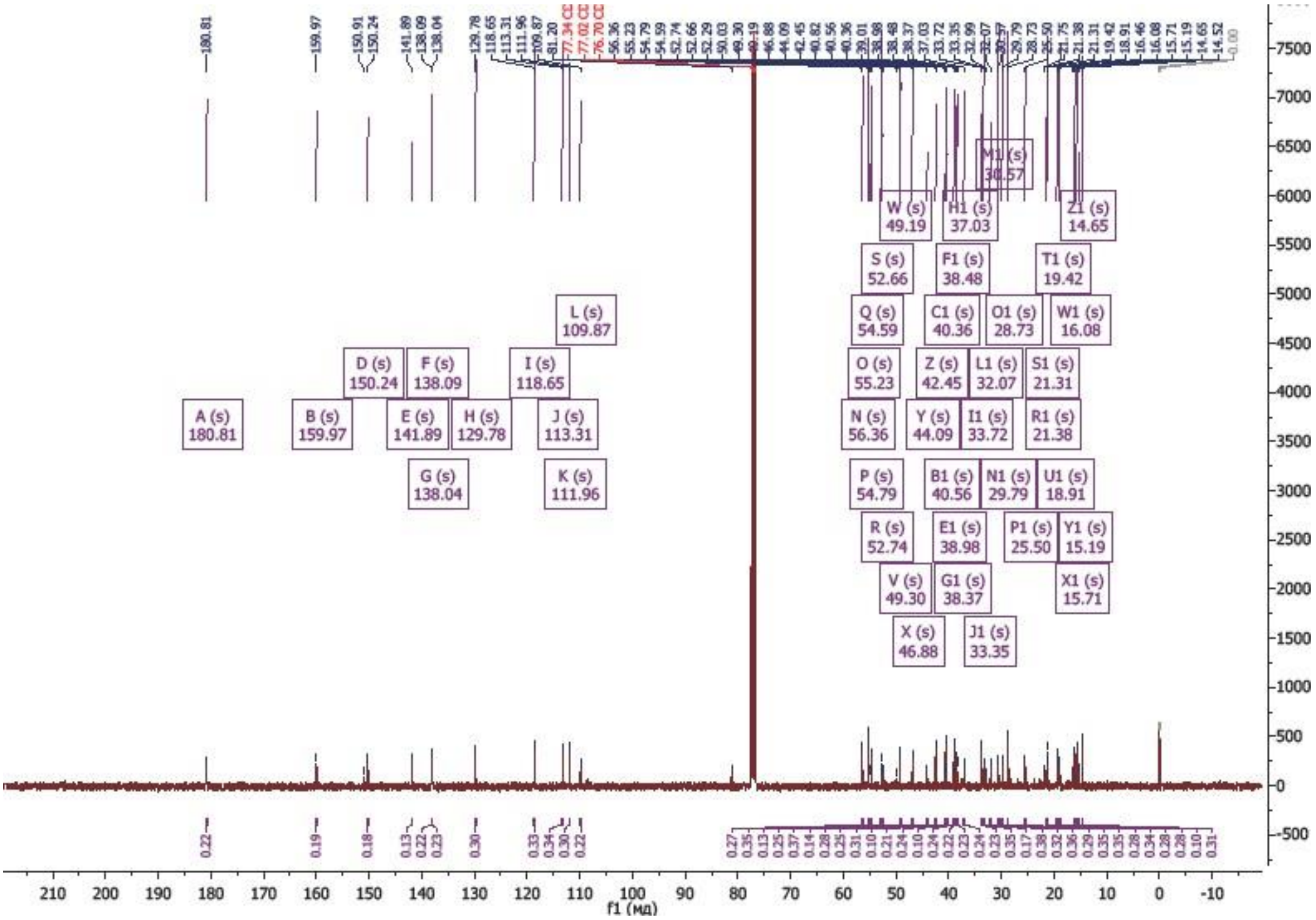

Compound **6**, ^{1}H NMR (400 MHz, $CDCl_3$)

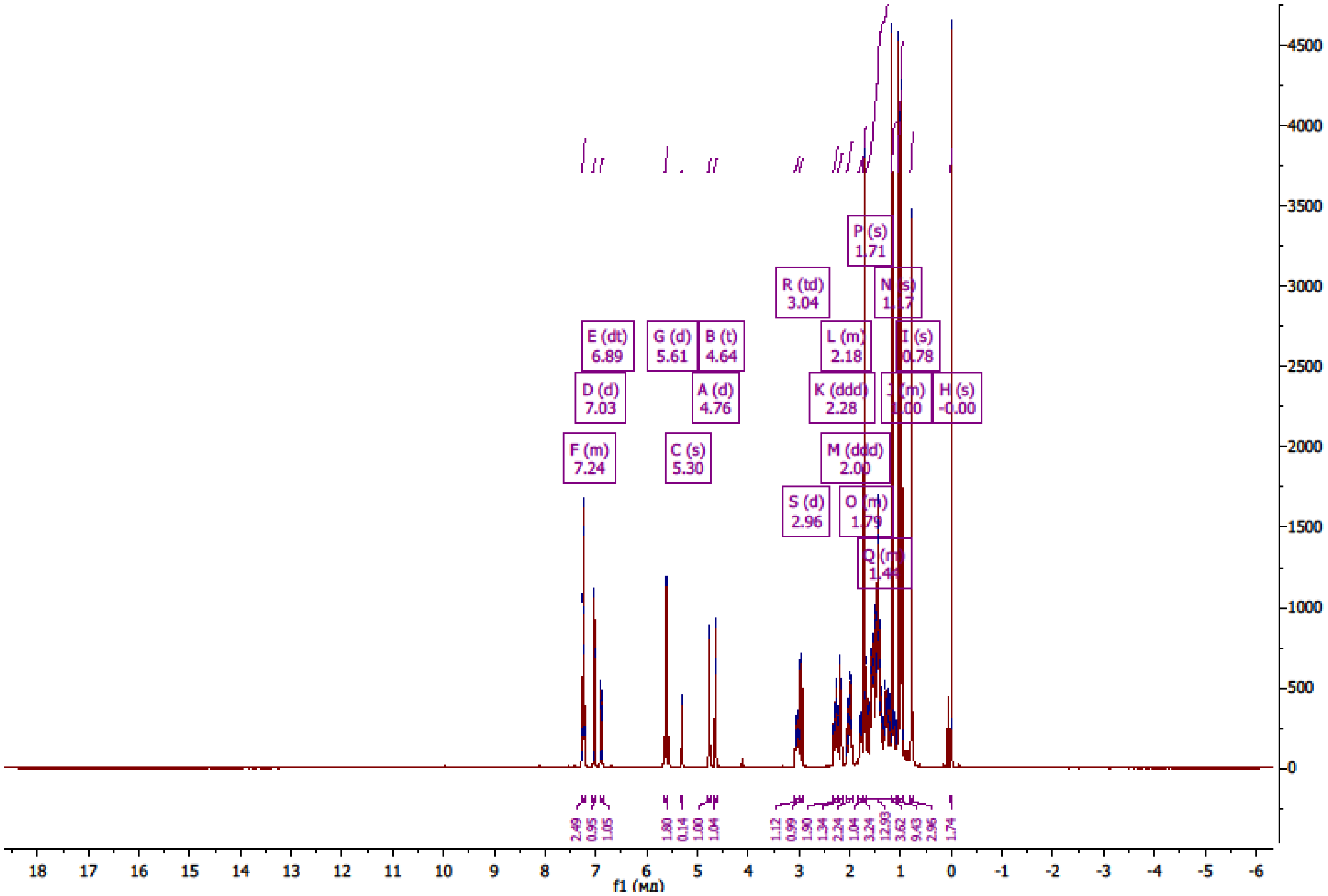

^{13}C NMR (101 MHz, $CDCl_3$)

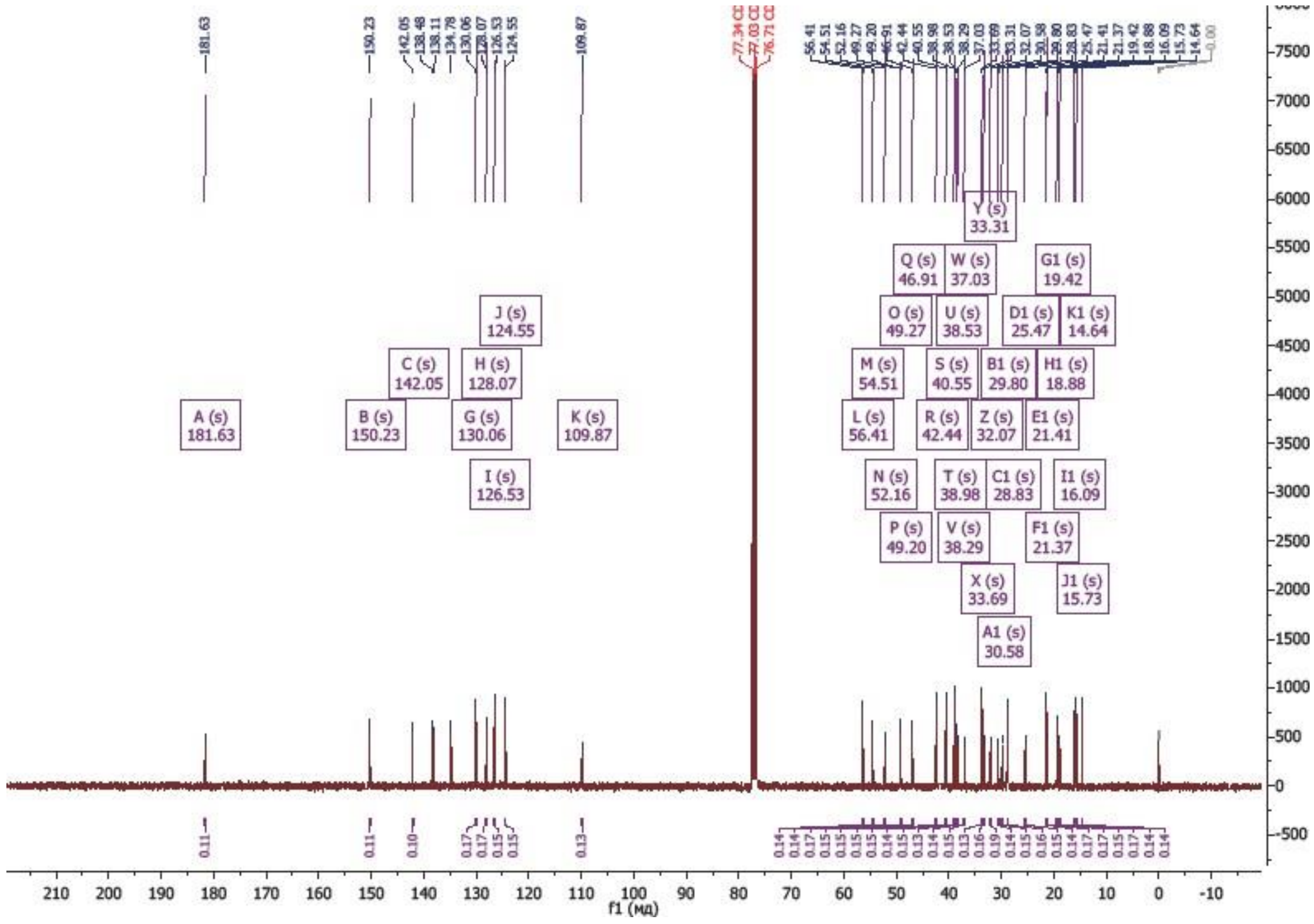

Compound **7**, ^{1}H NMR (400 MHz, $CDCl_3$)

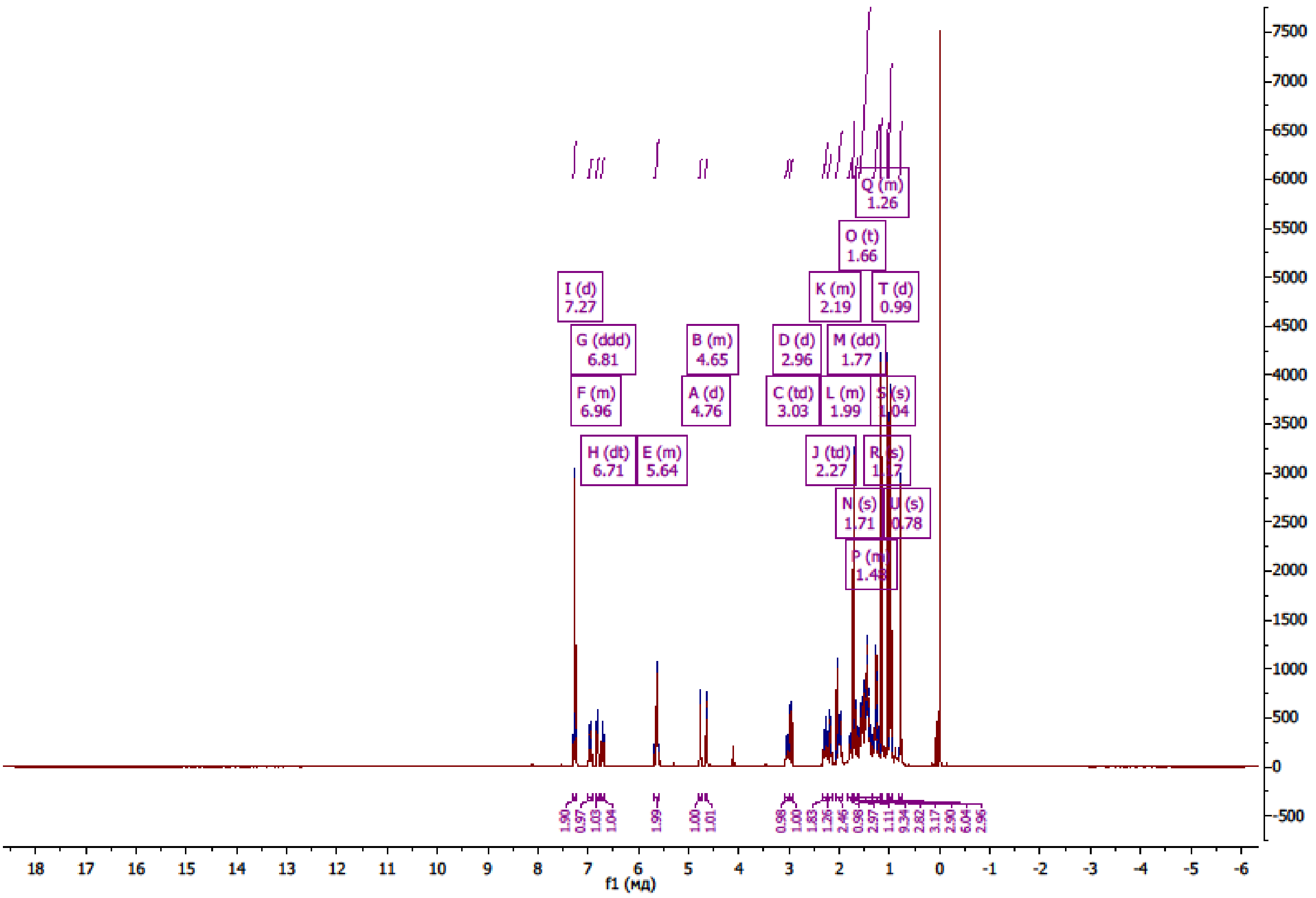

^{13}C NMR (101 MHz, $CDCl_3$)

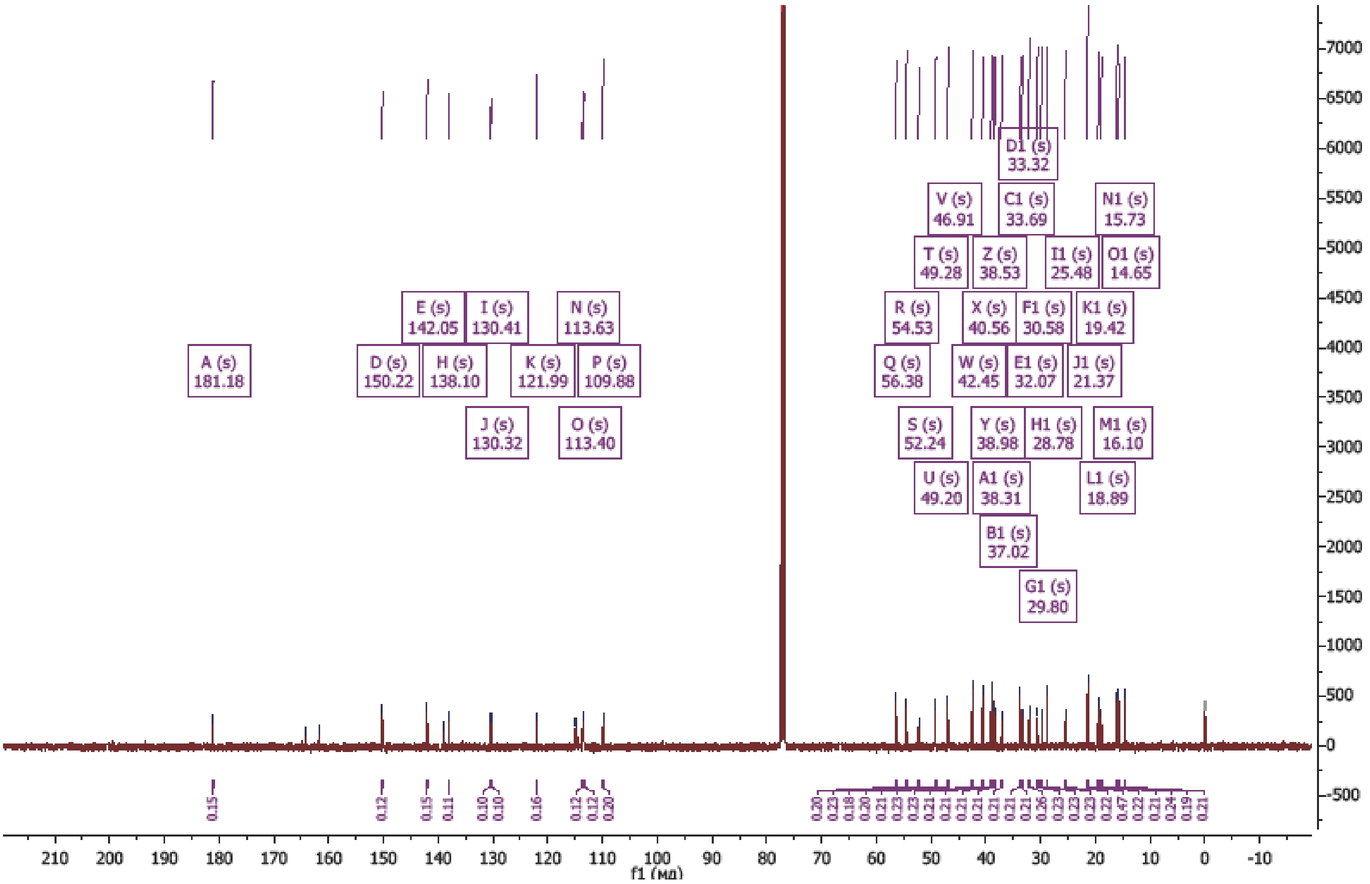

Compound **8**, ^{1}H NMR (400 MHz, $CDCl_3$)

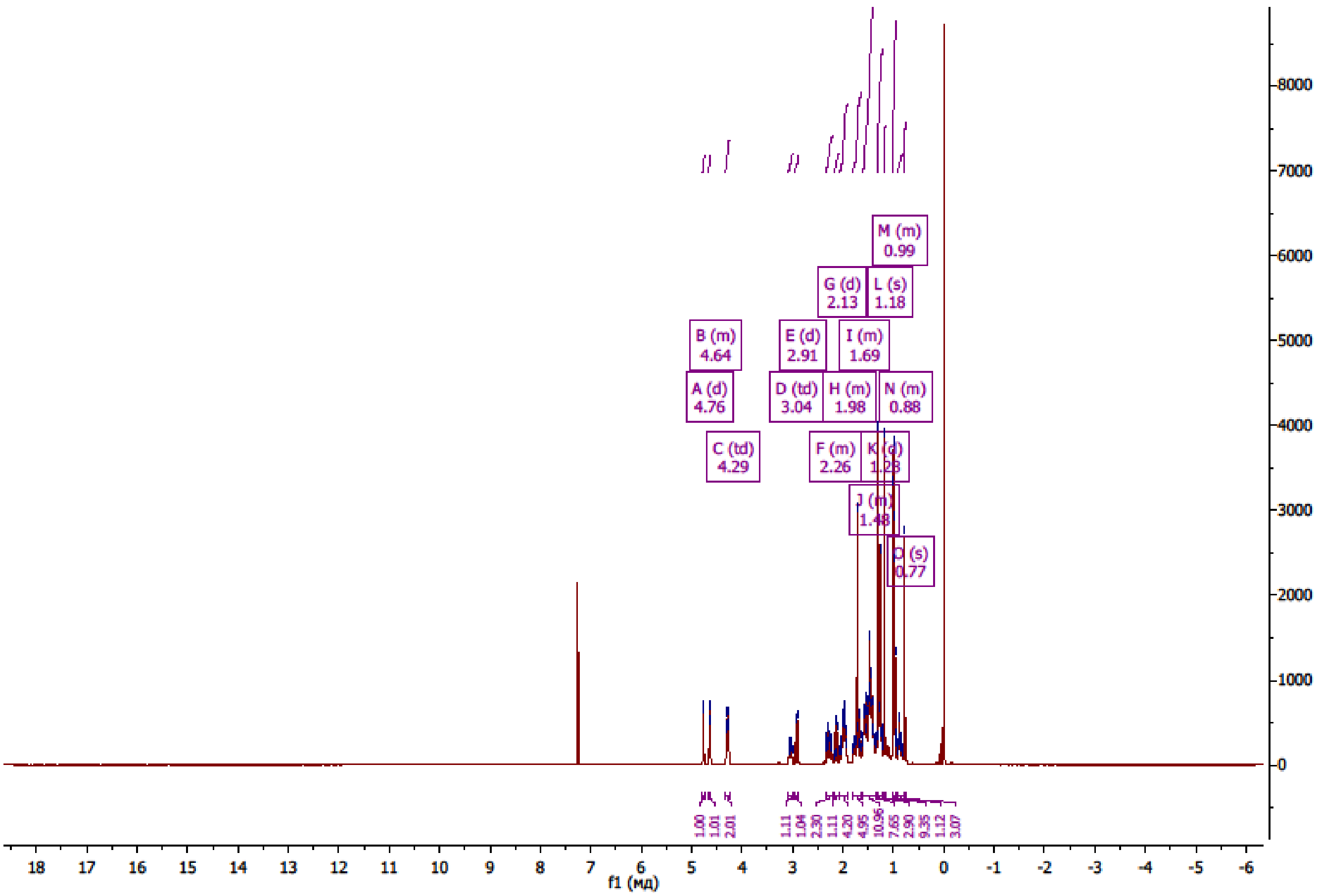

^{13}C NMR (101 MHz, $CDCl_3$)

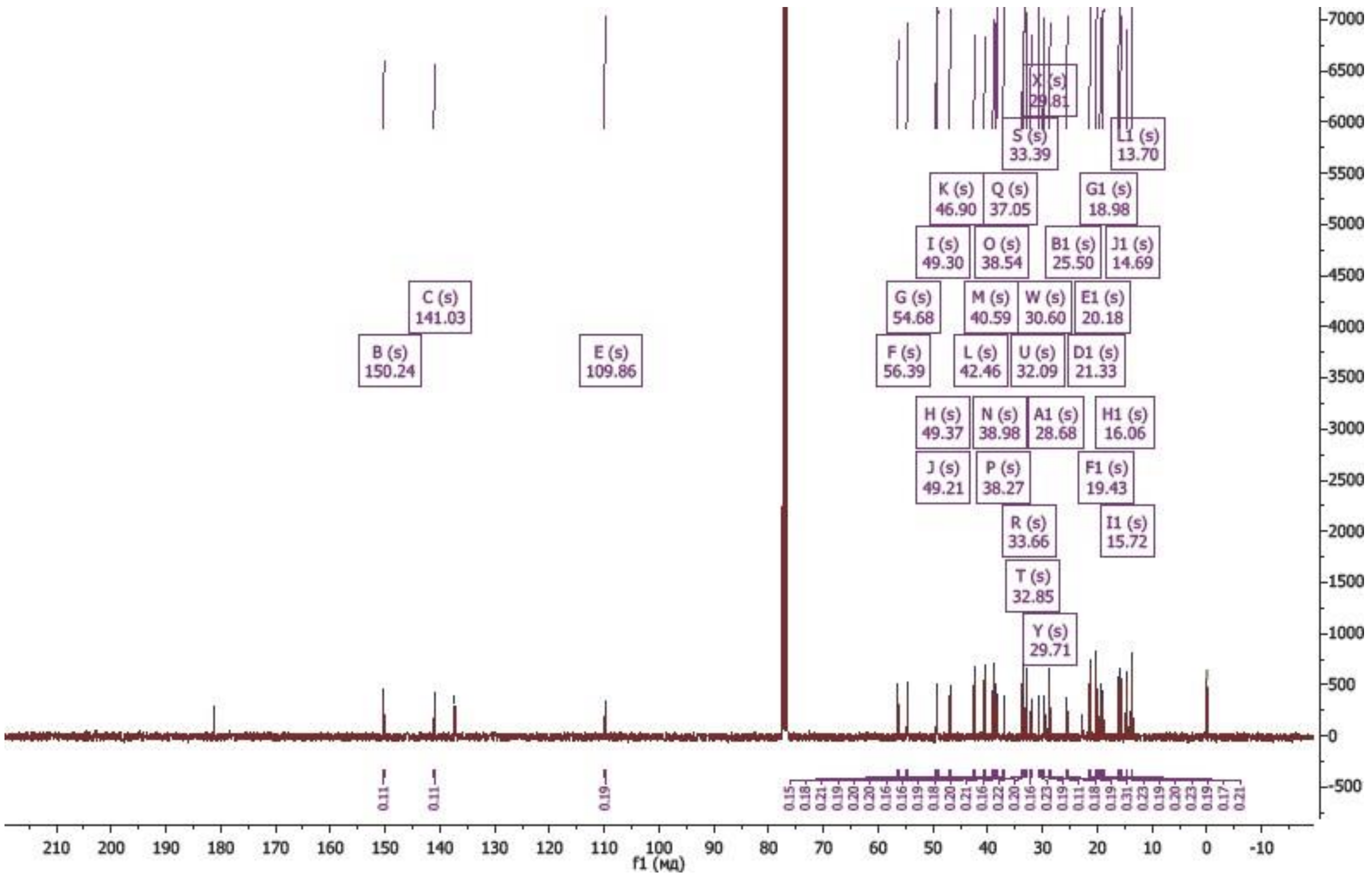

Compound **9**, ^{1}H NMR (400 MHz, $CDCl_3$)

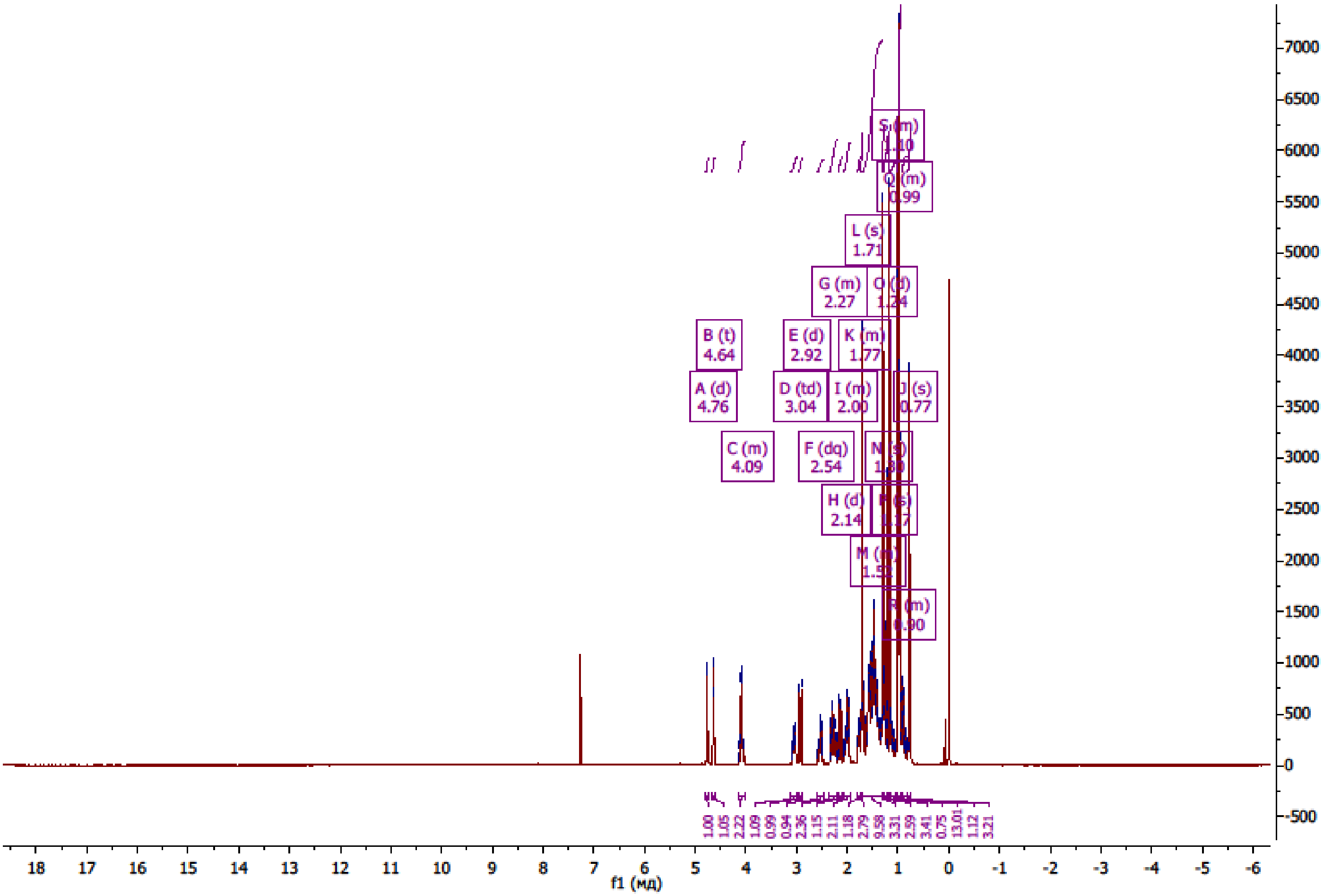

^{13}C NMR (101 MHz, $CDCl_3$)

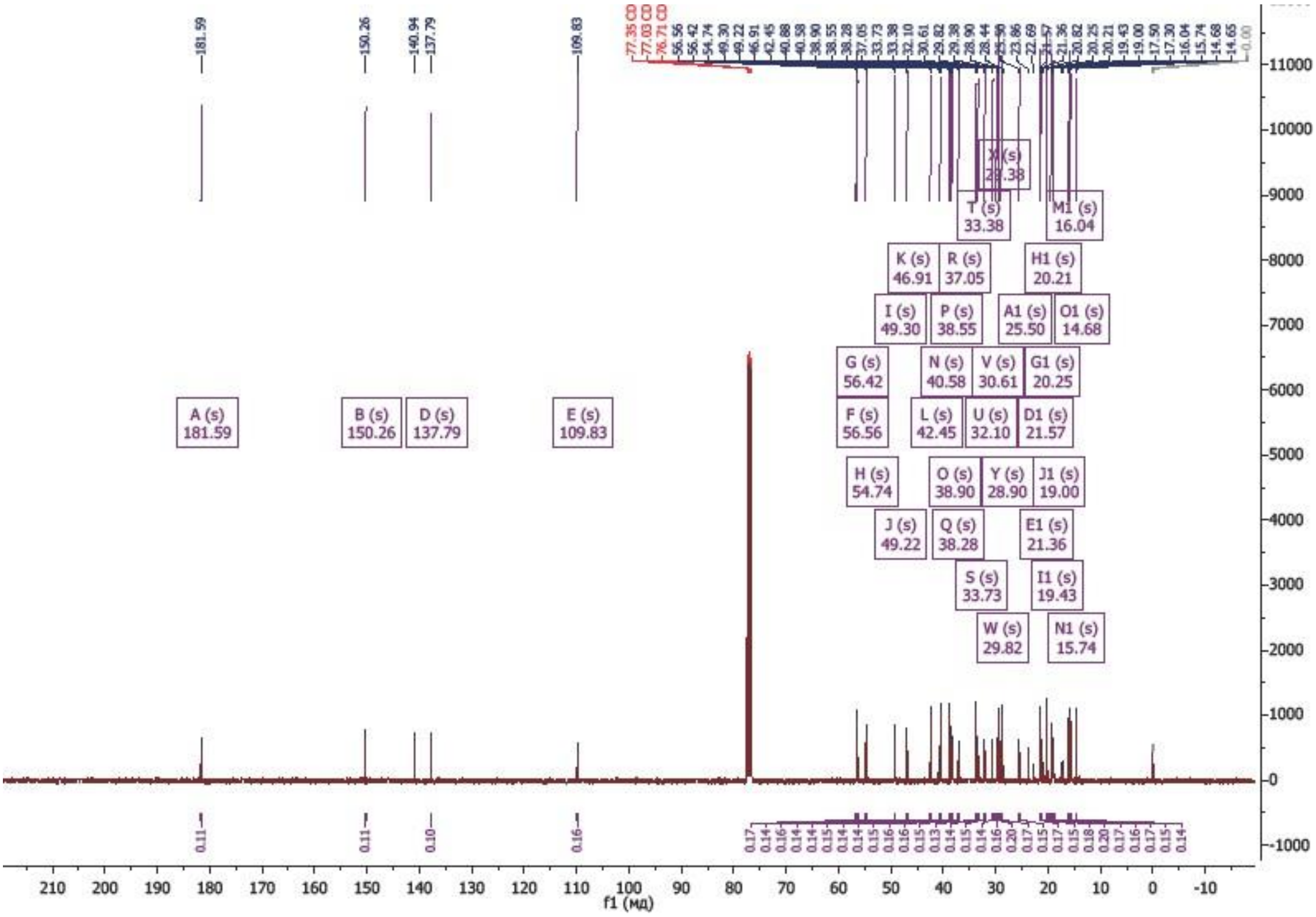

Compound **10**, ^{1}H NMR (400 MHz, $CDCl_3$)

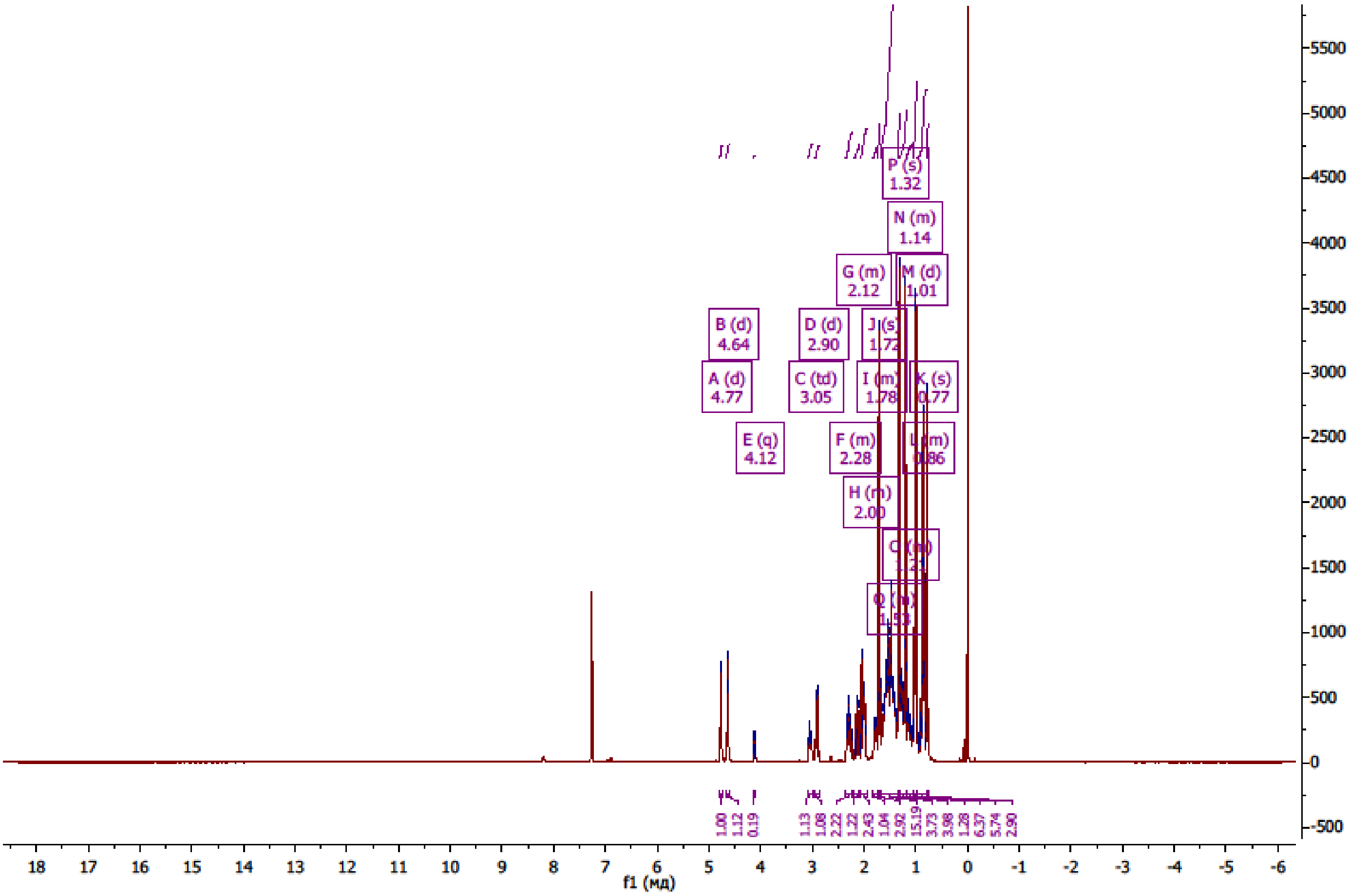

^{13}C NMR (101 MHz, $CDCl_3$)

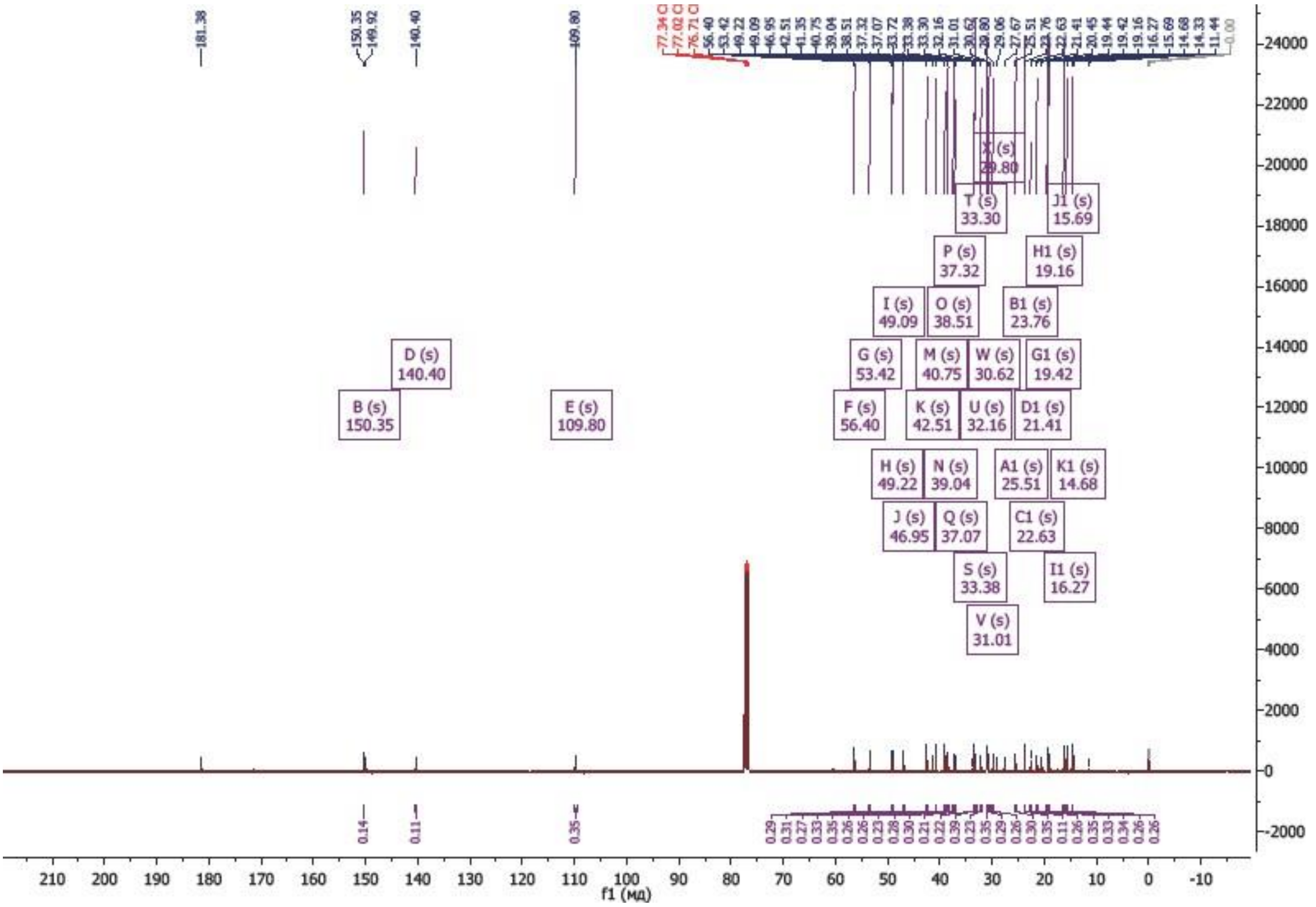

Compound **11**, ^{1}H NMR (400 MHz, $CDCl_3$)

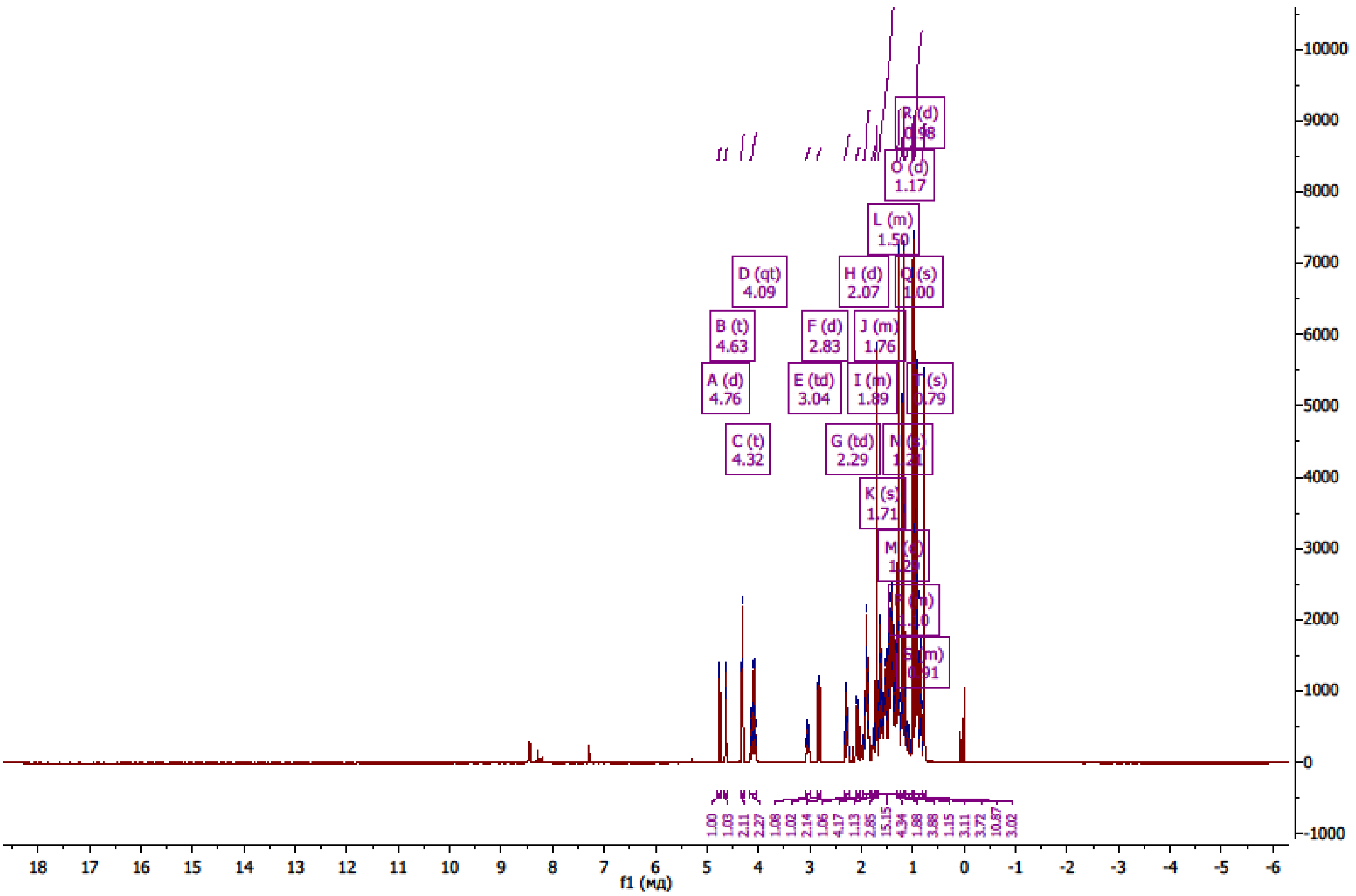

^{13}C NMR (101 MHz, $CDCl_3$)

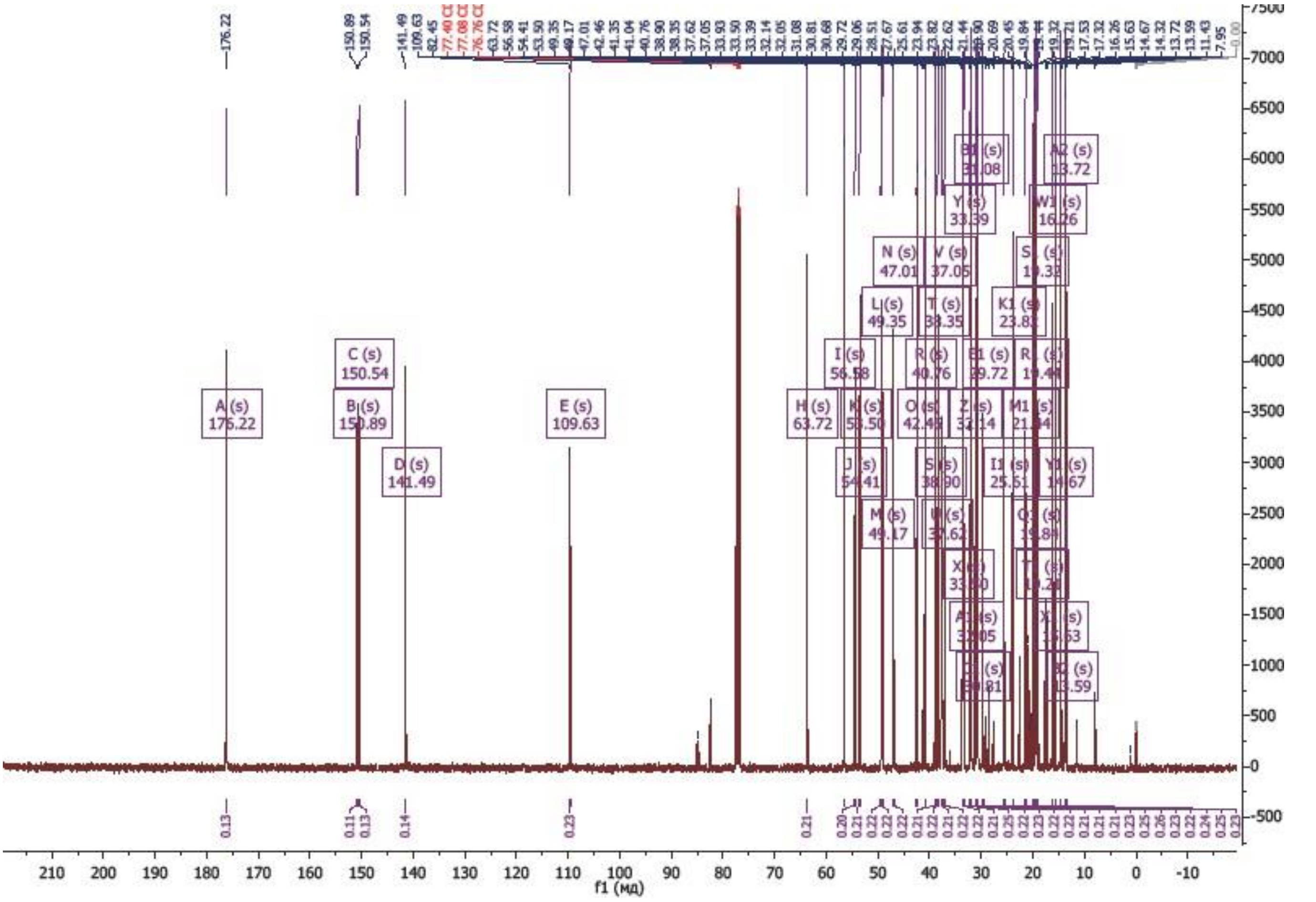

Compound **12**, ^{1}H NMR (400 MHz, $CDCl_3$)

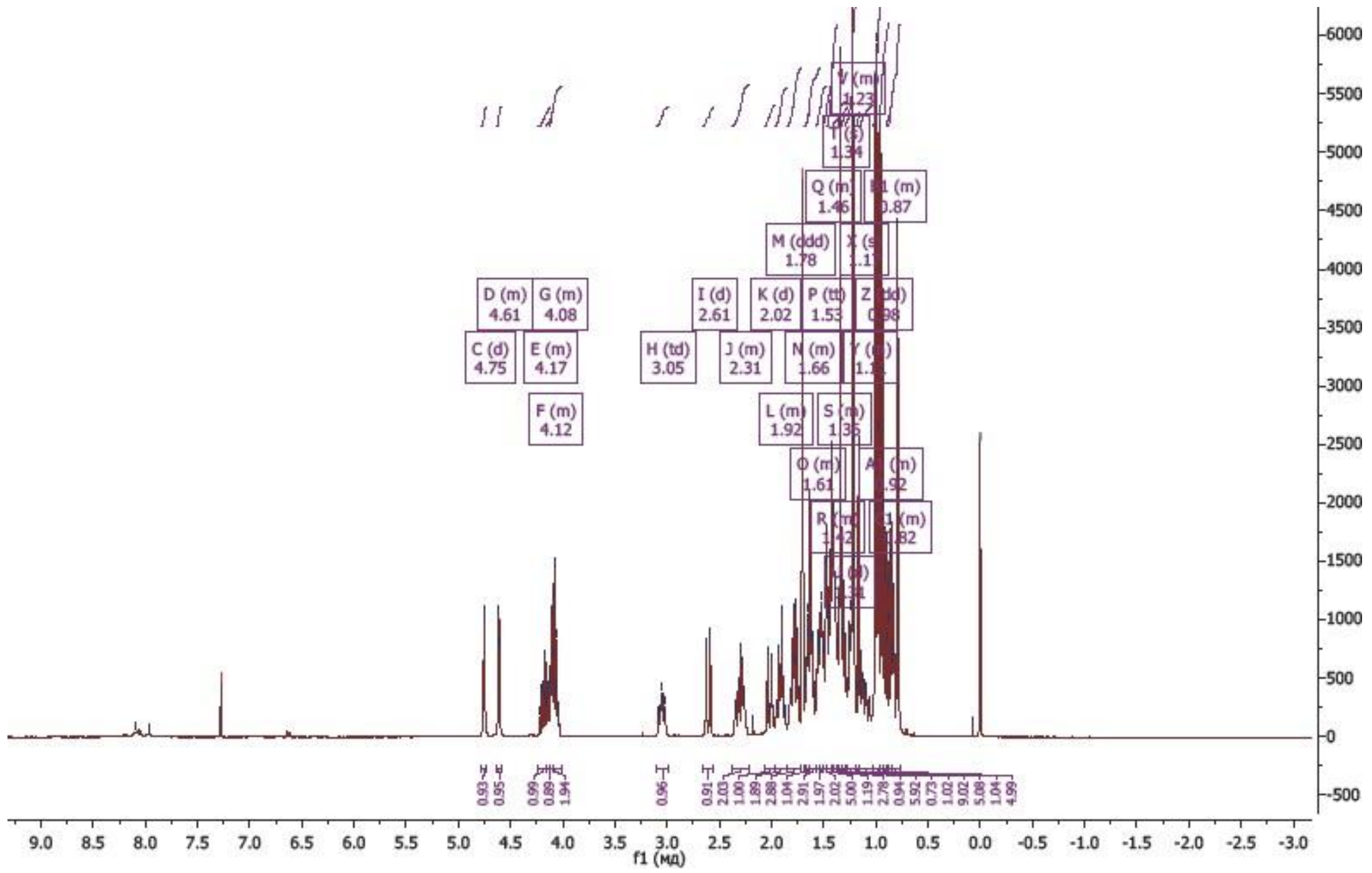

^{13}C NMR (101 MHz, $CDCl_3$)

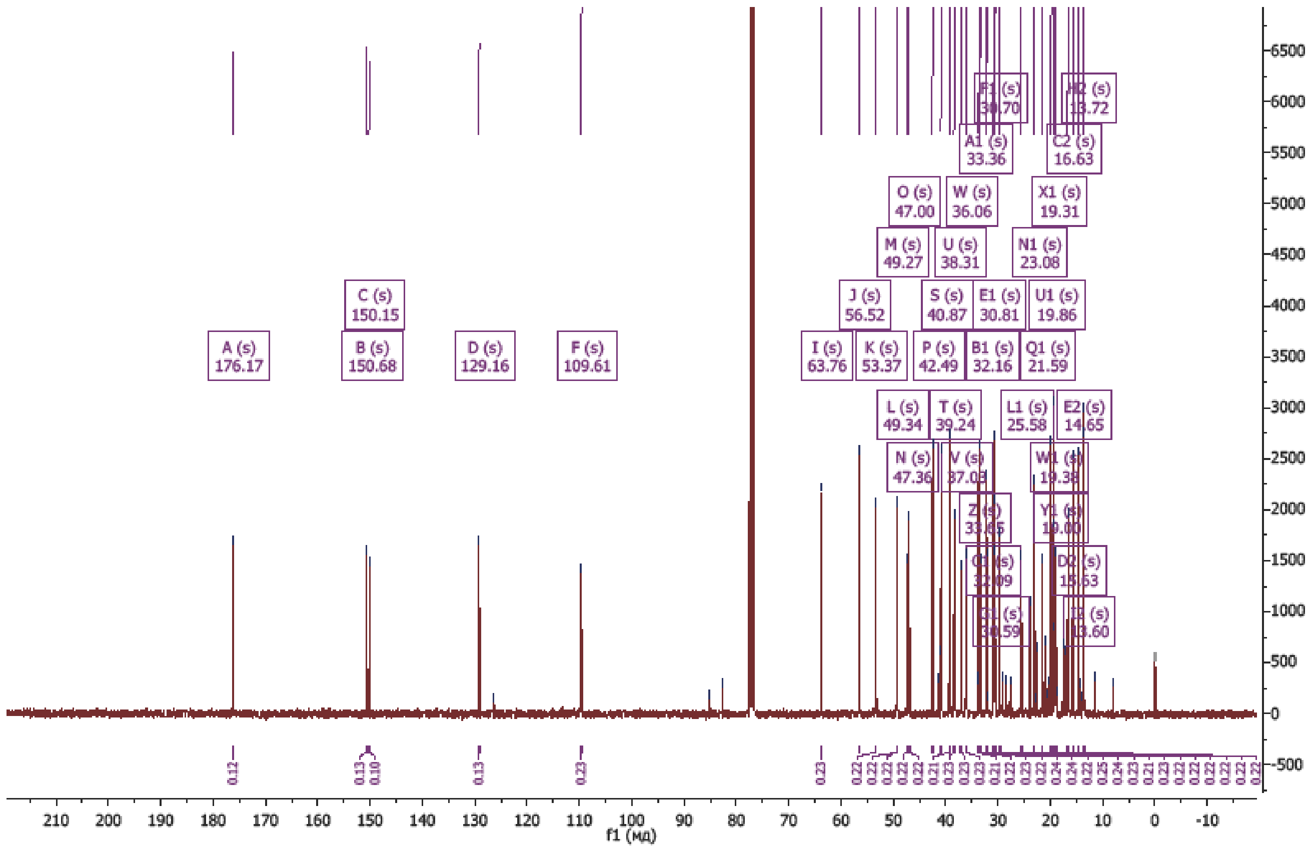

Compound **13**, ^{1}H NMR (400 MHz, $CDCl_3$)

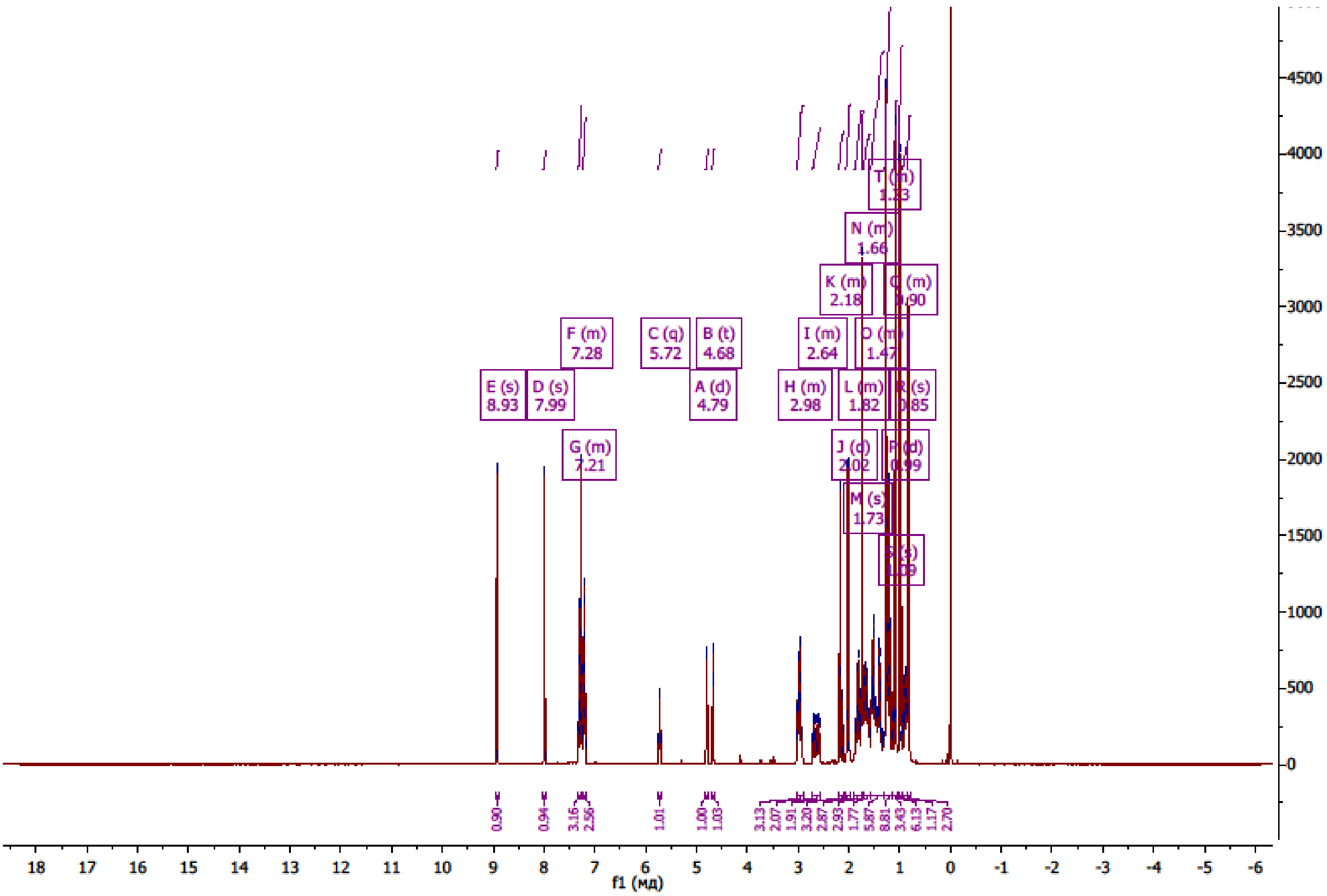

^{13}C NMR (101 MHz, $CDCl_3$)

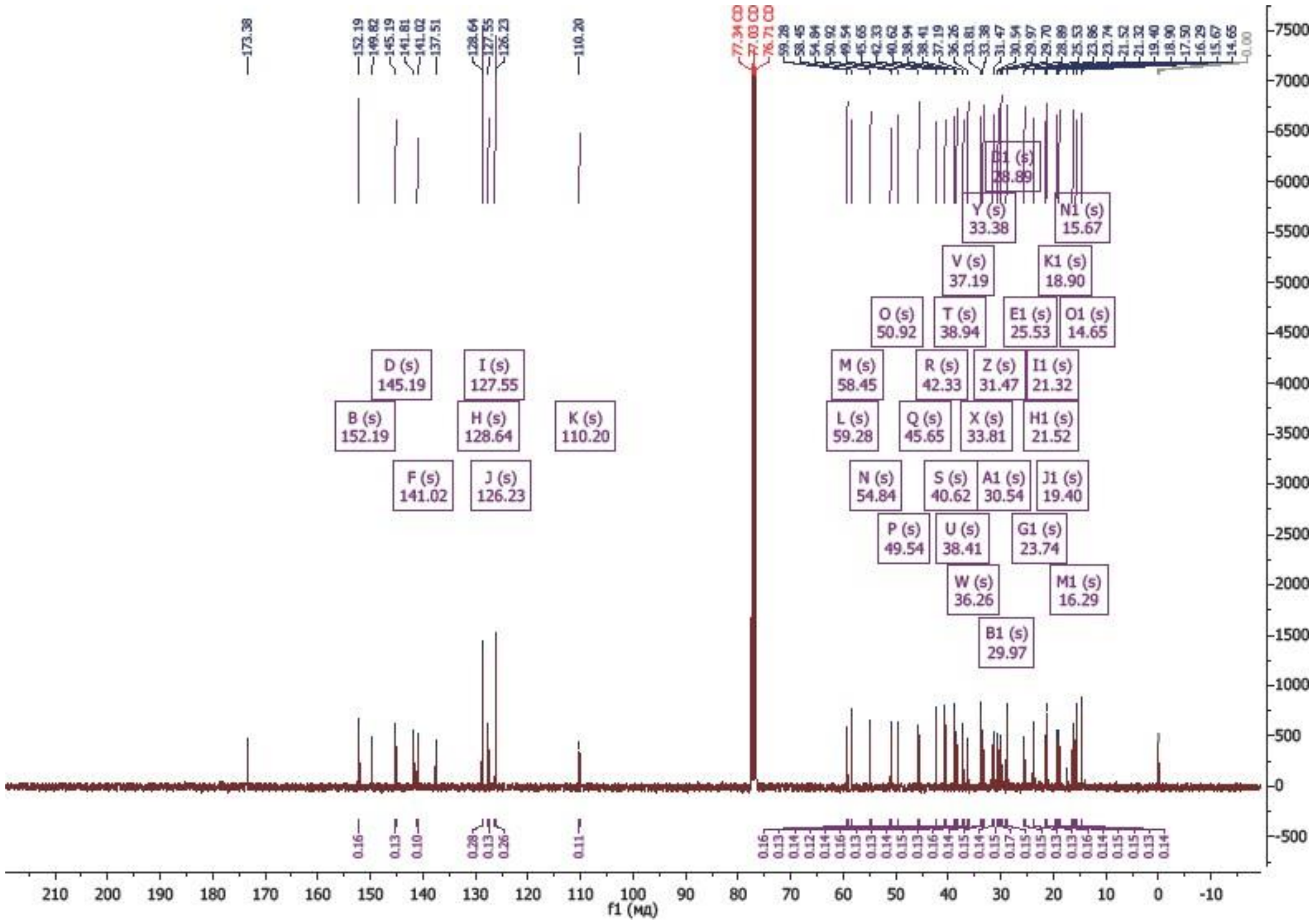

Compound **14**, ^{1}H NMR (400 MHz, $CDCl_3$)

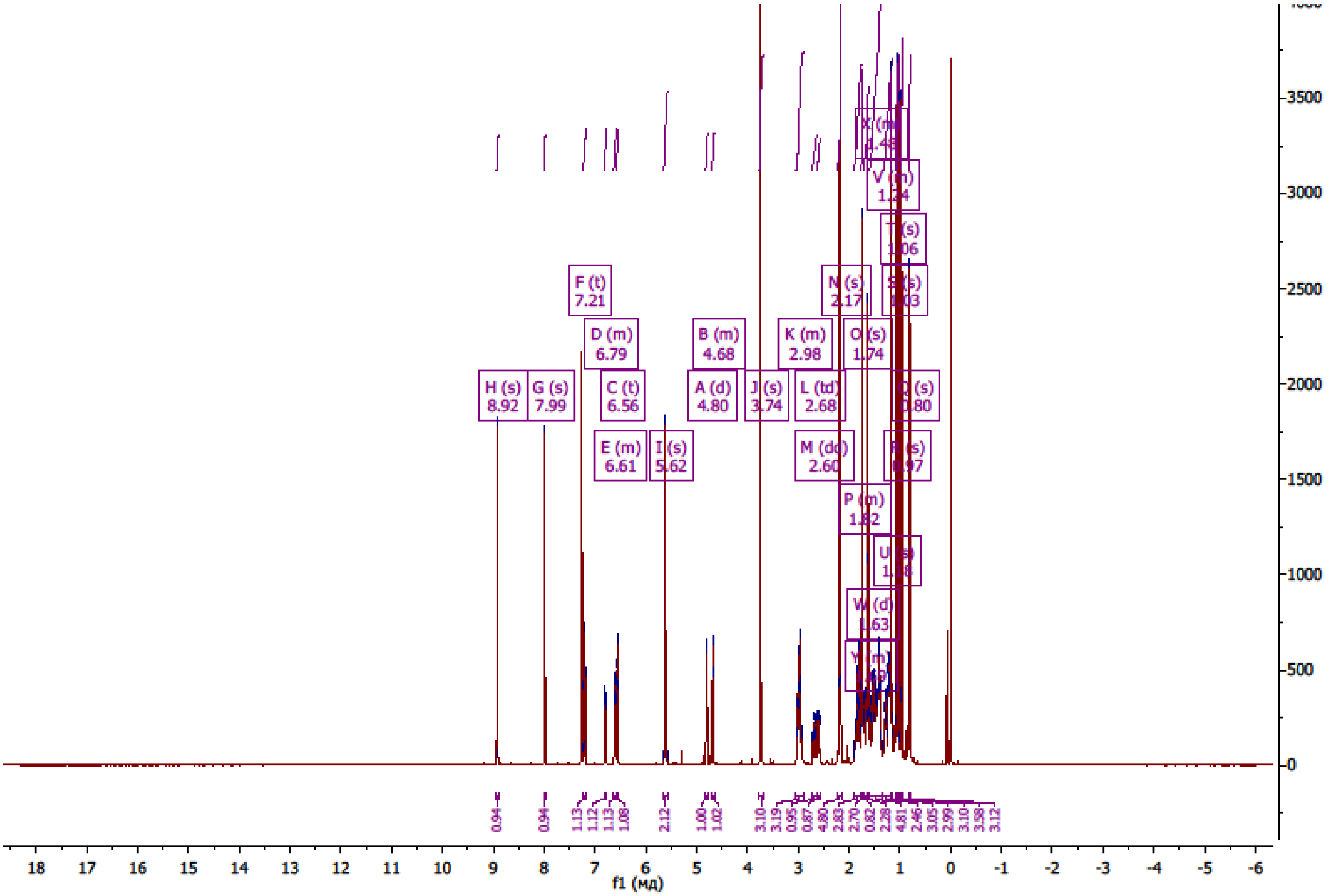

^{13}C NMR (101 MHz, $CDCl_3$)

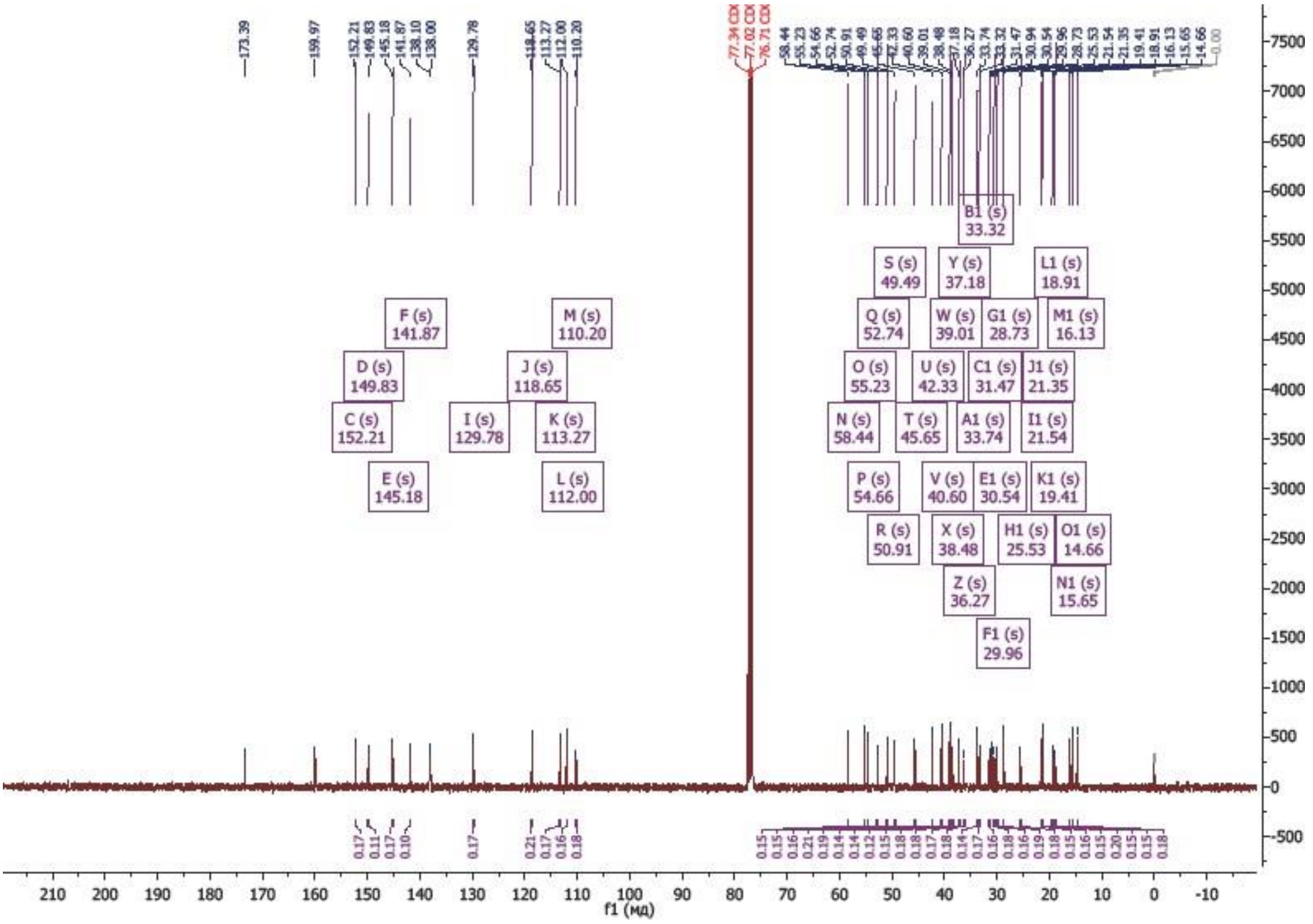

Compound **15**, ^{1}H NMR (400 MHz, $CDCl_3$)

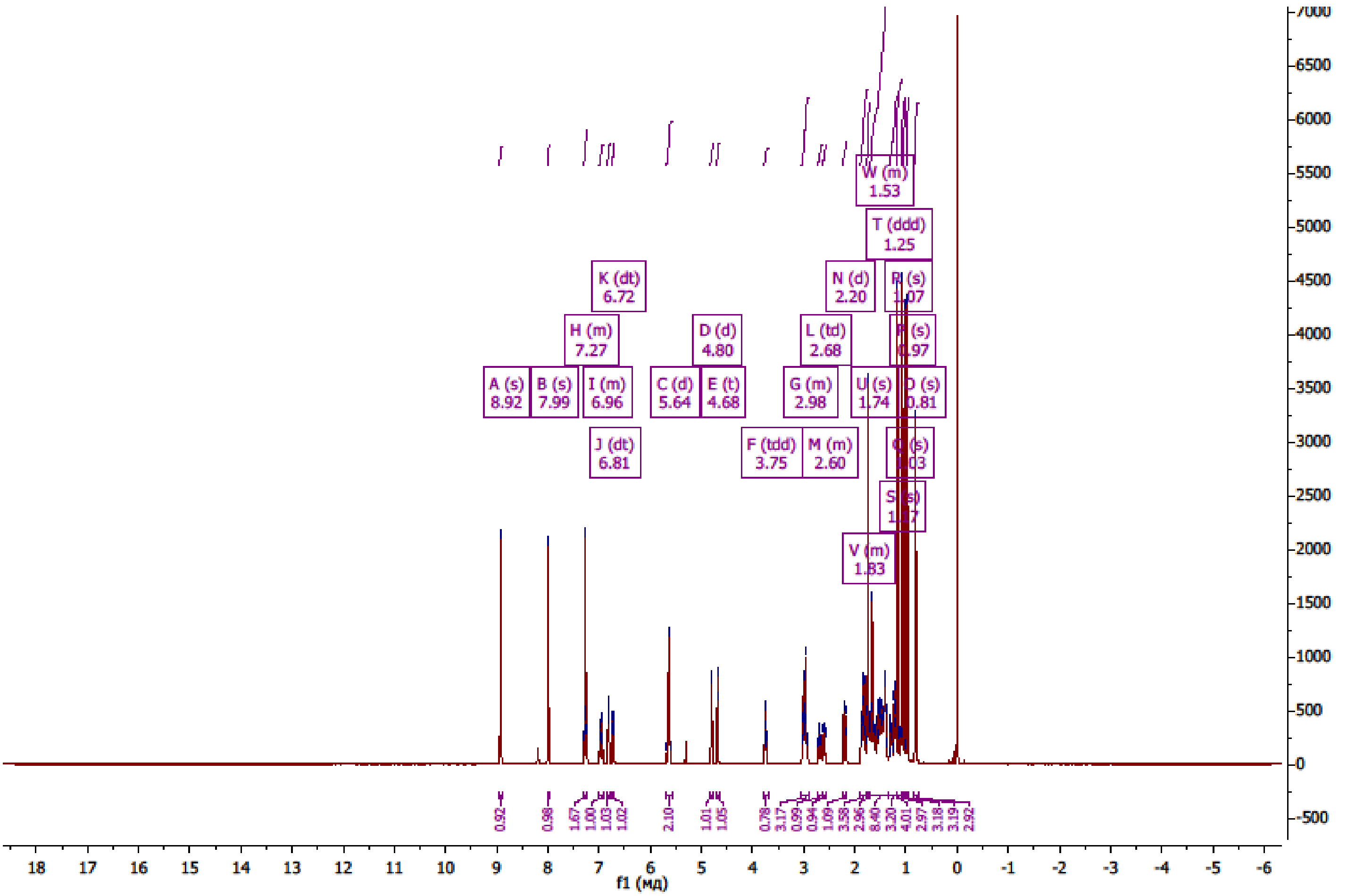

^{13}C NMR (101 MHz, $CDCl_3$)

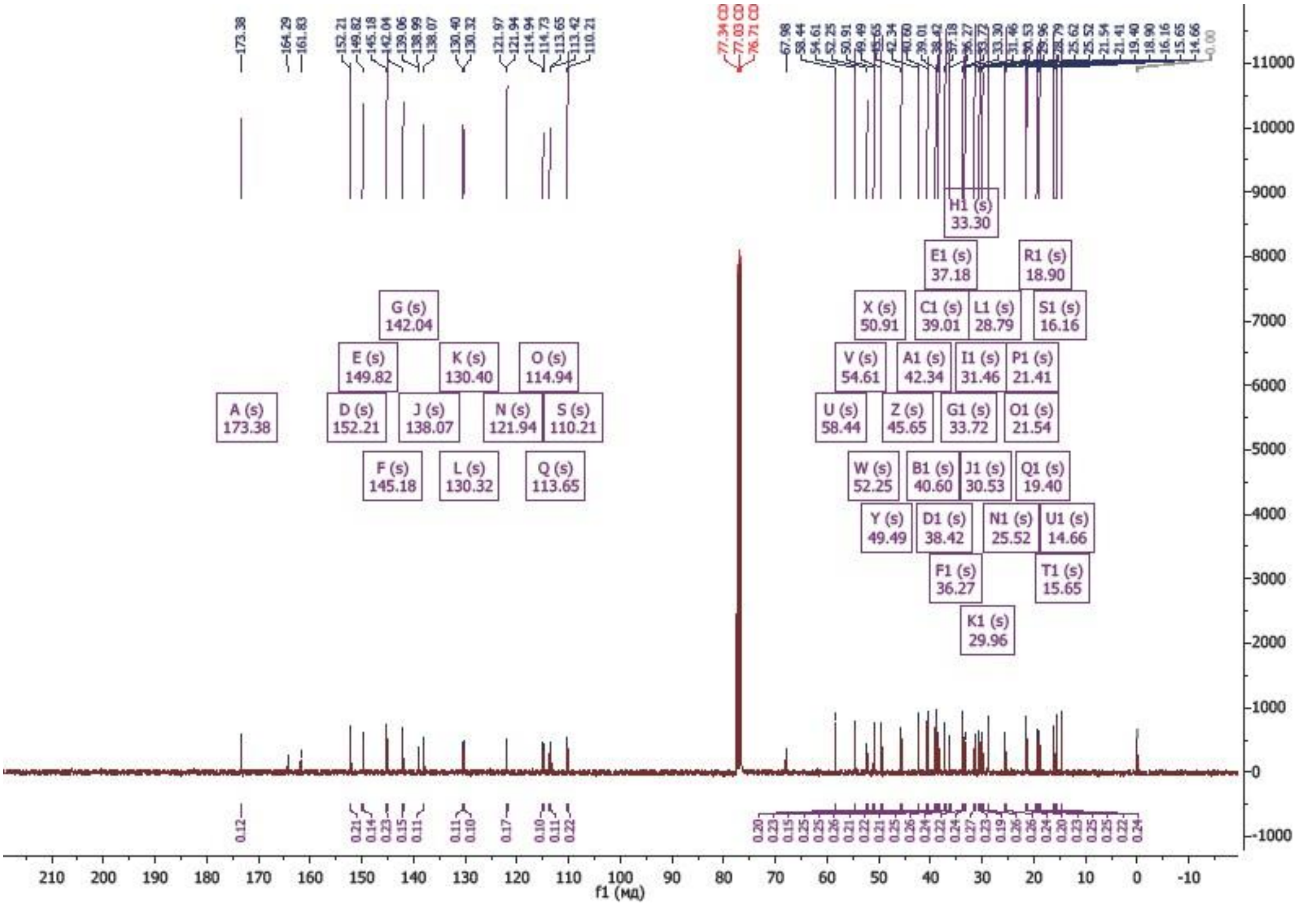

Compound **16**, ^{1}H NMR (400 MHz, $CDCl_3$)

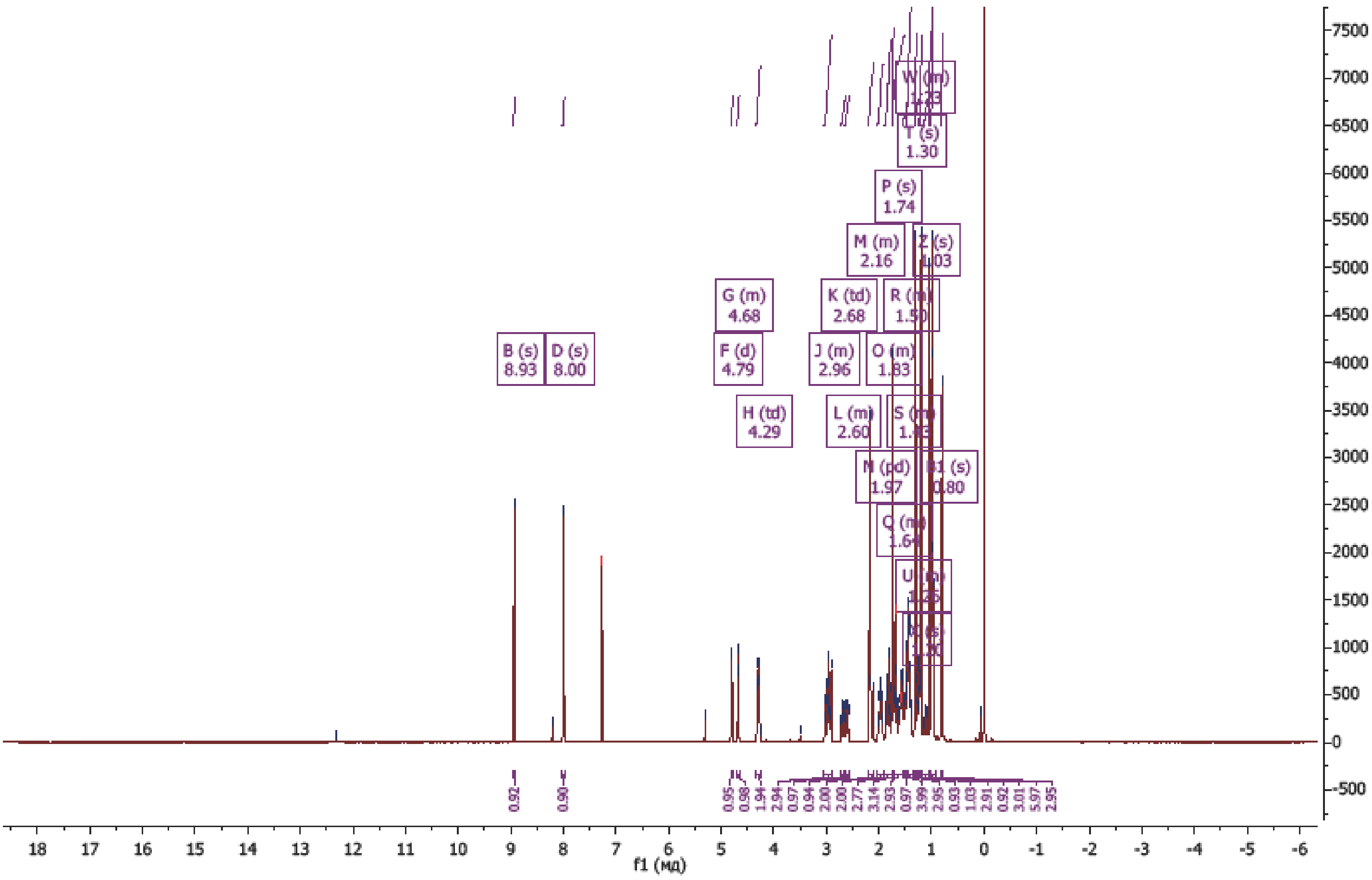

^{13}C NMR (101 MHz, $CDCl_3$)

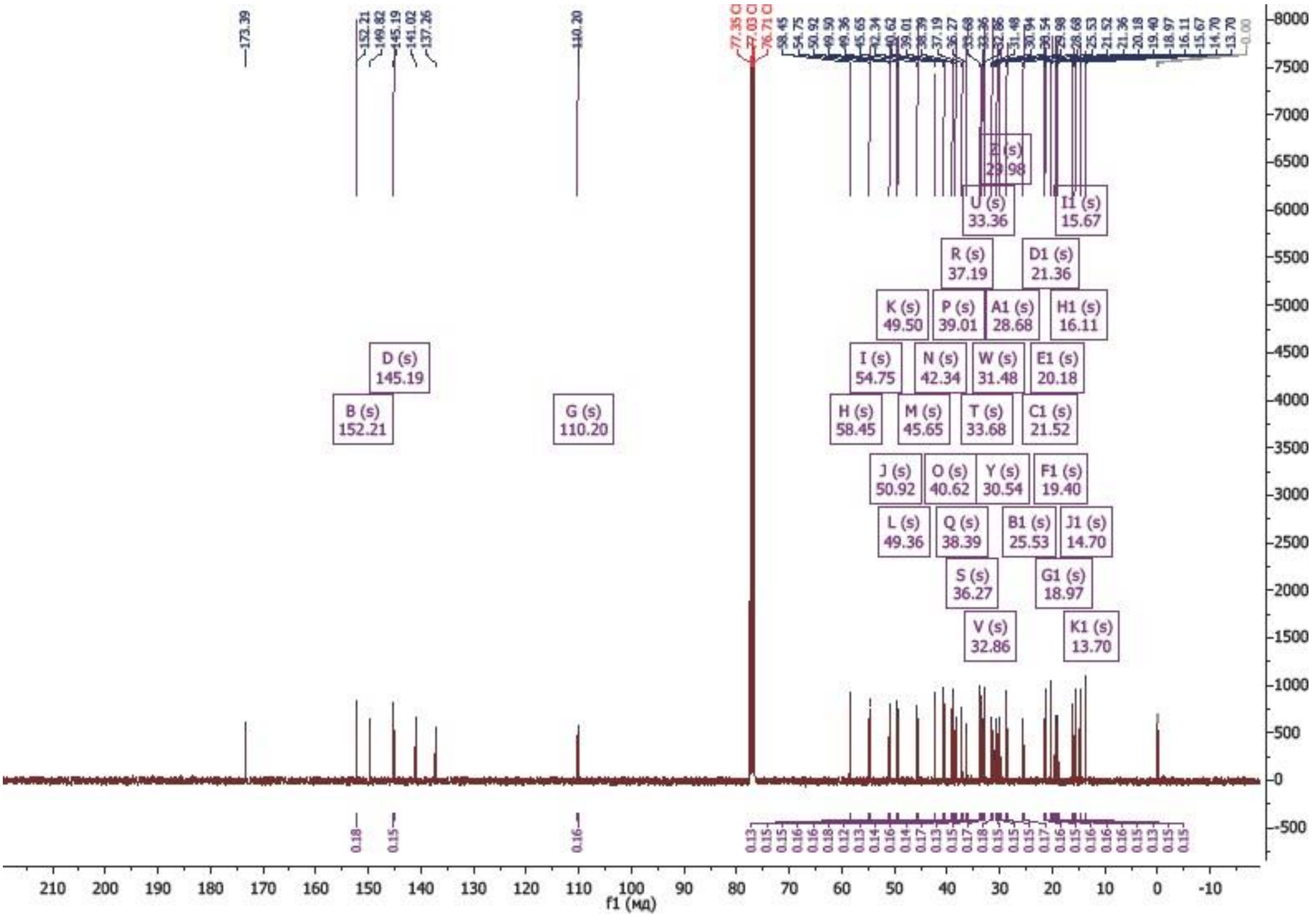

Compound **17**, ^{1}H NMR (400 MHz, $CDCl_3$)

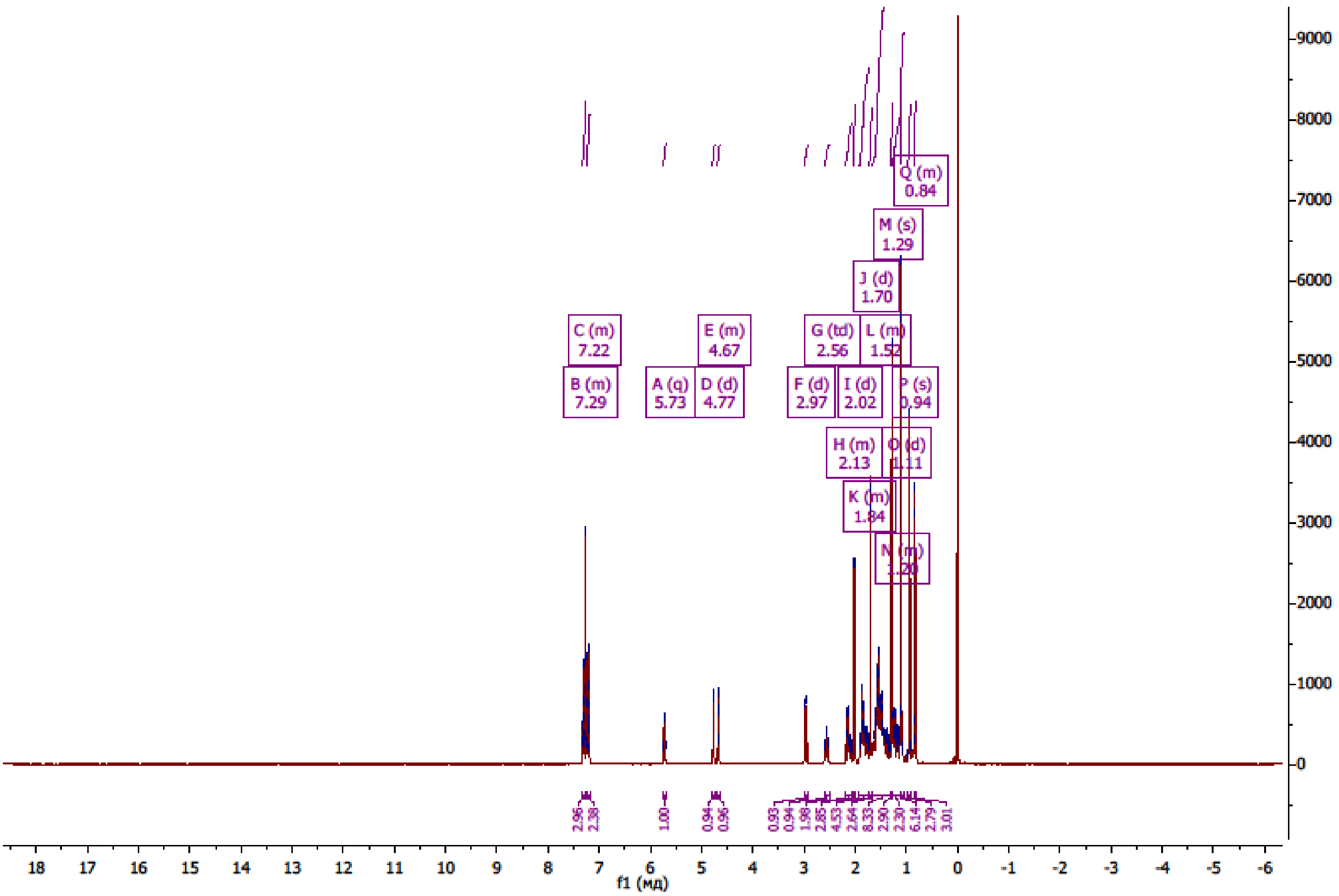

^{13}C NMR (101 MHz, $CDCl_3$)

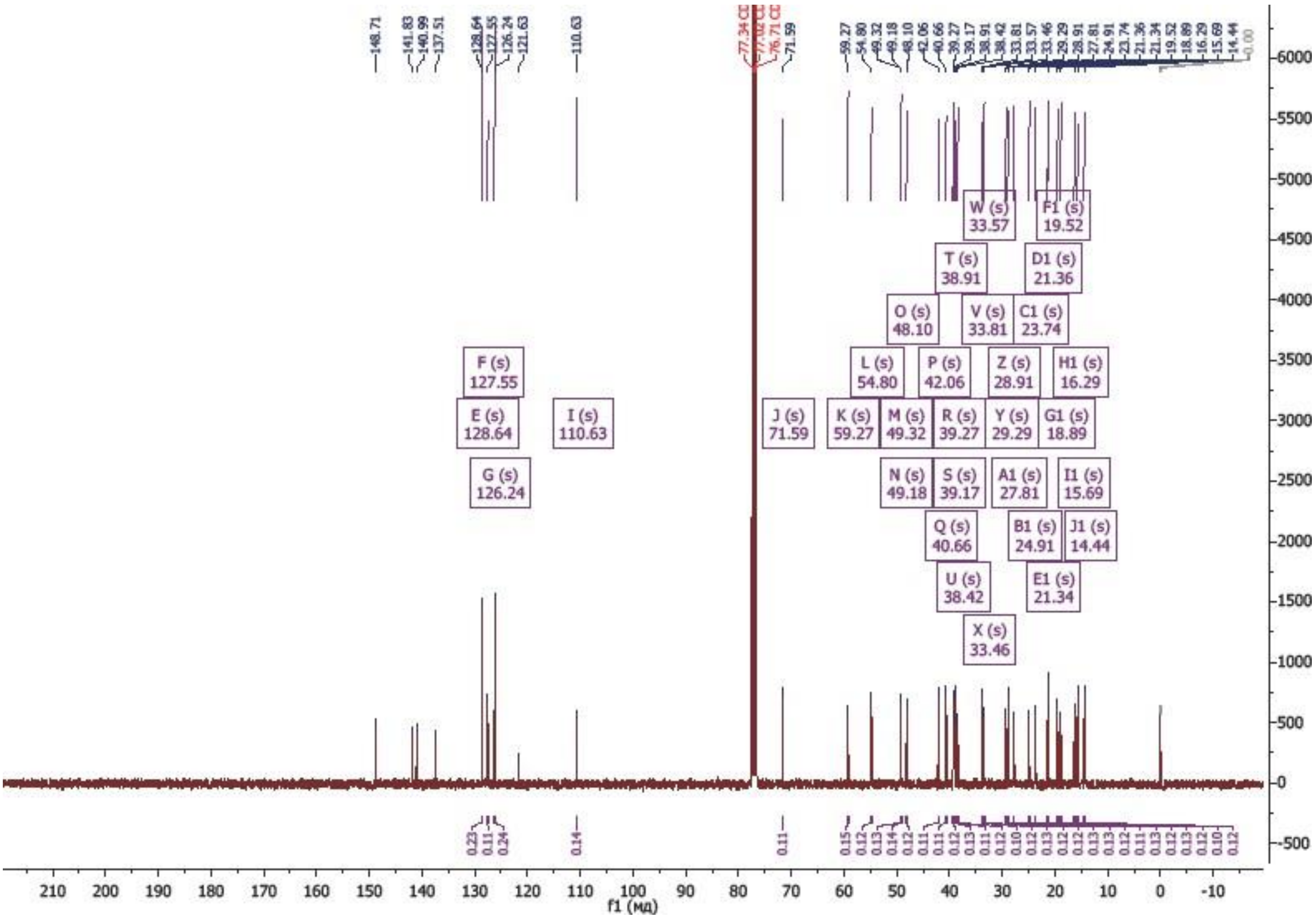

Compound **18**, ^{1}H NMR (400 MHz, $CDCl_3$)

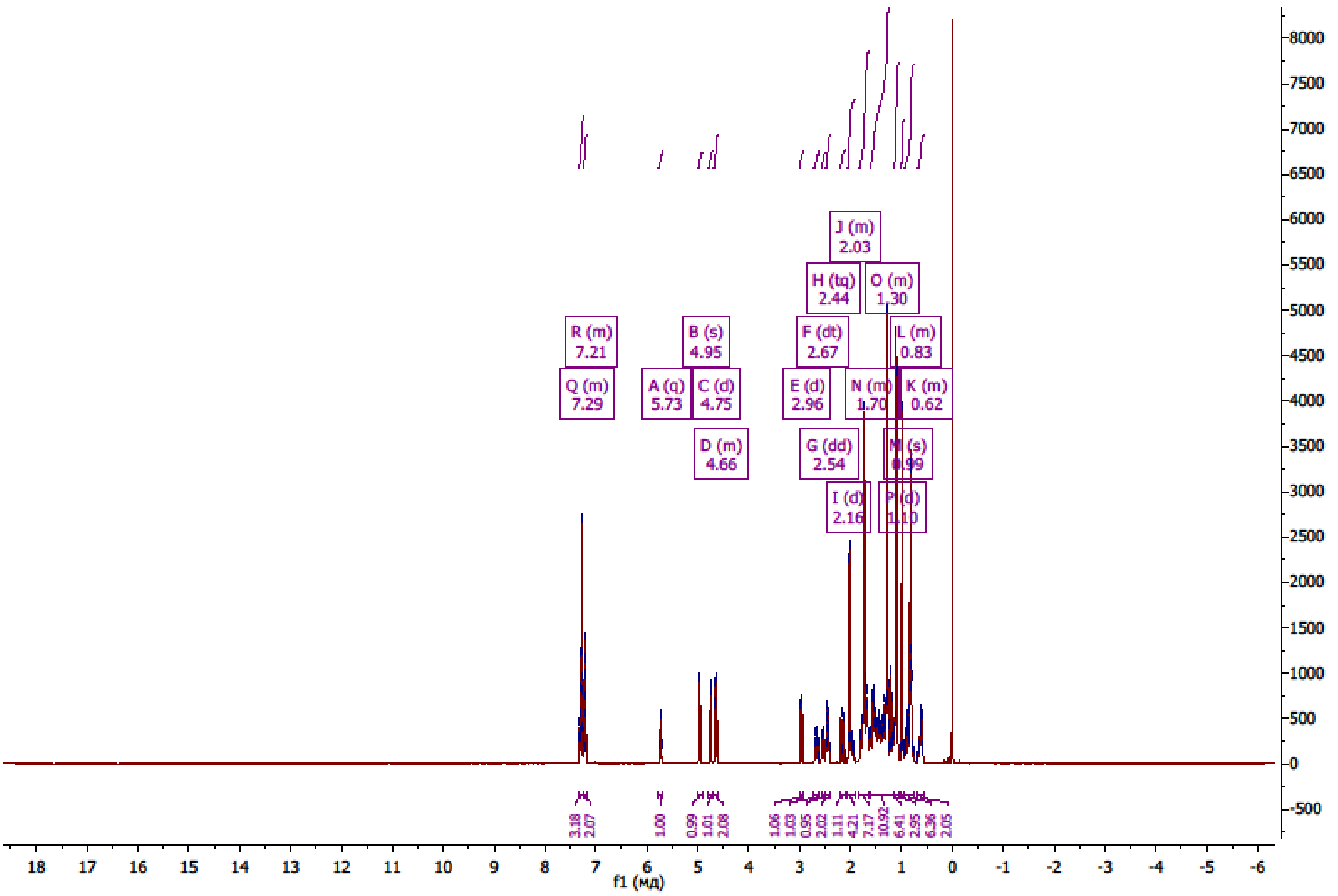

^{13}C NMR (101 MHz, $CDCl_3$)

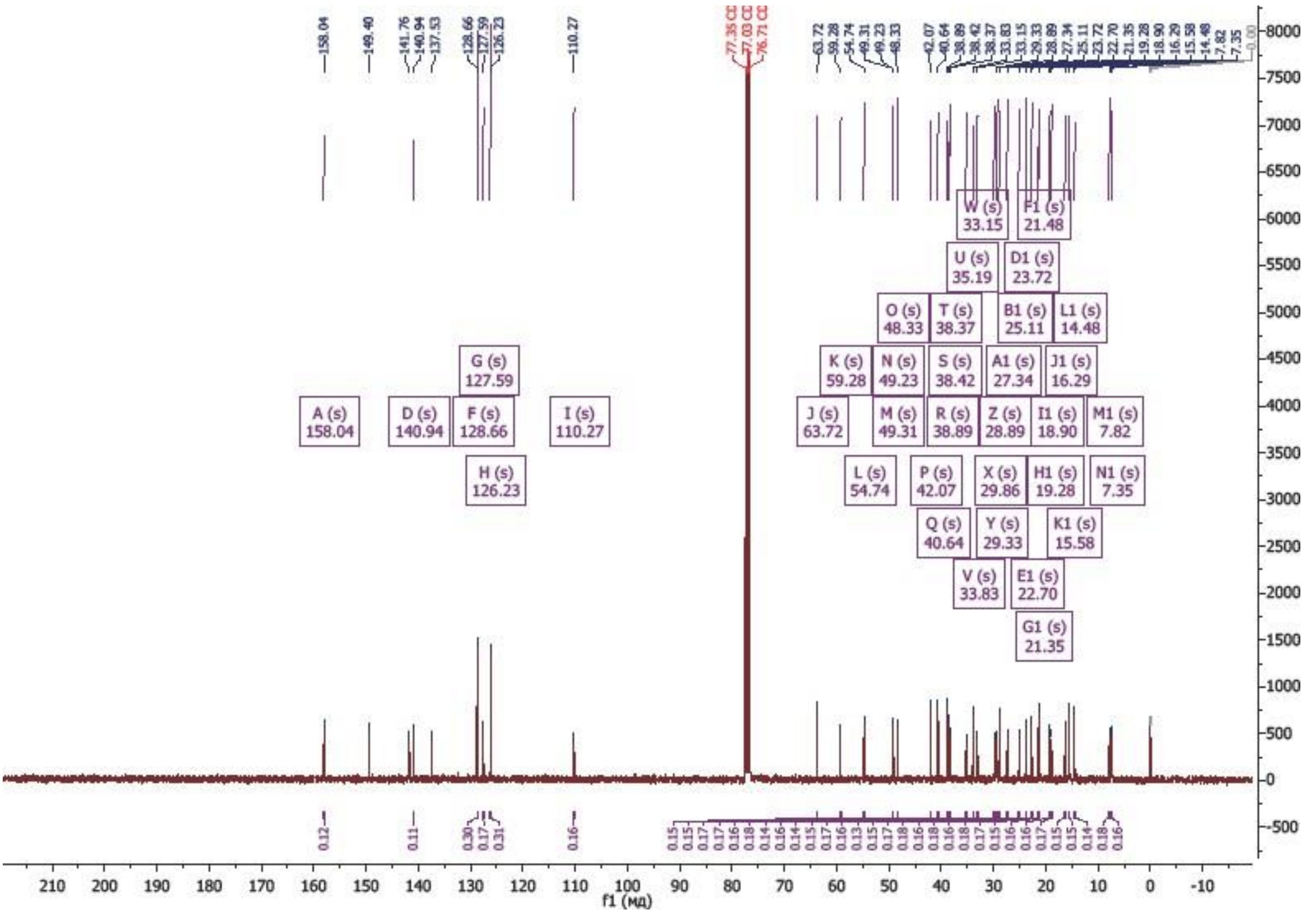

Compound **19**, ^{1}H NMR (400 MHz, $CDCl_3$)

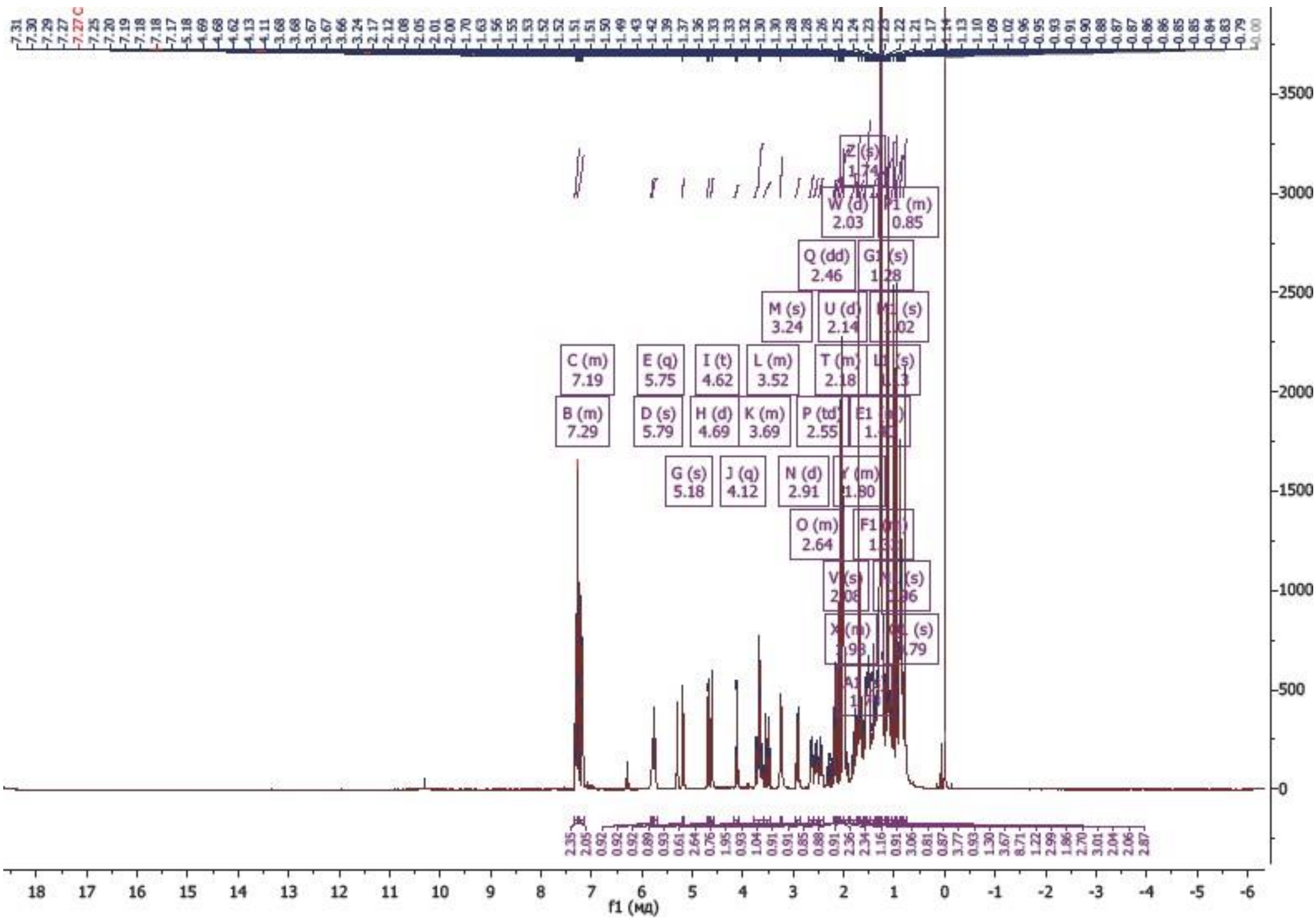

^{13}C NMR (101 MHz, $CDCl_3$)

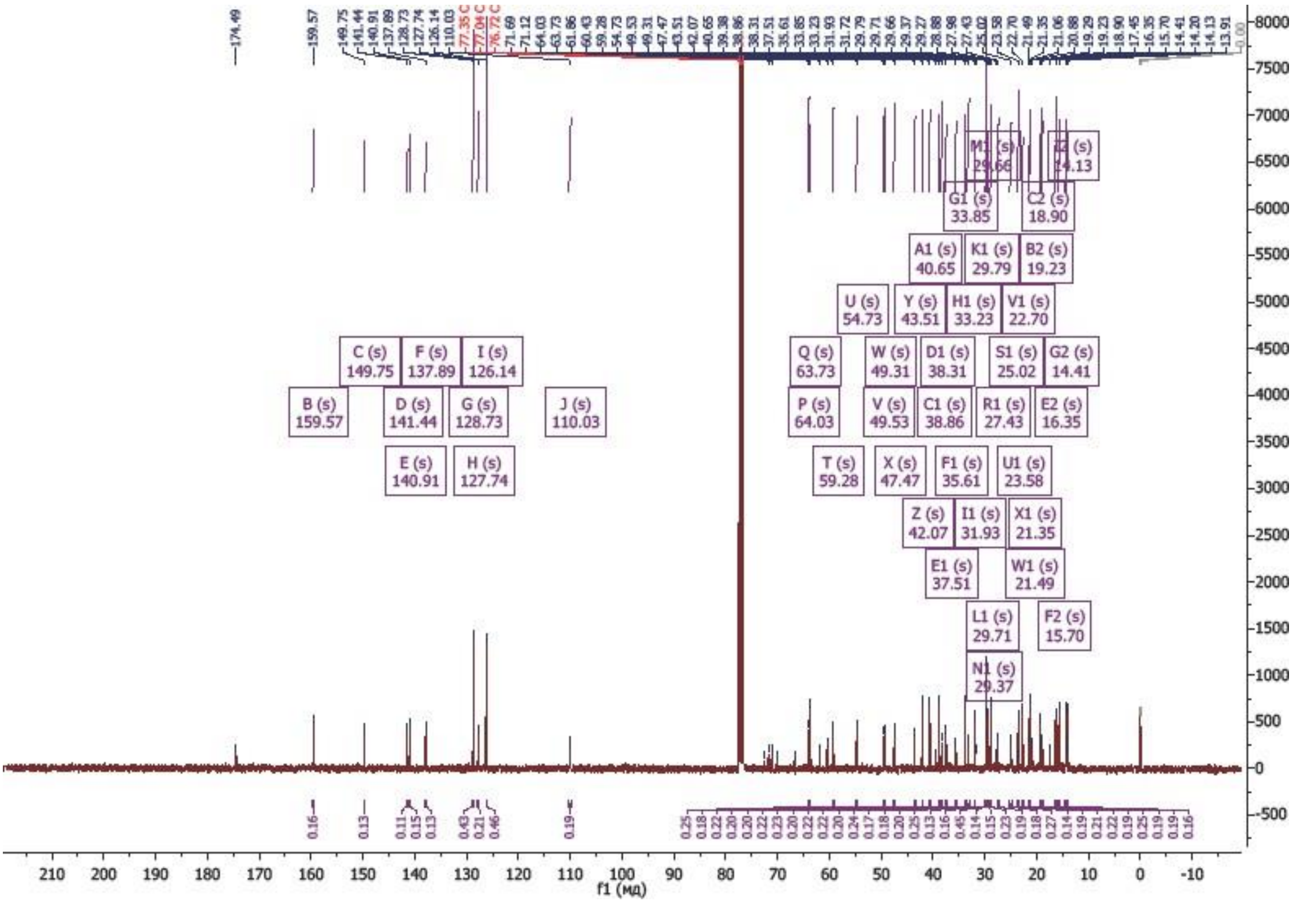

Compound **20**, ^{1}H NMR (400 MHz, $CDCl_3$)

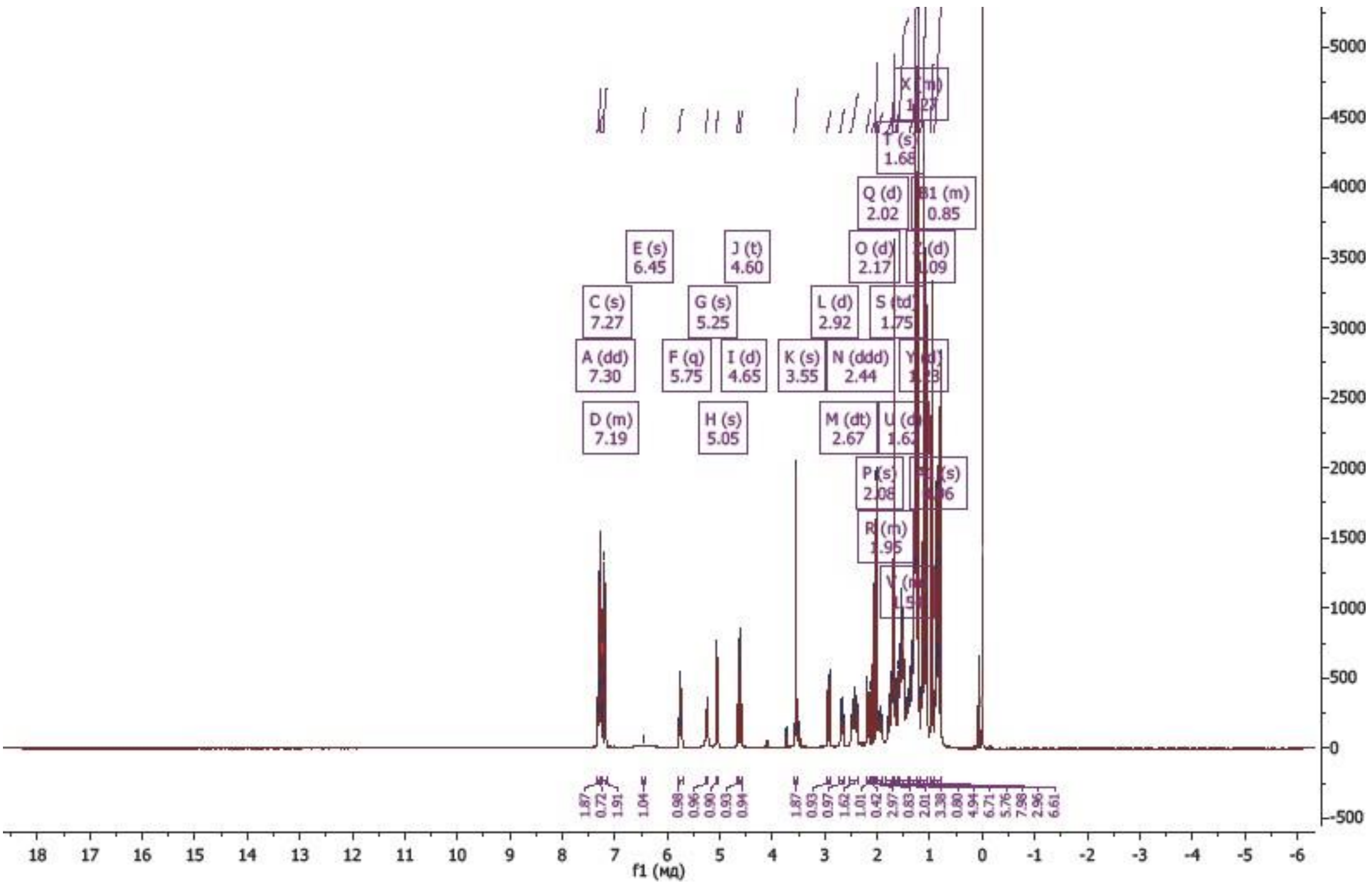

^{13}C NMR (101 MHz, $CDCl_3$)

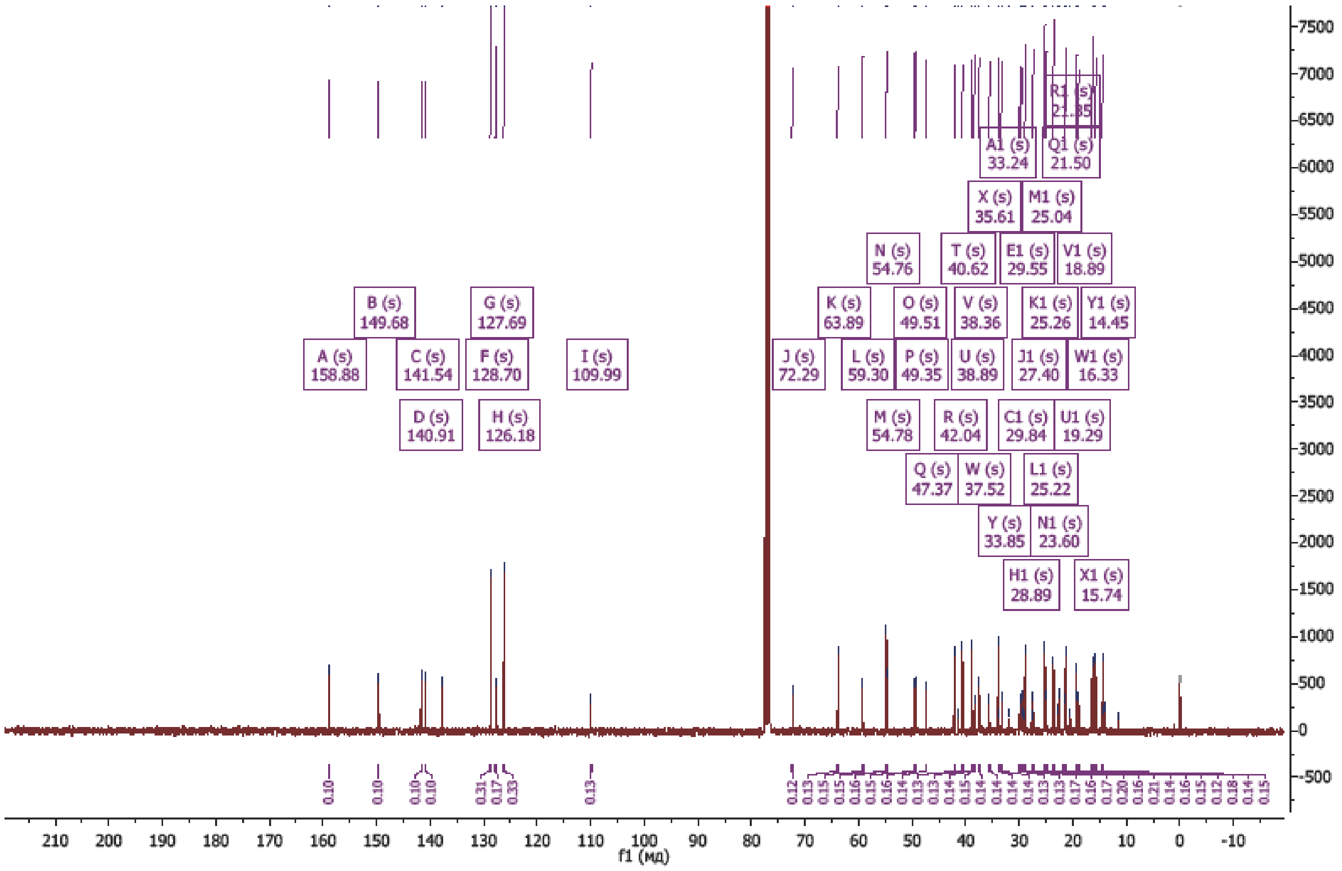

Compound **21**, ^{1}H NMR (400 MHz, $CDCl_3$)

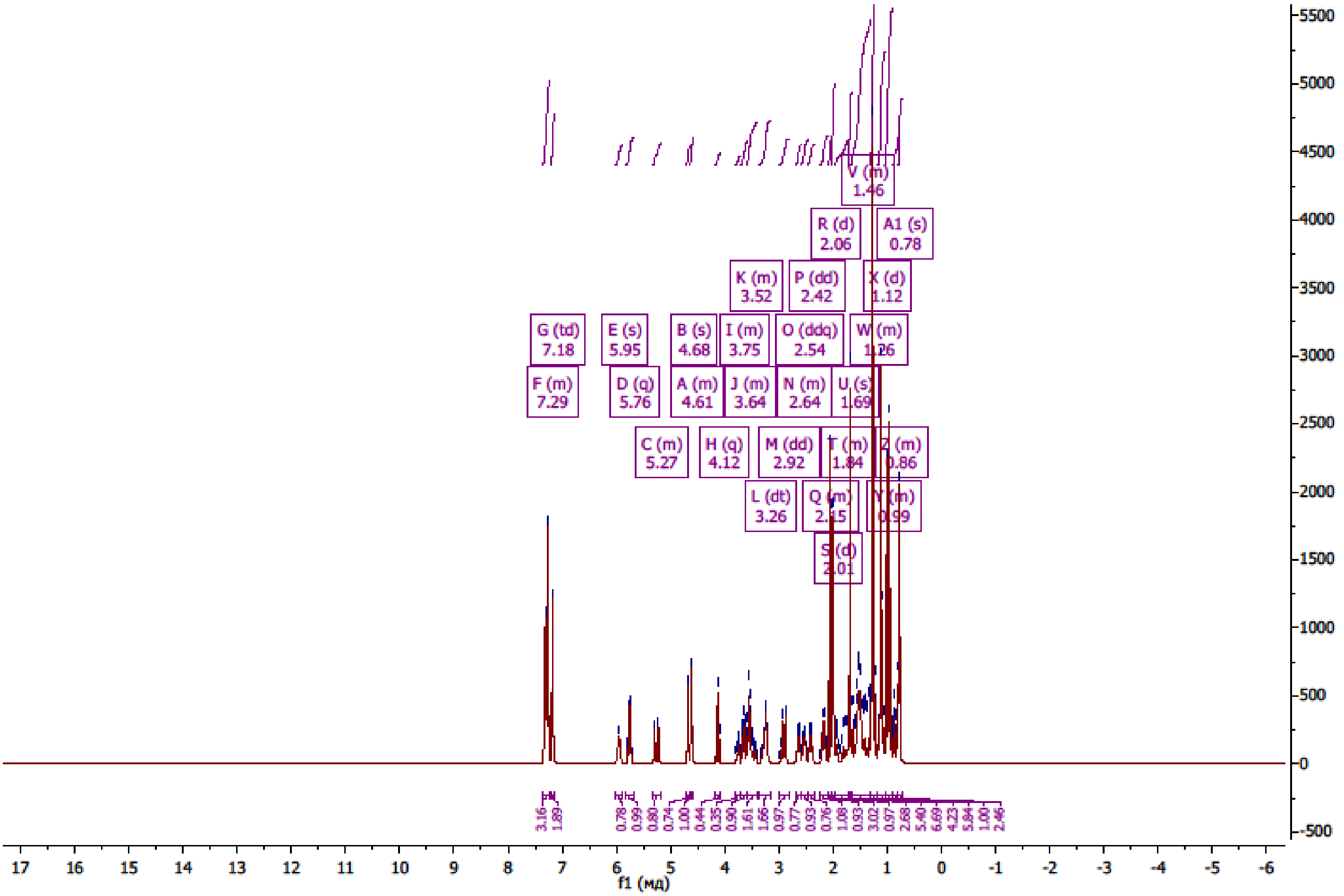

^{13}C NMR (101 MHz, $CDCl_3$)

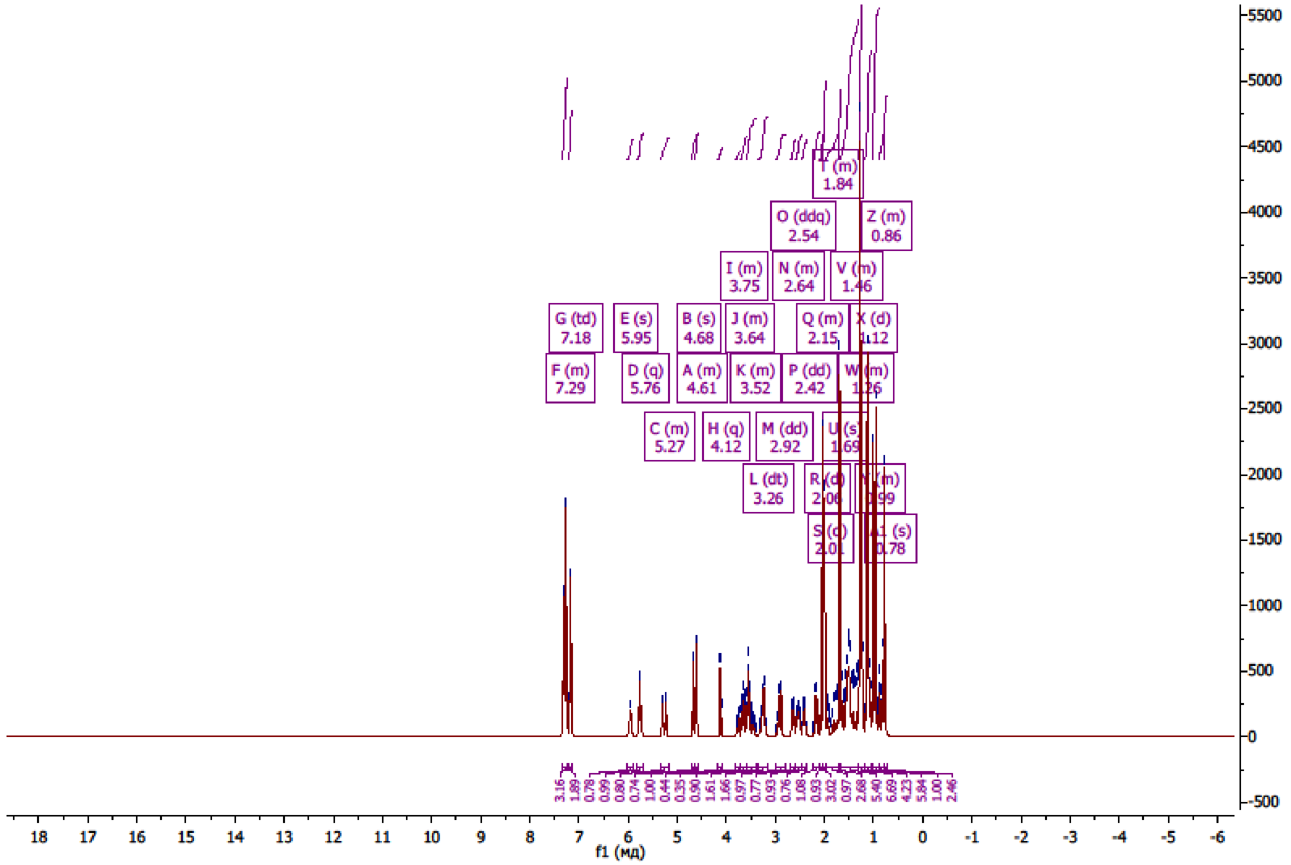

Compound **22**, ^{1}H NMR (400 MHz, $CDCl_3$)

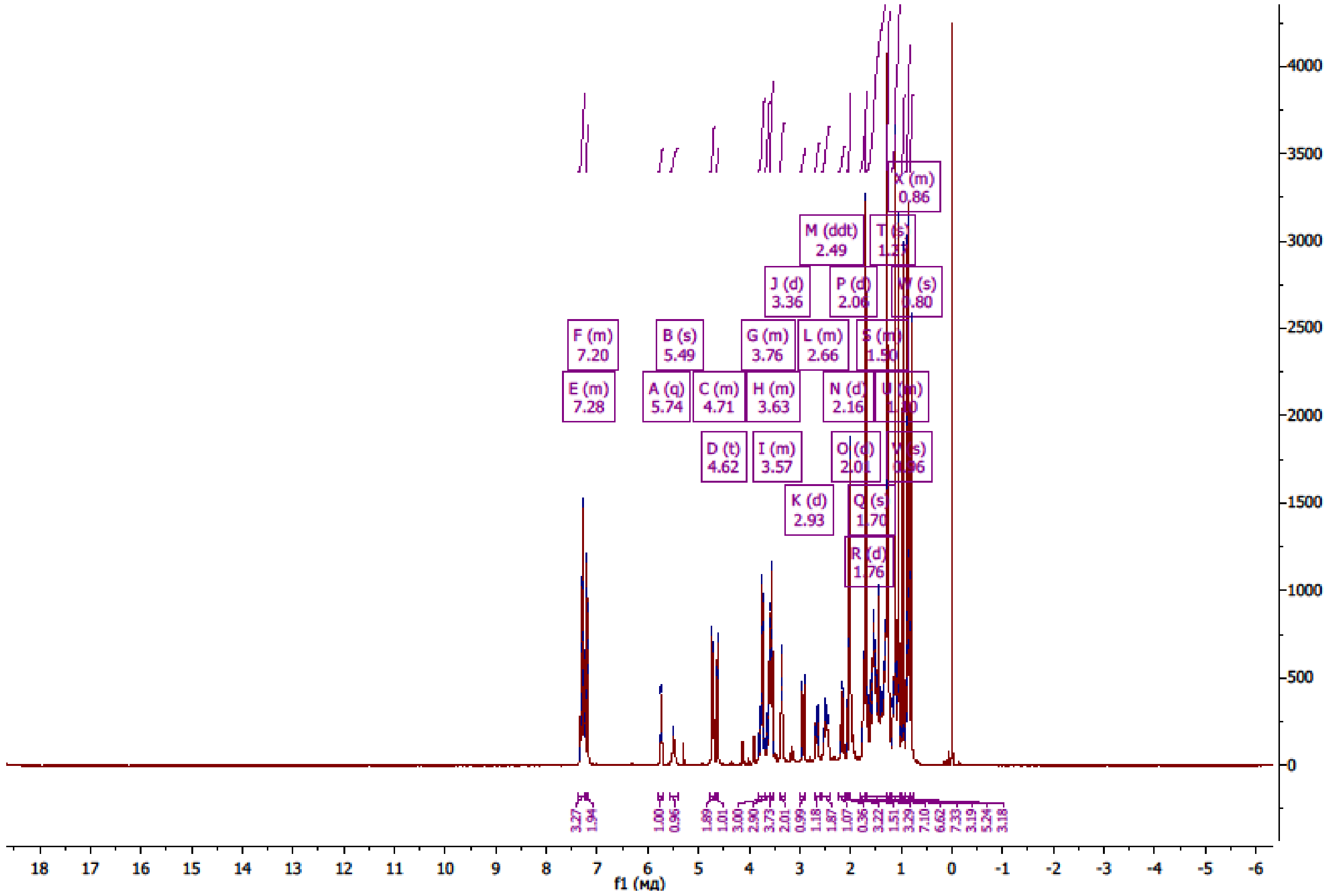

^{13}C NMR (101 MHz, $CDCl_3$)

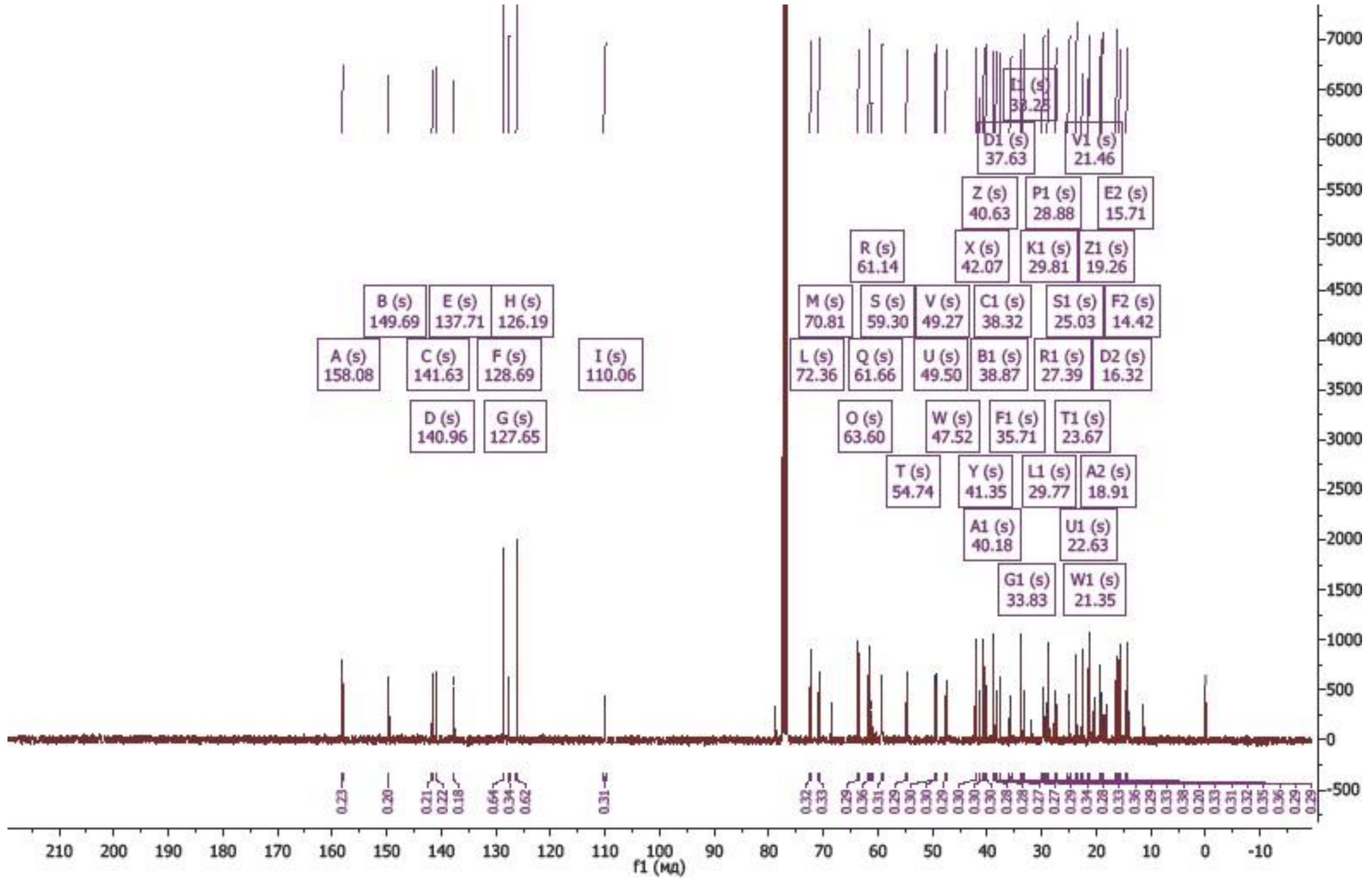

Compound **4** (MS)

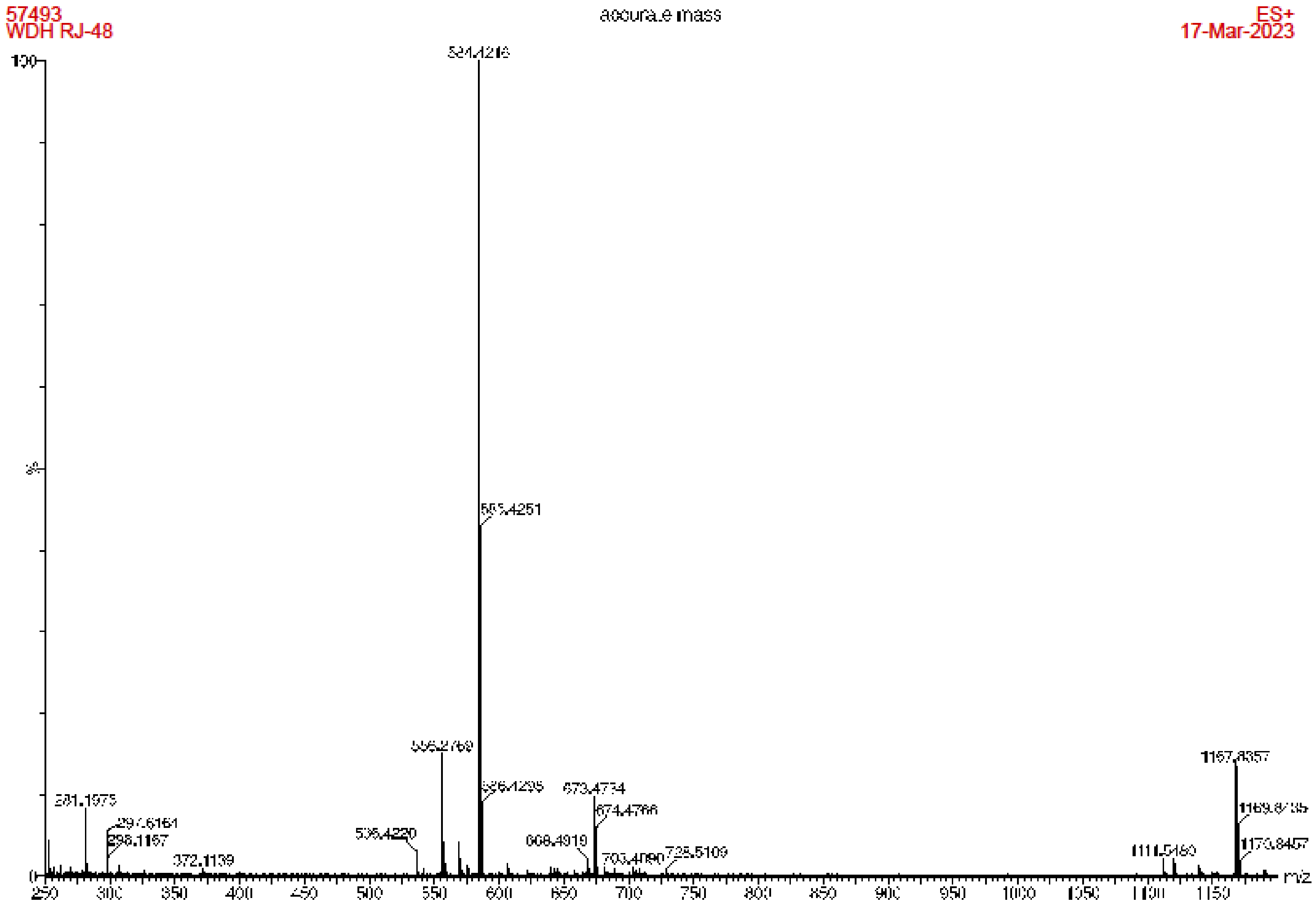

Compound **5** (MS)

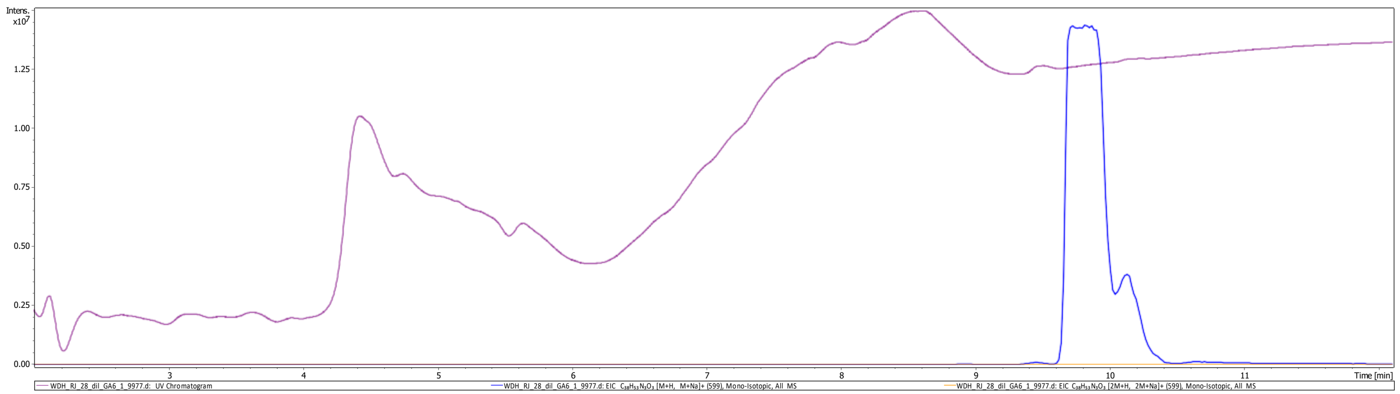

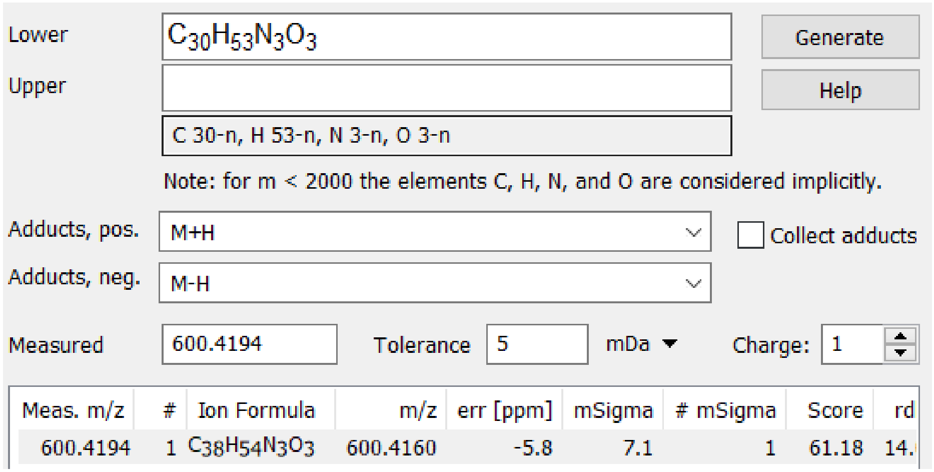

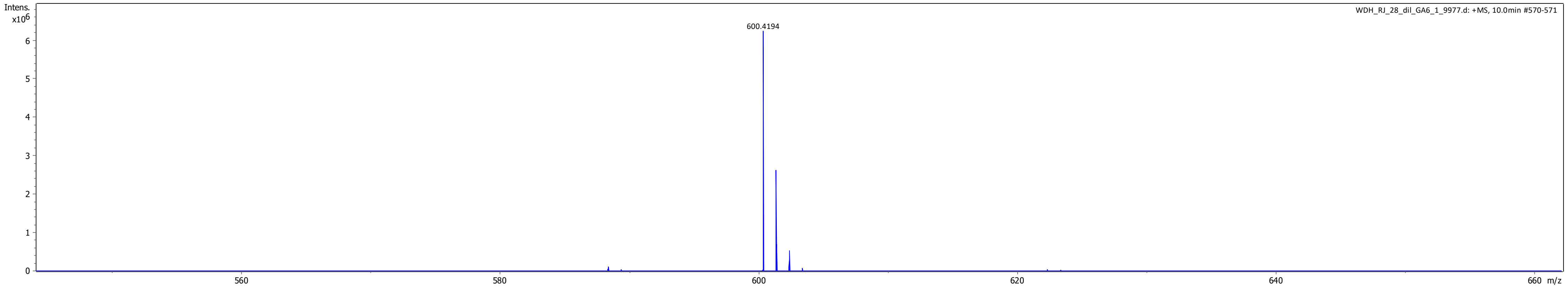

Compound **6** (MS)

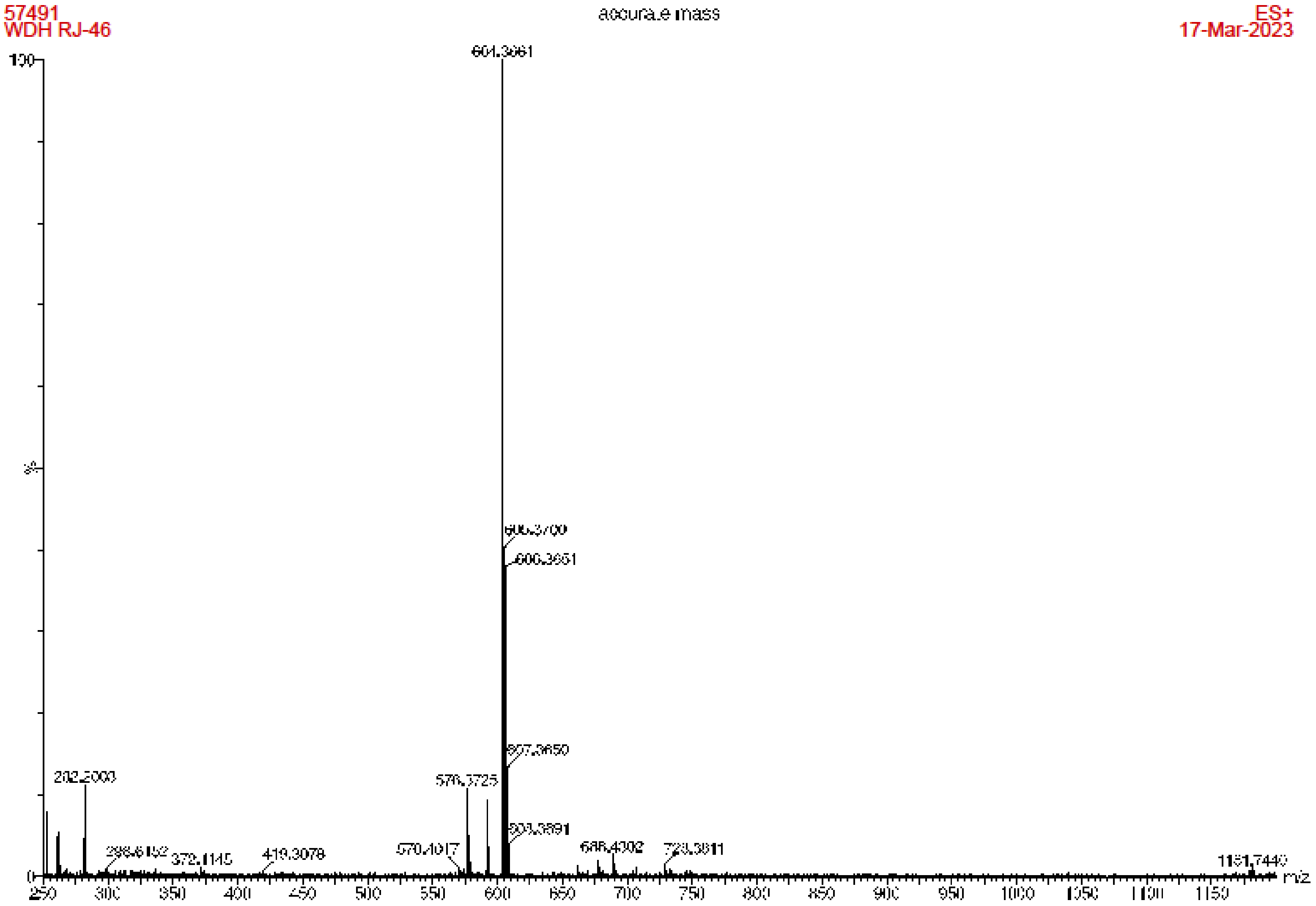

Compound **7** (MS)

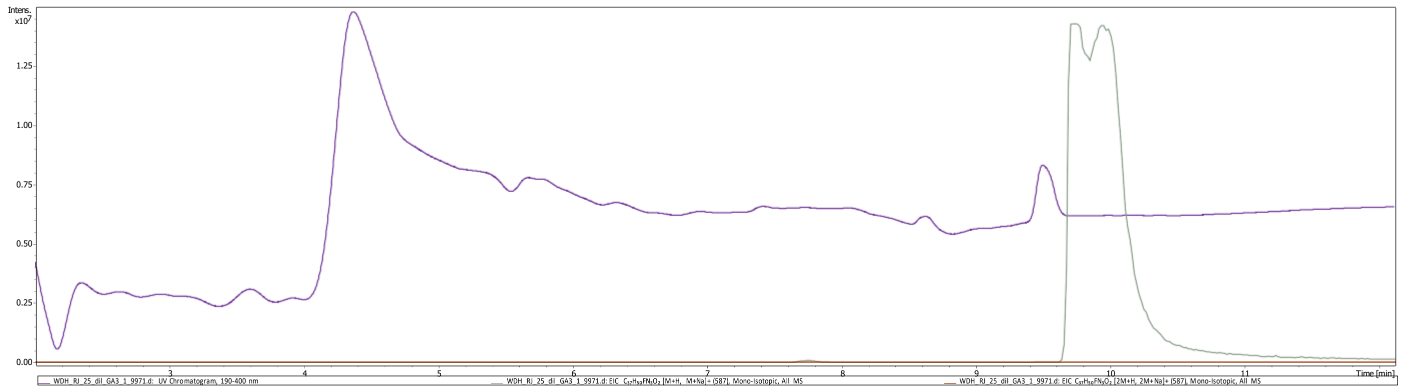

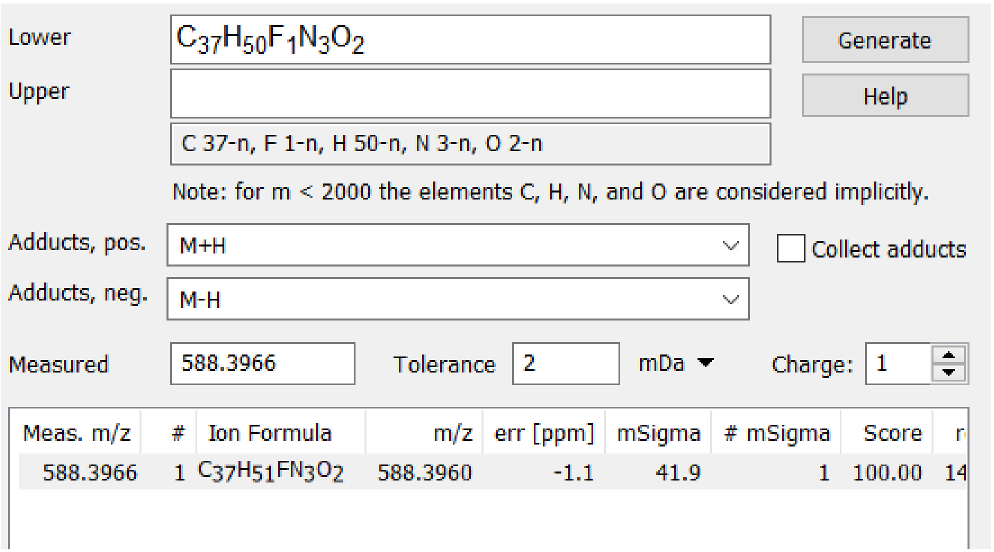

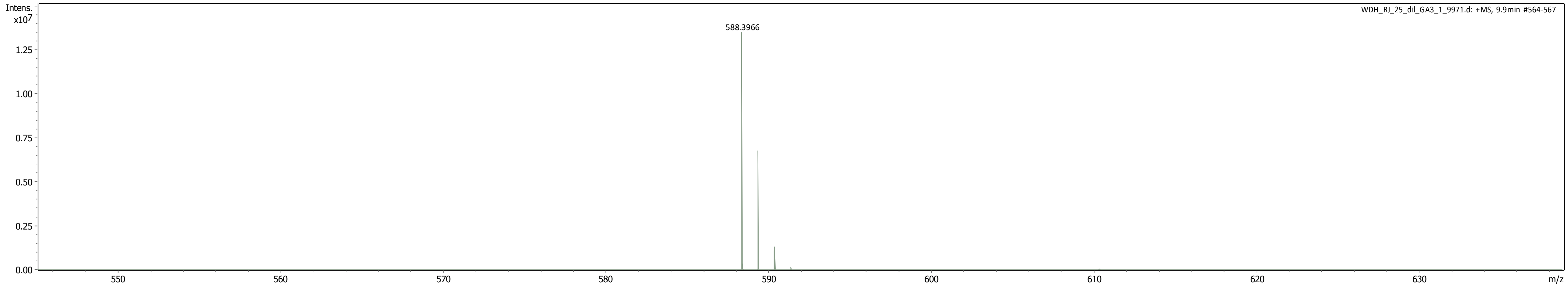

Compound **8** (MS)

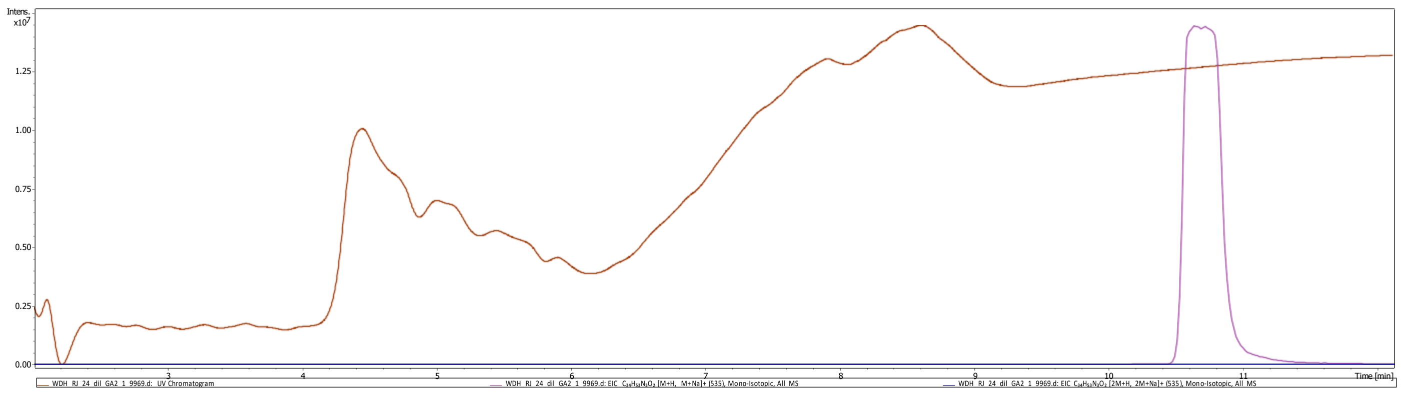

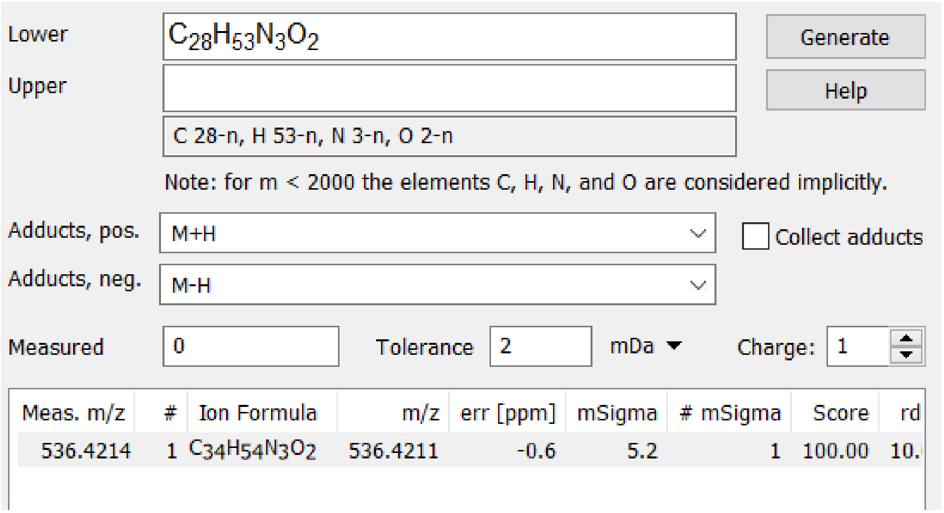
Lower: $C_{28}H_{53}N_3O_2$

Upper:

C 28-n, H 53-n, N 3-n, O 2-n

Note: for m < 2000 the elements C, H, N, and O are considered implicitly.

Adducts, pos.: M+H

Adducts, neg.: M-H

Collect adducts

Measured: 0 Tolerance: 2 mDa Charge: 1

Generate

Help

Meas. m/z	#	Ion Formula	m/z	err [ppm]	mSigma	# mSigma	Score	rd
536.4214	1	$C_{34}H_{54}N_3O_2$	536.4211	-0.6	5.2	1	100.00	10.

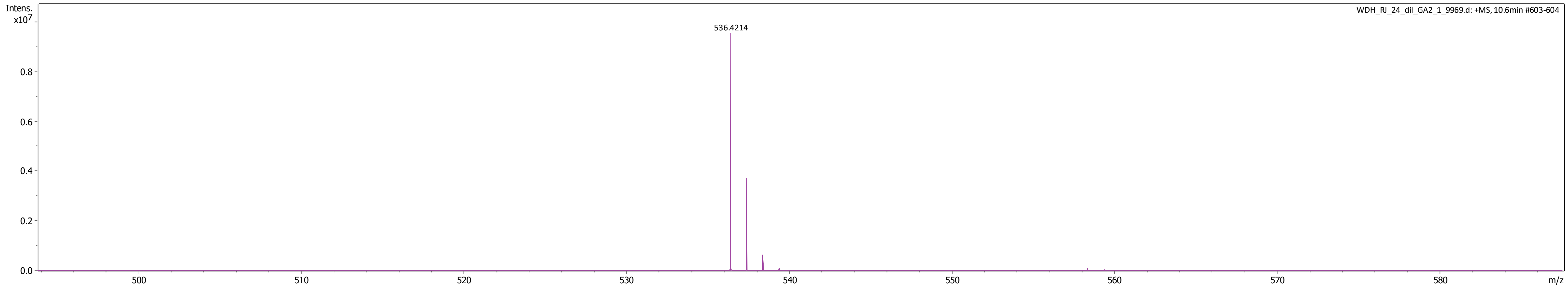

Compound **9** (MS)

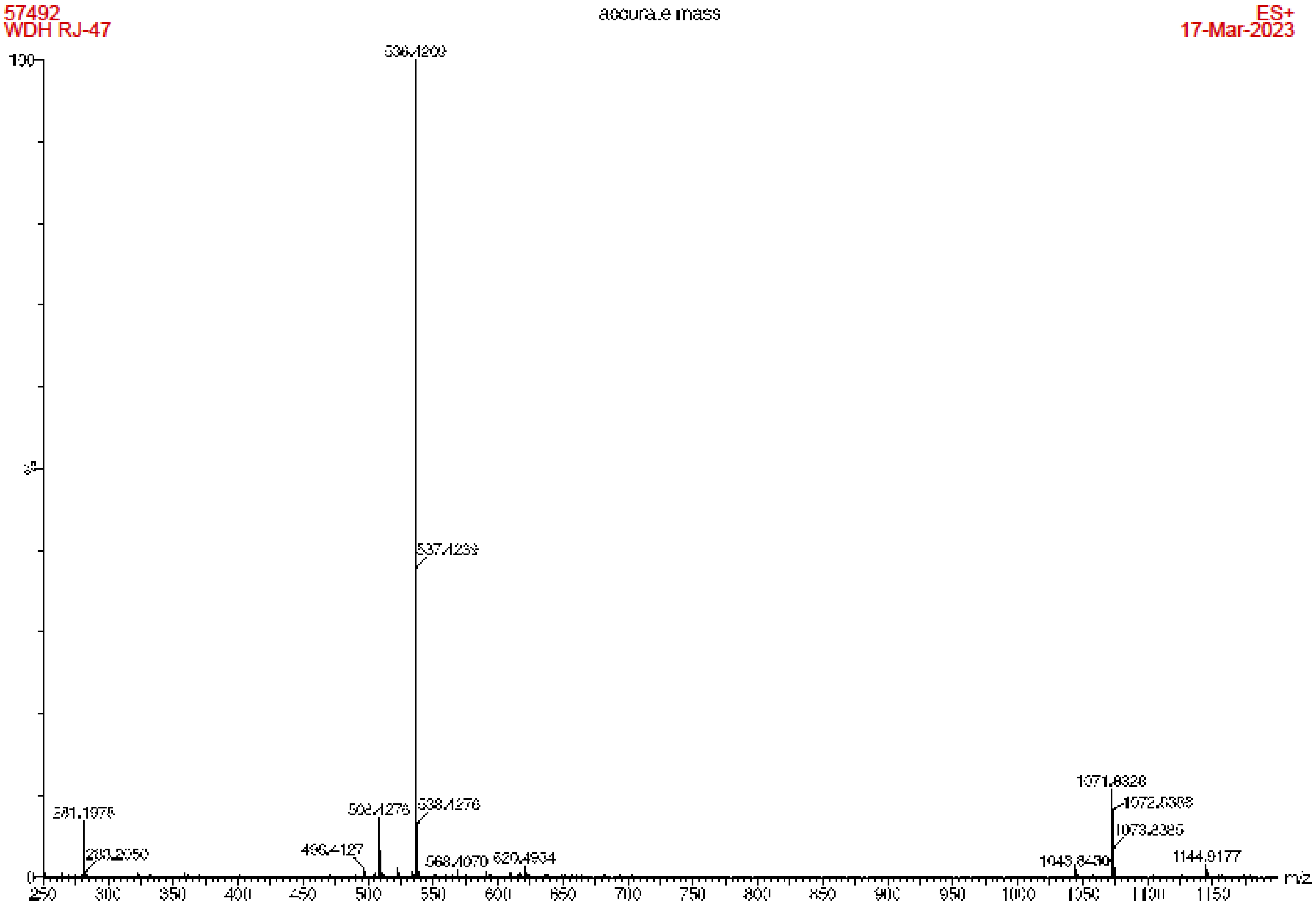

Compound **11** (MS)

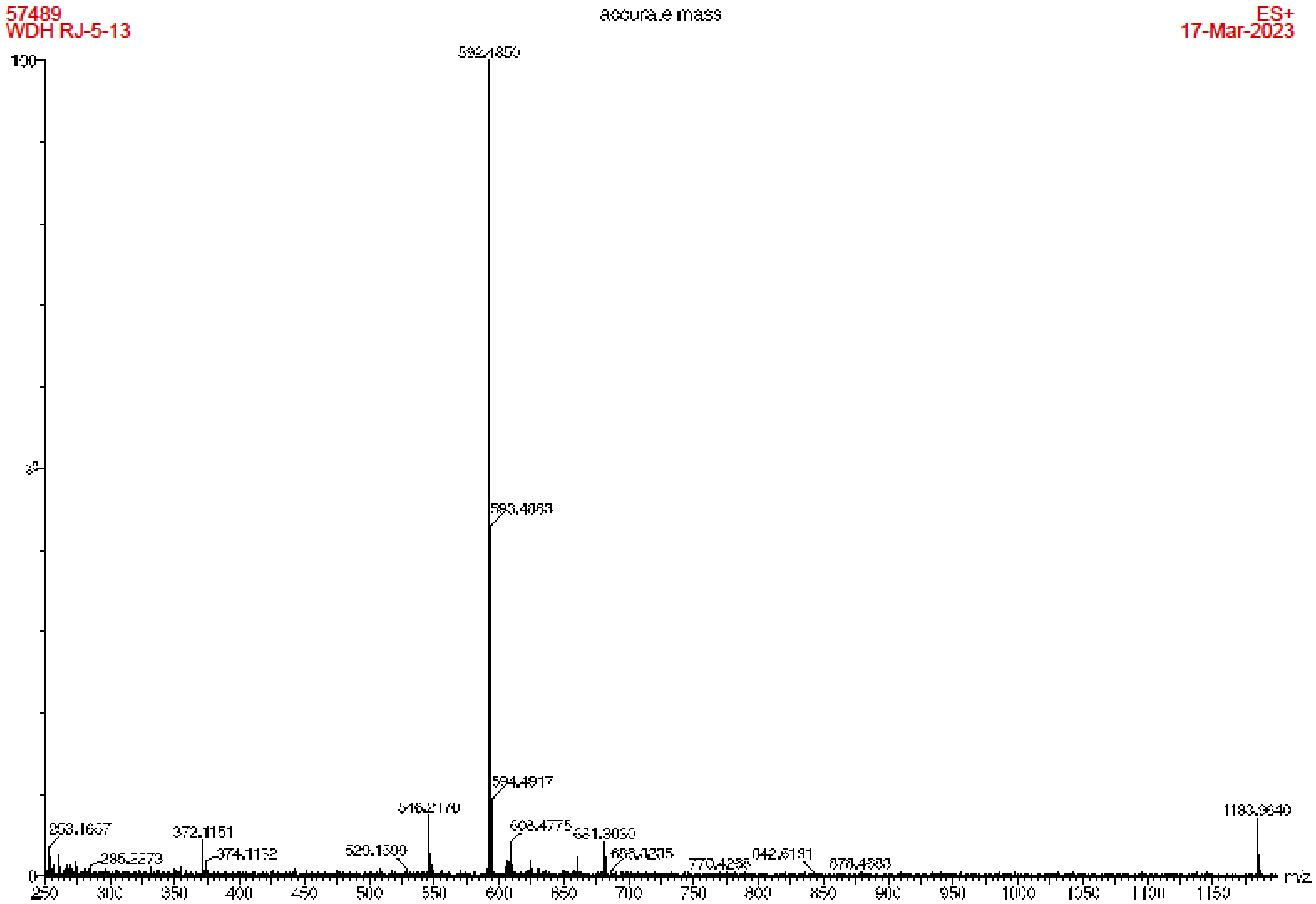

Compound **12** (MS)

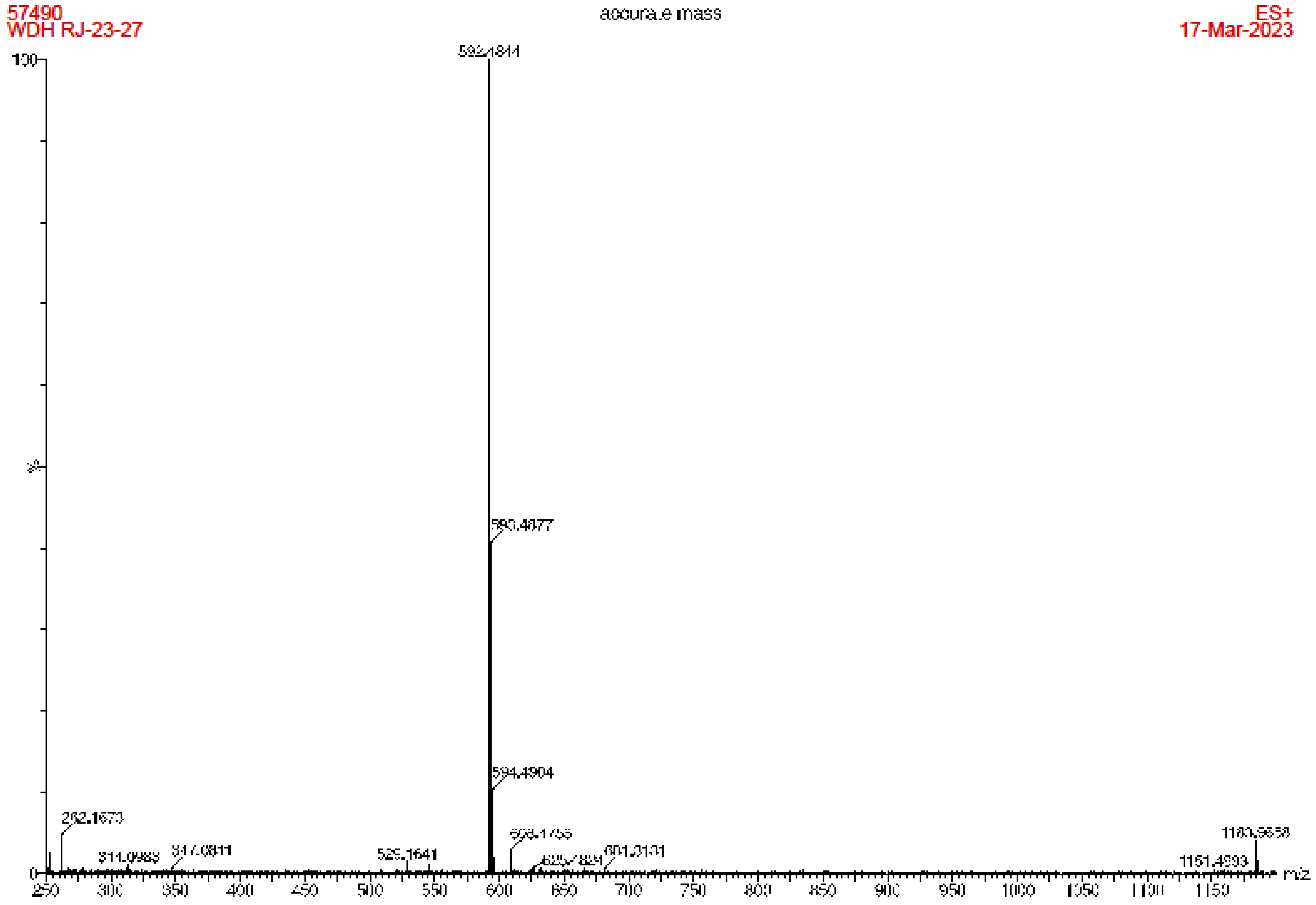

Compound **13** (MS)

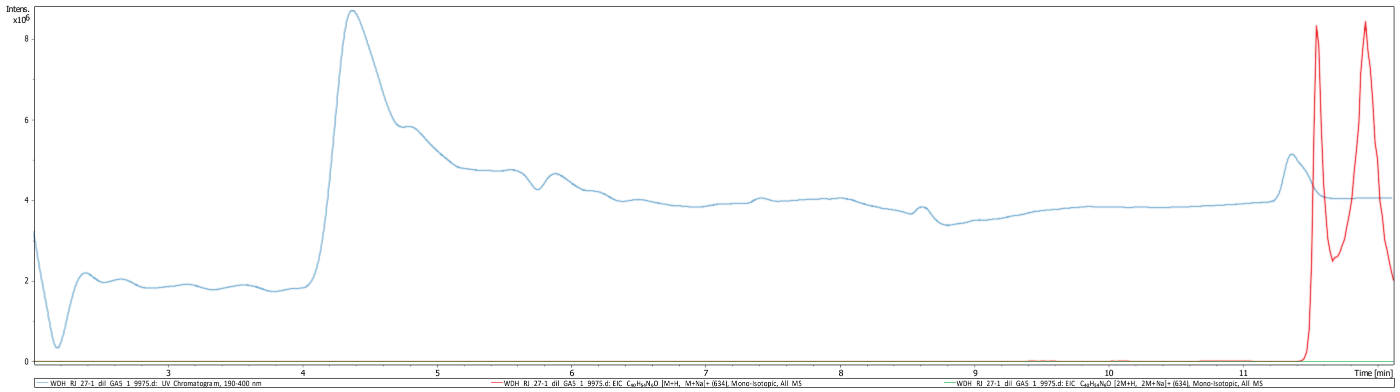

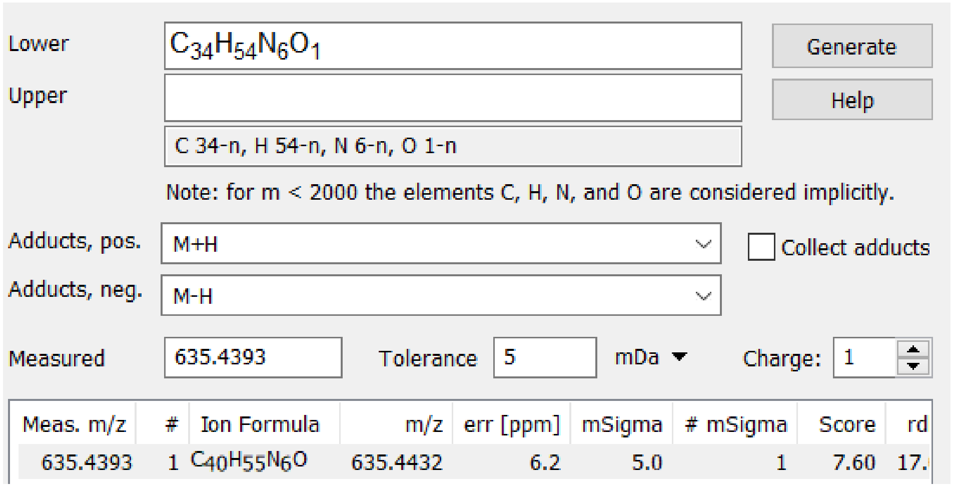

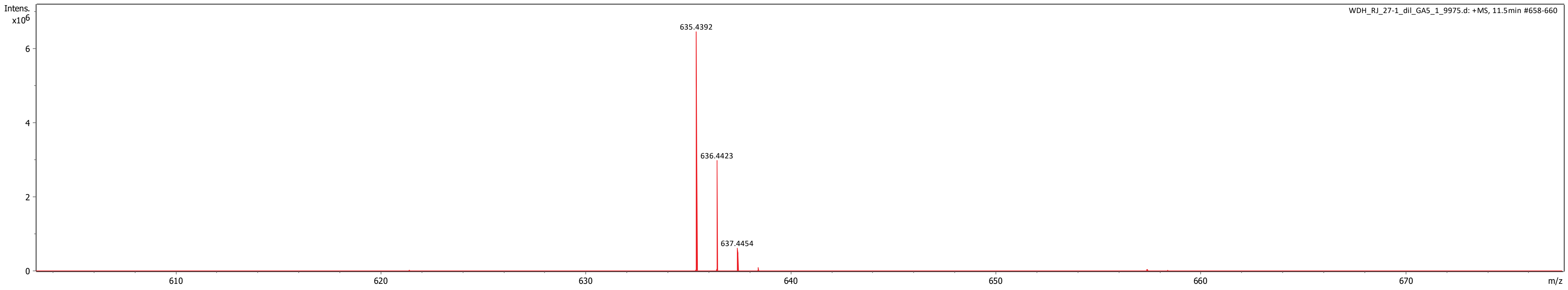

Compound **14** (MS)

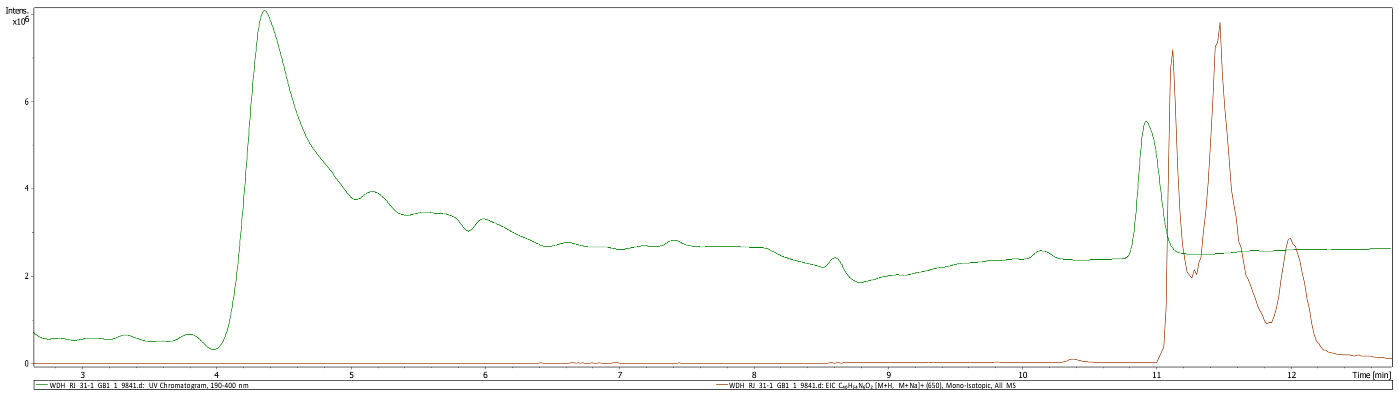

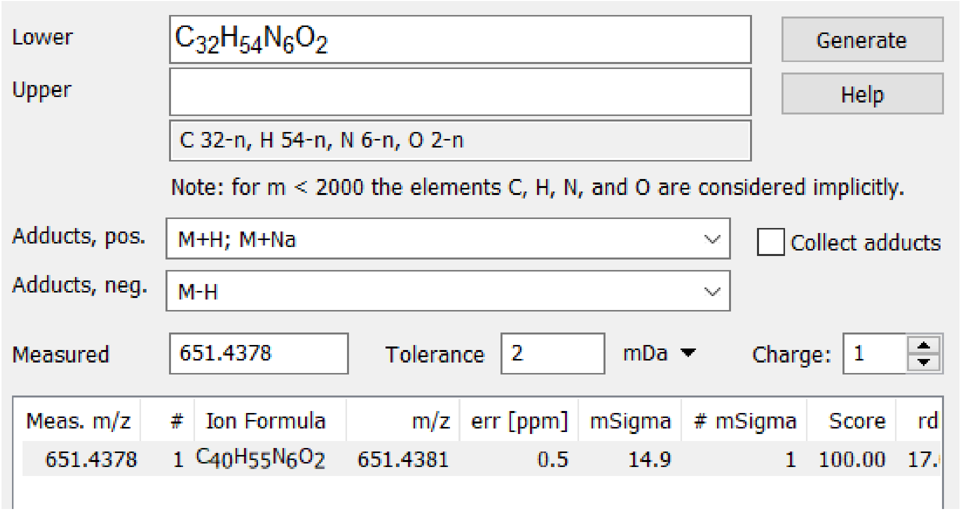

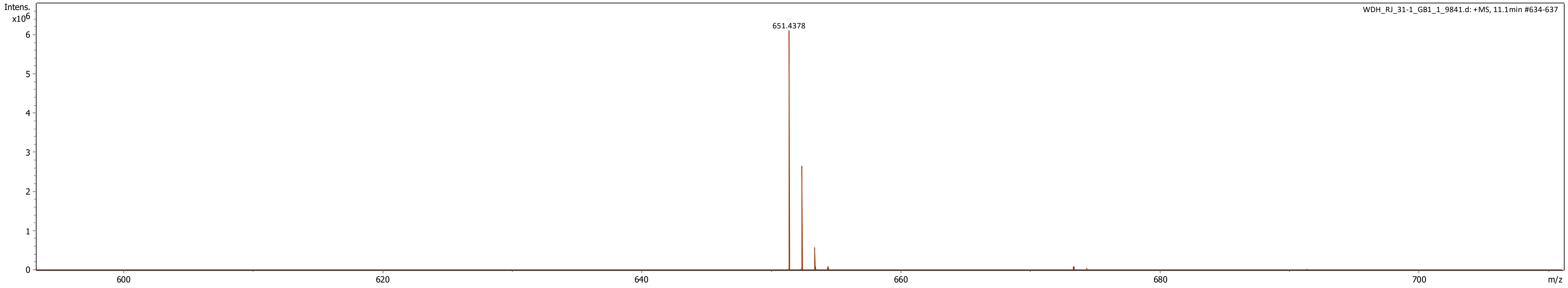

Compound **15** (MS)

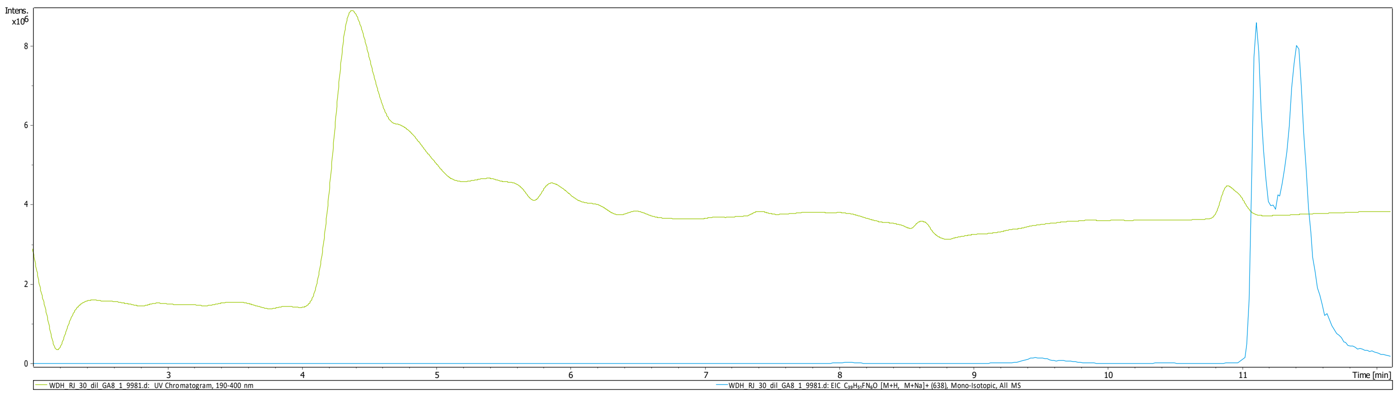

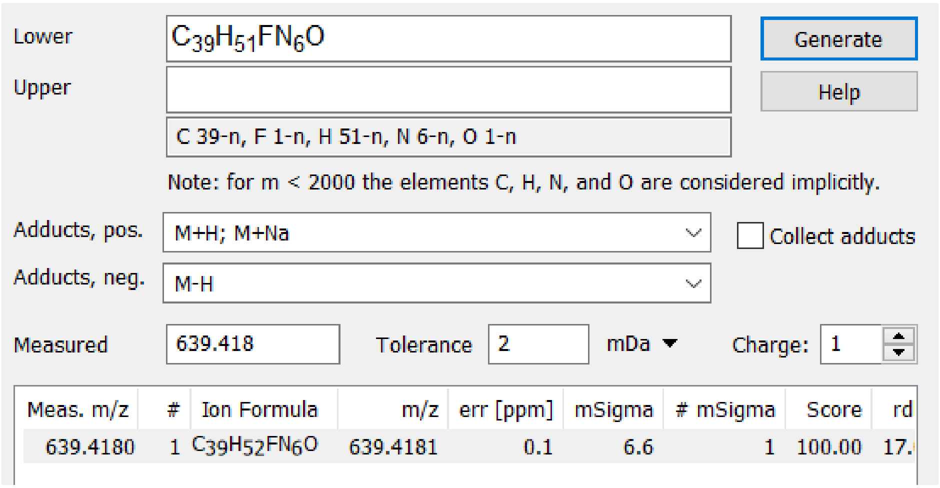

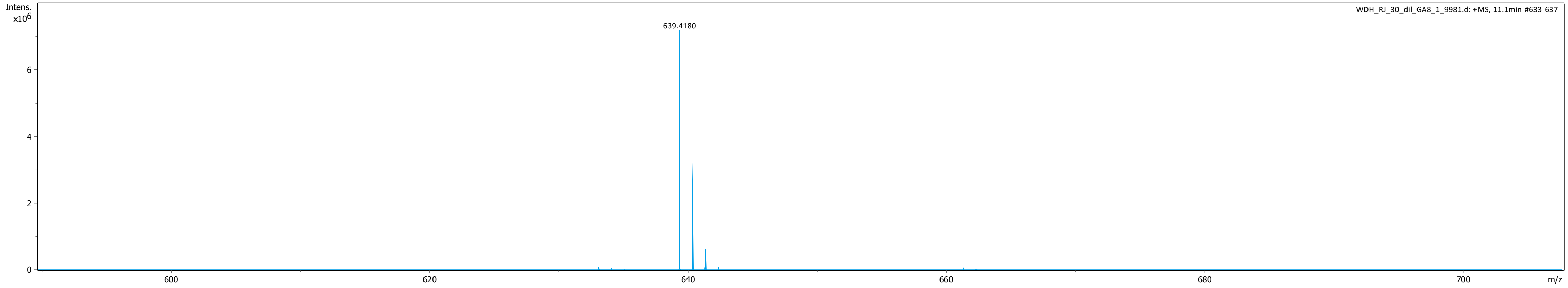

Compound **16** (MS)

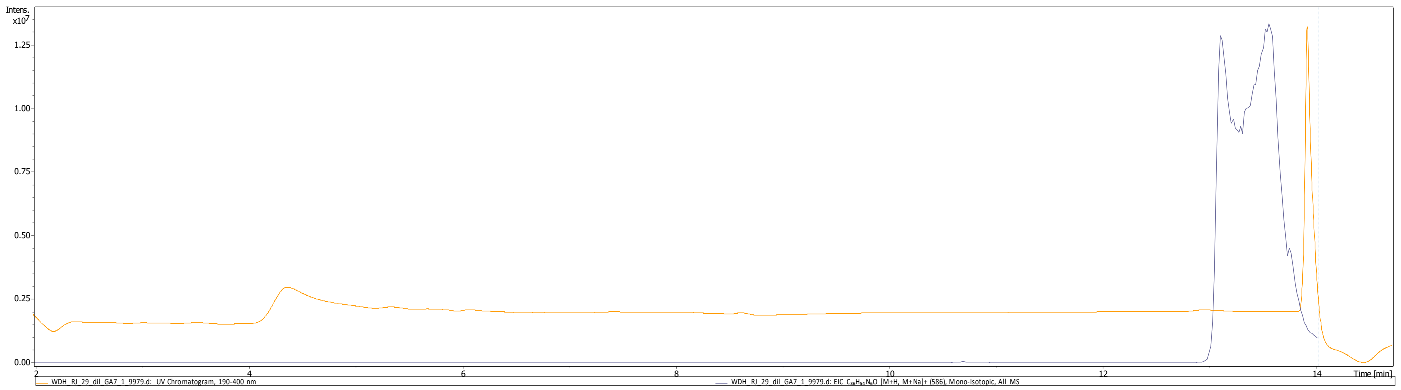

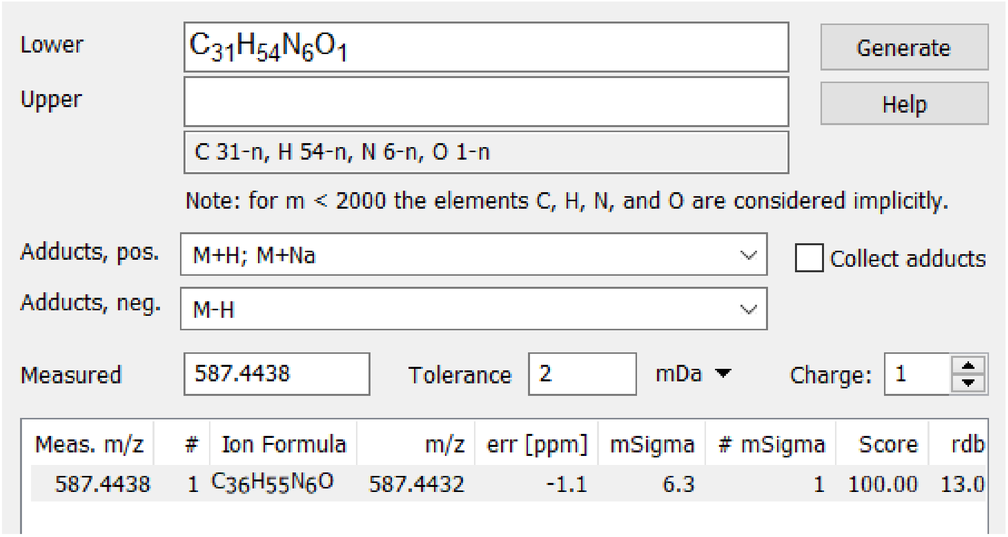

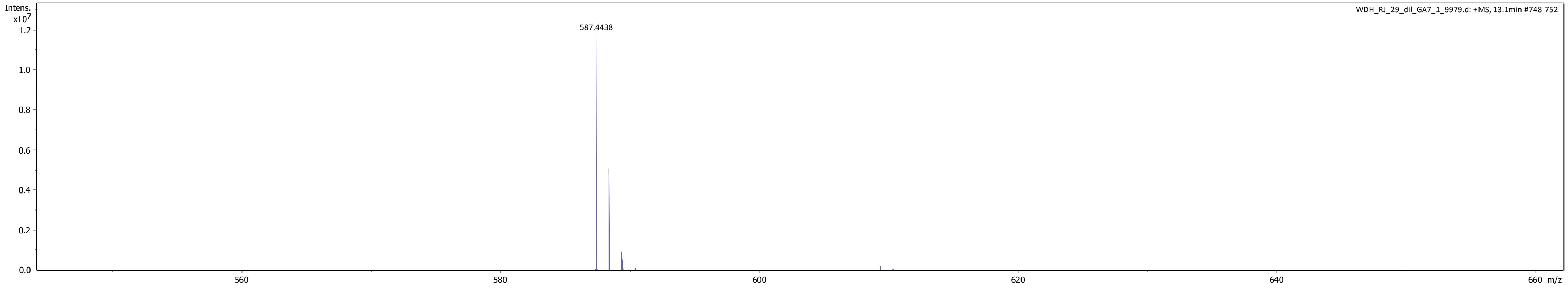

Compound **17** (MS)

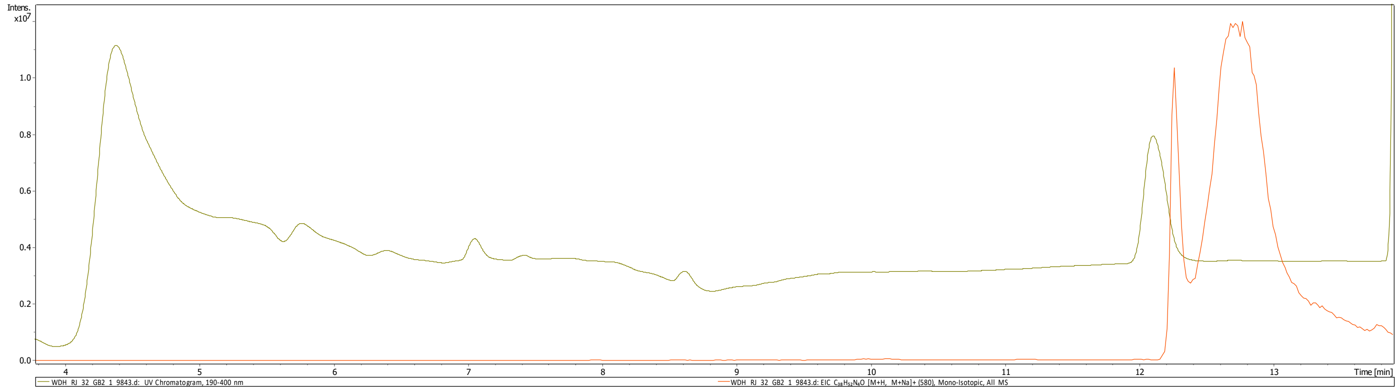

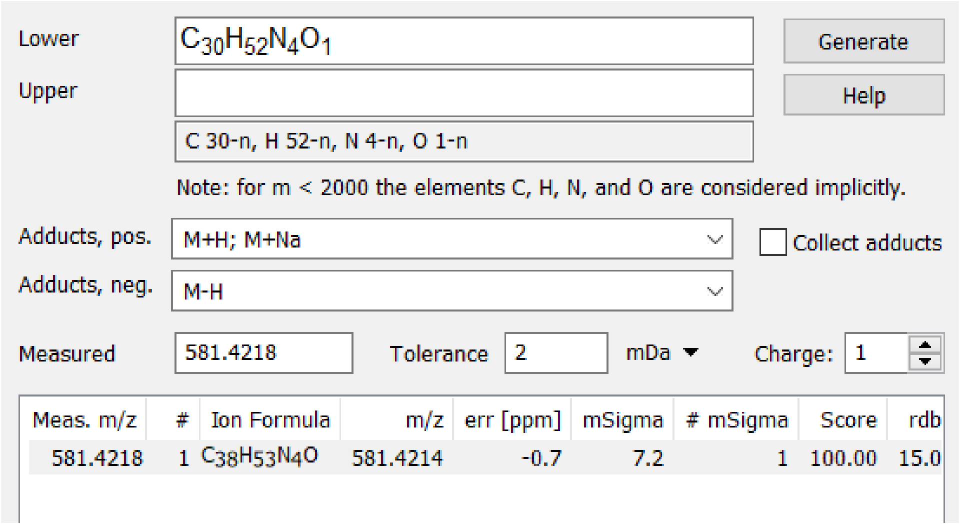

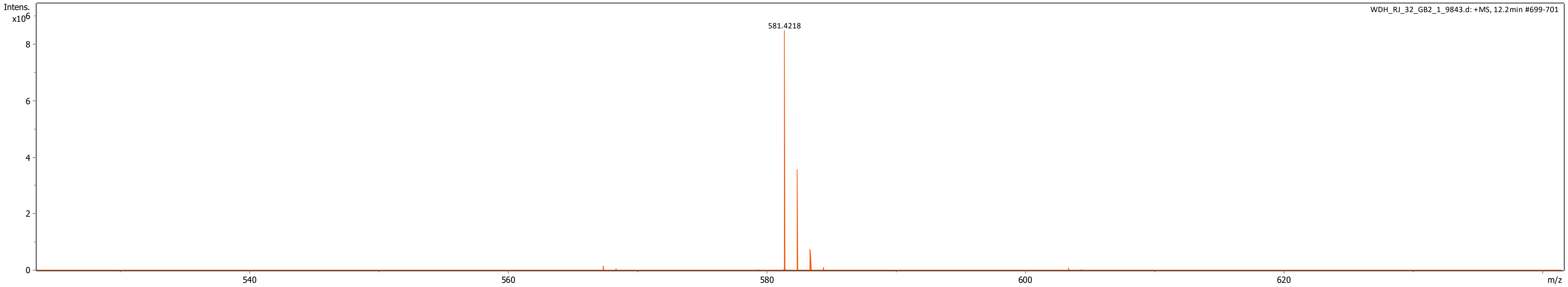

Compound **18** (MS)

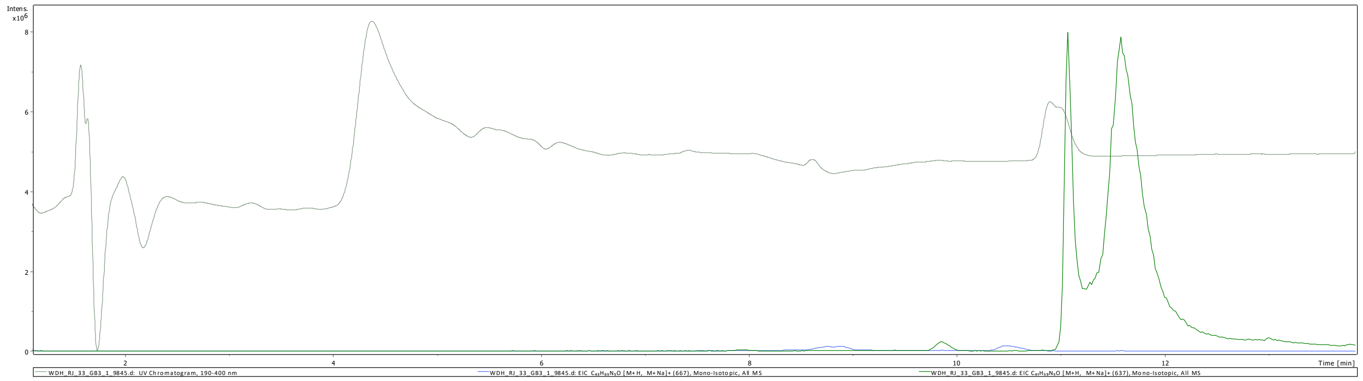

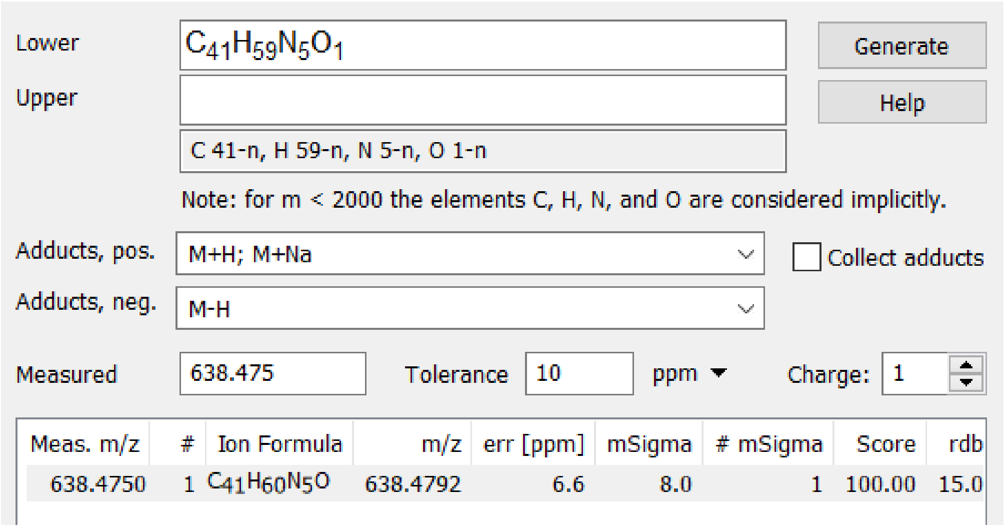

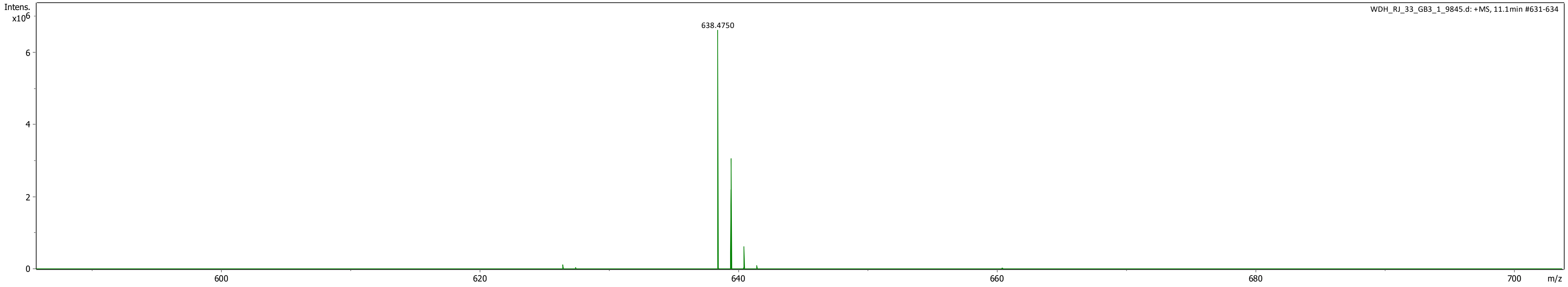

Compound **19** (MS)

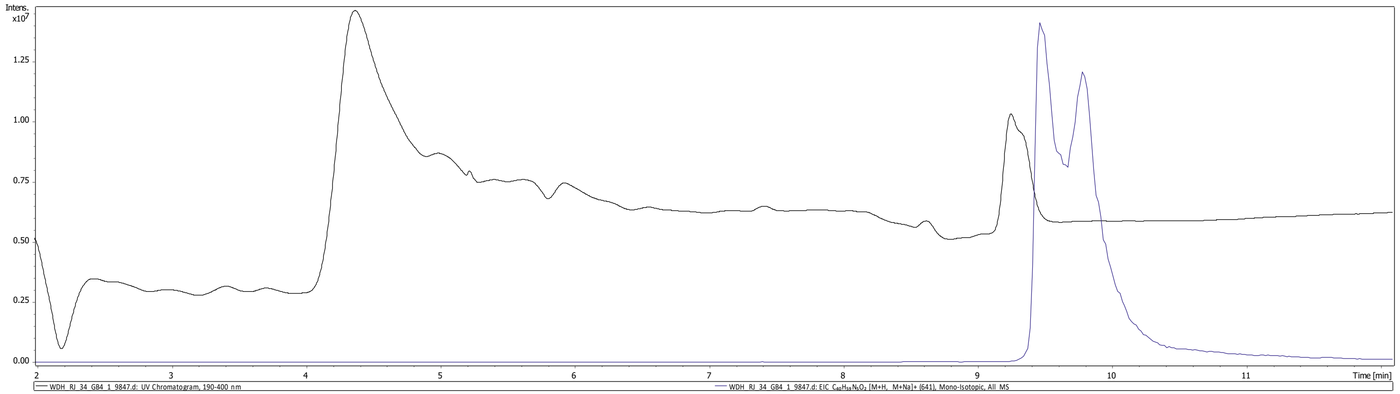

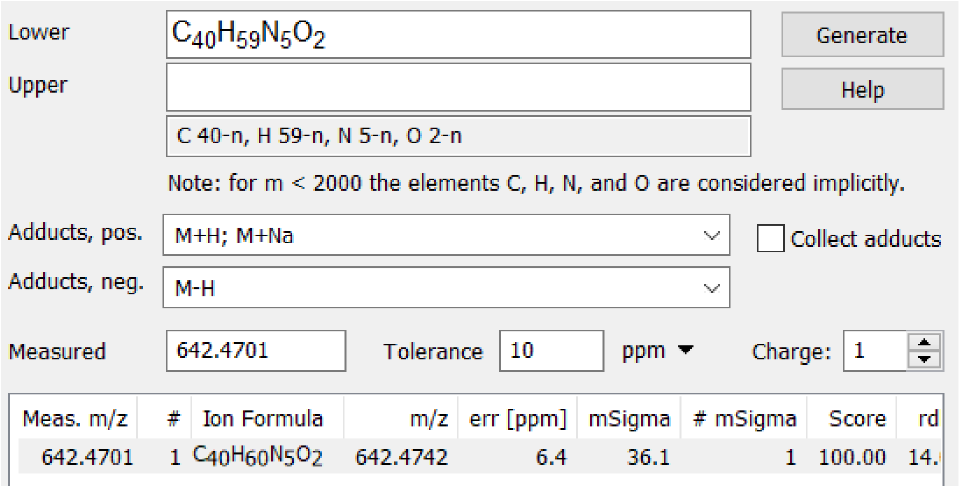

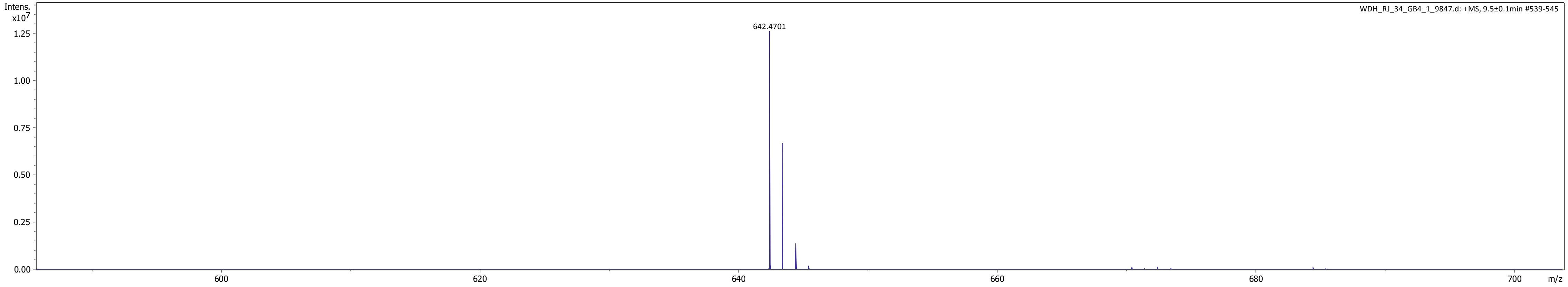

Compound **20** (MS)

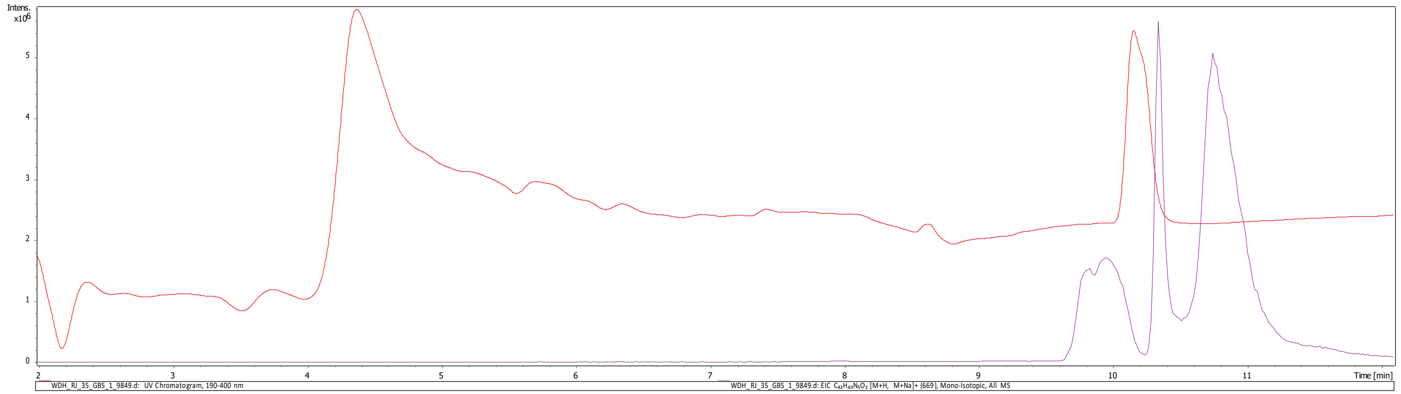

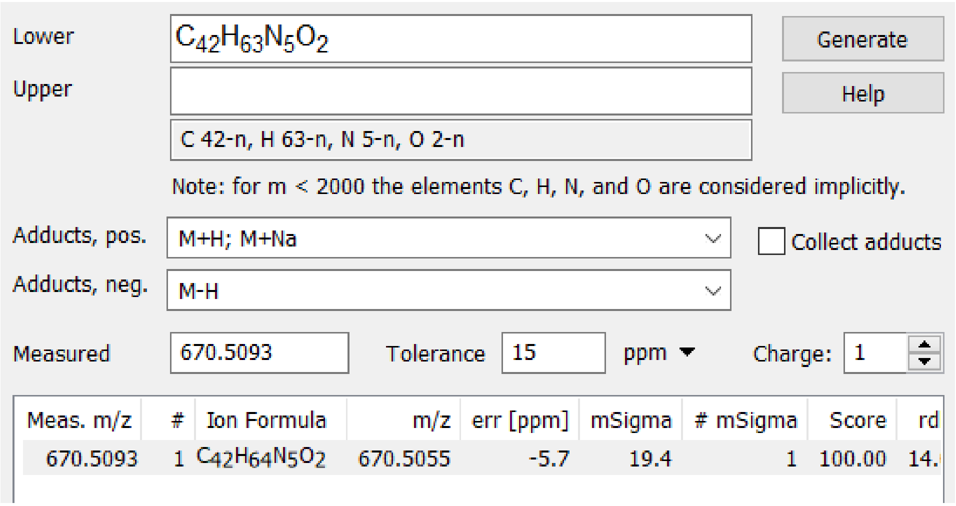

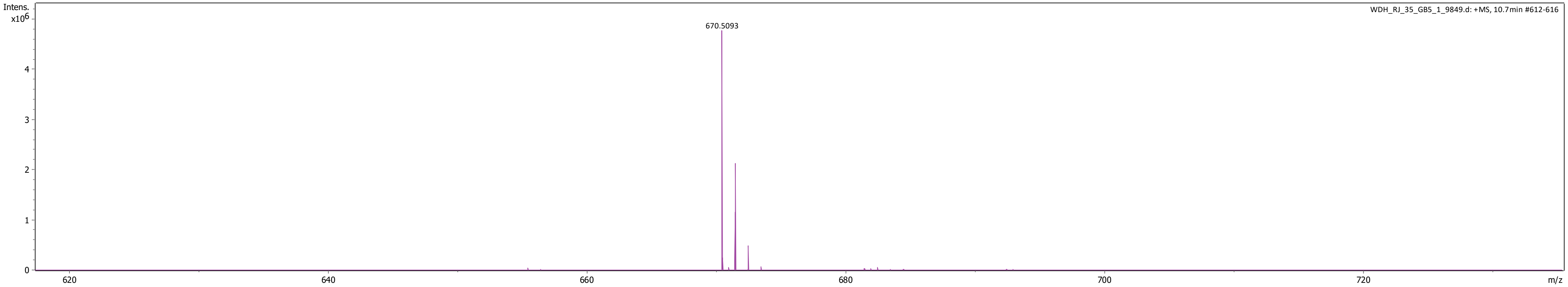

Compound **21** (MS)

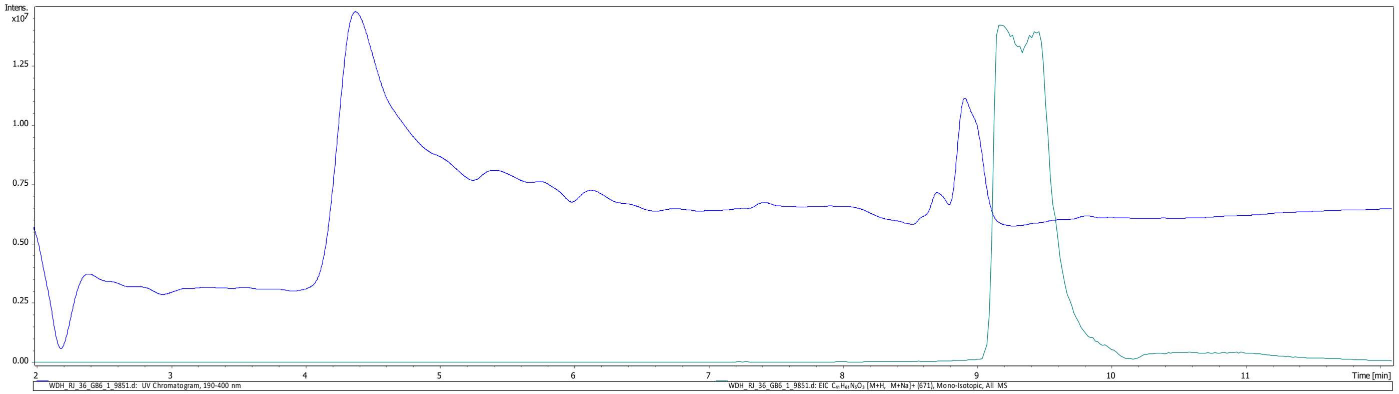

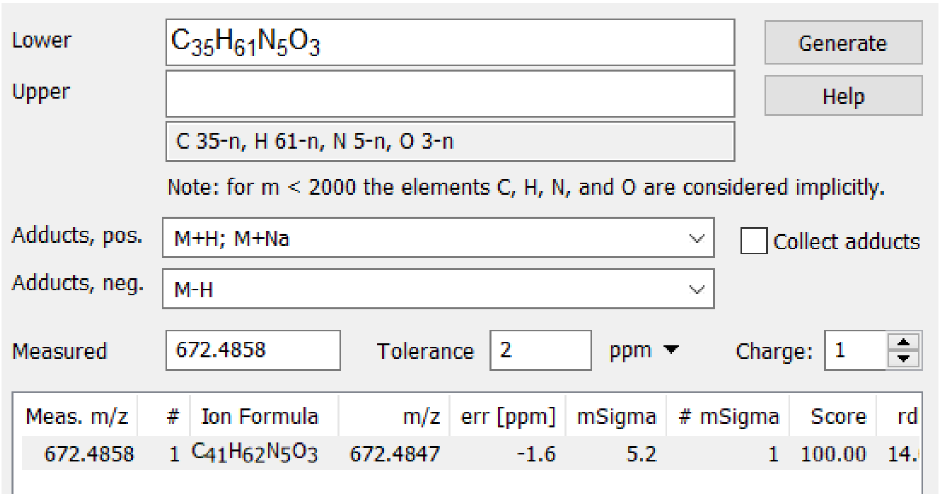

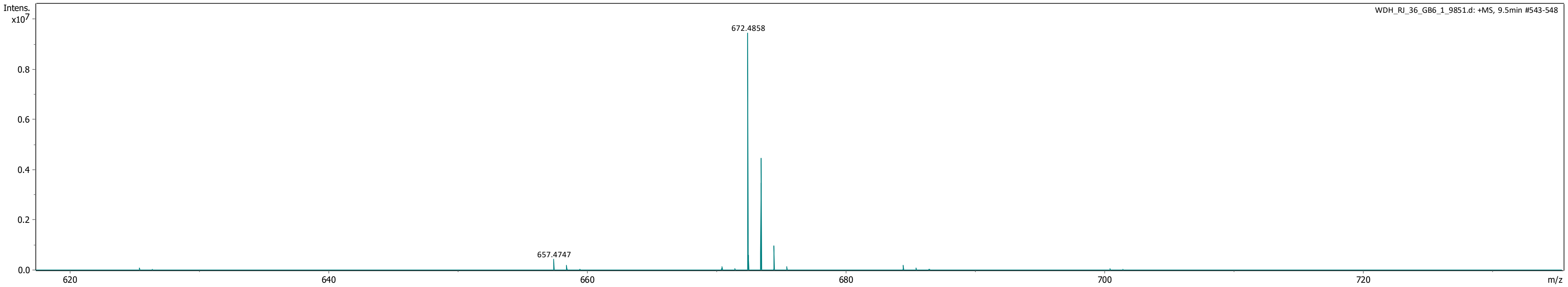

Compound **22** (MS)

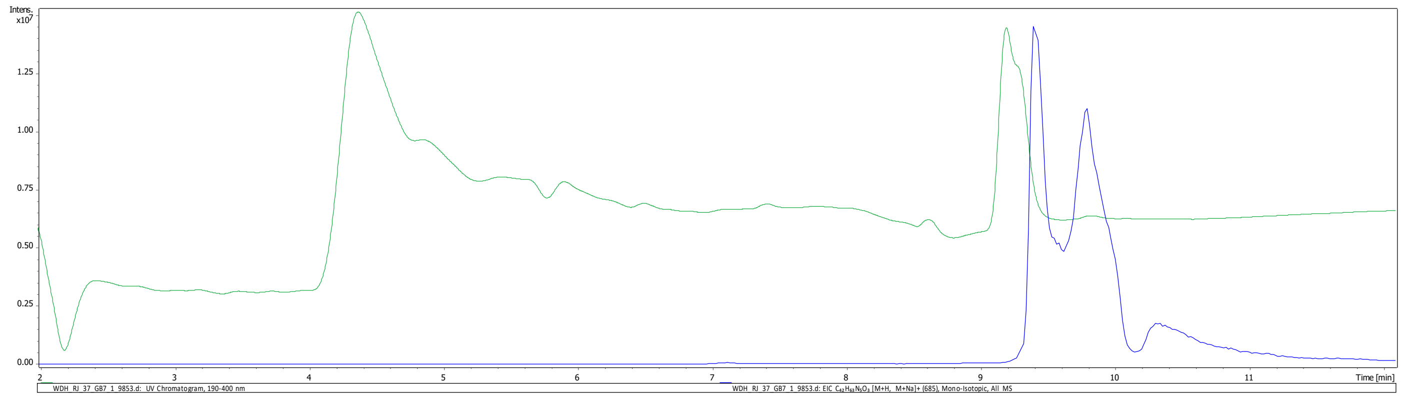

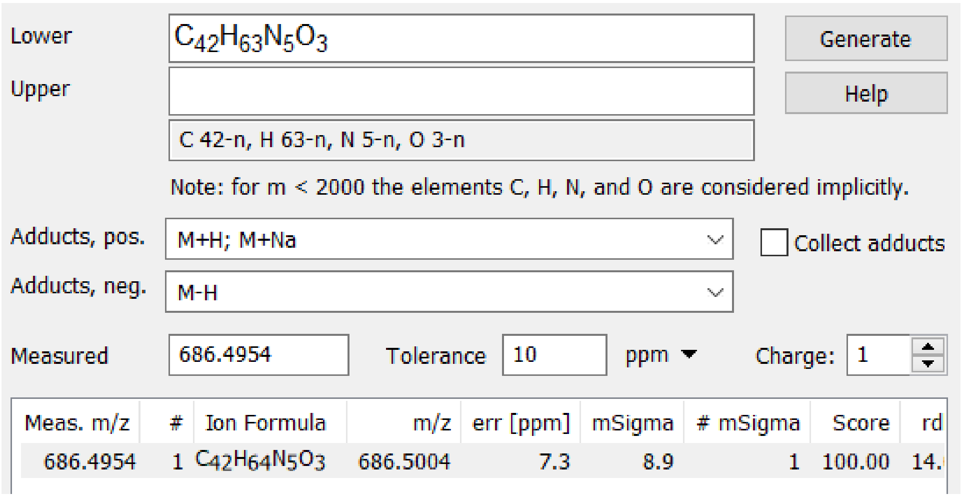

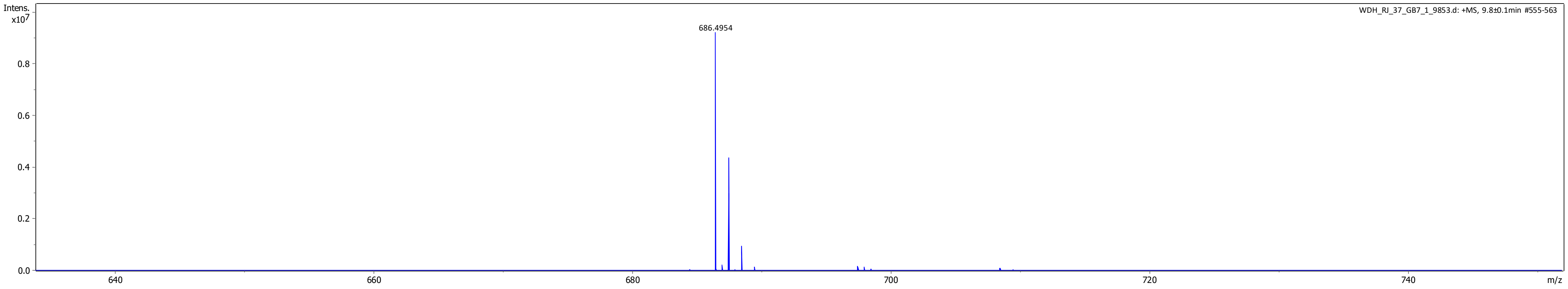

Supplement: Supplementary file 1 [file molecules-29-03149-s001.zip › molecules-3069907-supplementary.pdf]
